# Structural insights into coordinating 5S RNP rotation with ITS2 pre‐RNA processing during ribosome formation

**DOI:** 10.15252/embr.202357984

**Published:** 2023-11-03

**Authors:** Matthias Thoms, Benjamin Lau, Jingdong Cheng, Lisa Fromm, Timo Denk, Nikola Kellner, Dirk Flemming, Paulina Fischer, Laurent Falquet, Otto Berninghausen, Roland Beckmann, Ed Hurt

**Affiliations:** ^1^ Gene Center Ludwig‐Maximilians‐Universität München Munich Germany; ^2^ Heidelberg University Biochemistry Center (BZH) Heidelberg Germany; ^3^ Minhang Hospital & Institutes of Biomedical Sciences, Shanghai Key Laboratory of Medical Epigenetics Fudan University Shanghai China; ^4^ University of Fribourg and Swiss Institute of Bioinformatics Fribourg Switzerland

**Keywords:** 5S RNP, ITS2, pre‐60S ribosome, ribosome biogenesis, rixosome, Structural Biology, Translation & Protein Quality

## Abstract

The rixosome defined in *Schizosaccharomyces pombe* and humans performs diverse roles in pre‐ribosomal RNA processing and gene silencing. Here, we isolate and describe the conserved rixosome from *Chaetomium thermophilum*, which consists of two sub‐modules, the sphere‐like Rix1‐Ipi3‐Ipi1 and the butterfly‐like Las1‐Grc3 complex, connected by a flexible linker. The Rix1 complex of the rixosome utilizes Sda1 as landing platform on nucleoplasmic pre‐60S particles to wedge between the 5S rRNA tip and L1‐stalk, thereby facilitating the 180° rotation of the immature 5S RNP towards its mature conformation. Upon rixosome positioning, the other sub‐module with Las1 endonuclease and Grc3 polynucleotide‐kinase can reach a strategic position at the pre‐60S foot to cleave and 5′ phosphorylate the nearby ITS2 pre‐rRNA. Finally, inward movement of the L1 stalk permits the flexible Nop53 N‐terminus with its AIM motif to become positioned at the base of the L1‐stalk to facilitate Mtr4 helicase‐exosome participation for completing ITS2 removal. Thus, the rixosome structure elucidates the coordination of two central ribosome biogenesis events, but its role in gene silencing may adapt similar strategies.

## Graphical abstract

The rixosome is involved in ribosome biogenesis and gene silencing. Its cryo‐EM structure explains how it can participate in pre‐60S ribosome maturation during 5S RNP rotation and ITS2 processing.

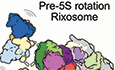

## Introduction

The eukaryotic ribosome is a macromolecular machinery consisting of a large and a small subunit and is responsible for all protein synthesis in living cells. Rapidly dividing cells have a high demand for correctly assembled ribosomes, which are ready to engage in accurate translation of the genetic code. The assembly line providing mature eukaryotic ribosomes starts in the nucleolus with the transcription of a large precursor ribosomal RNA (rRNA), 35S in yeast and 47S in human. This precursor is transcribed by RNA polymerase I and contains the 18S rRNA of the 40S small ribosomal subunit and the 5.8S and 25S rRNAs of the 60S large subunit, connected by two internal transcribed spacers (ITS1 and ITS2) and flanked by two external transcribed spacers (5′ ETS and 3′ ETS), respectively, which are successively processed and removed by endo‐ and exo‐nucleases (Henry *et al*, 1994; Woolford & Baserga, 2013; Bassler & Hurt, 2019; Klinge & Woolford, 2019).

The multiple maturation processes of the pre‐ribosomes require processing, folding and modification of the rRNA molecules as well as incorporation of around 80 ribosomal proteins. Numerous assembly factors (~200) promote and safe‐guard this process, facilitating favorable RNA and protein folding states including quality control mechanisms as maturation proceeds. Already co‐transcriptionally, a myriad of assembly factors associates with the nascent rRNA driving maturation (Dragon *et al*, 2002; Grandi *et al*, 2002; Bassler & Hurt, 2019; Klinge & Woolford, 2019). Cryo‐electron microscopy (cryo‐EM) studies of the first biochemically stable pre‐ribosomal intermediate, the huge 90S particle, isolated from yeast, *Chaetomium thermophilum*, and human cells, revealed how its molecular structure provides a dynamic scaffold, which enables the timely hierarchical folding of the pre‐18S rRNA and assembly of ribosomal proteins (Kornprobst *et al*, 2016; Cheng *et al*, 2017; Sun *et al*, 2017; Du *et al*, 2020; Singh *et al*, 2021). Noteworthy, after endo‐nucleolytic cleavage at site A_2_ in the ITS1, the maturation pathways of the small and large subunit proceed independently from each other, while the pre‐ribosomes travel through the nucleolus and nucleoplasm (Udem & Warner, 1972; Woolford & Baserga, 2013; Bassler & Hurt, 2019; Klinge & Woolford, 2019). In addition to the 5.8S and the 25S rRNA, the 60S large subunit contains the 5S rRNA, which is transcribed separately by RNA polymerase III and incorporated as 5S RNP early during pre‐60S maturation together with the ribosomal proteins uL18 (rpL5), uL5 (rpL11), and assembly factors Rrs1 and Rpf2 (Zhang *et al*, 2007; Asano *et al*, 2015; Kharde *et al*, 2015; Madru *et al*, 2015; Castillo Duque de Estrada *et al*, 2023; Lau *et al*, 2023). After export through the nuclear pore complexes, a final quality control checkpoint probes the integrity and reliability of the nascent ribosomal subunits in the cytoplasm (Bassler & Hurt, 2019; Klinge & Woolford, 2019).

Early nucleolar assembly intermediates of the pre‐60S maturation pathway are characterized by the presence of 27SA2 rRNA and the trimmed form 27SB pre‐rRNA. These early intermediates could be elucidated by cryo‐EM and revealed the initial folding of rRNA domains, and association of first assembly factors and ribosomal proteins (Tsuno *et al*, 2000; Granneman *et al*, 2011; Kater *et al*, 2017; Sanghai *et al*, 2018; Zhou *et al*, 2019; Lau *et al*, 2023). Later, one decisive step in nucleolar pre‐60S biogenesis is the release of the Erb1‐Ytm1 heterodimer by the AAA‐ATPase Rea1, which triggers the dissociation of interdependent assembly factors, associated with Erb1's N‐terminus (Mitterer *et al*, 2023). Consequently, structural rearrangements and the repositioning of the L1 stalk are induced (Miles *et al*, 2005; Kressler *et al*, 2008; Thoms *et al*, 2016; Sanghai *et al*, 2018; Kater *et al*, 2020). In further matured nucleoplasmic pre‐60S intermediates, the Rea1‐Rix1 machinery has to be correctly positioned and stably assembled for Rea1 to release its second target substrate Rsa4, a structural homolog of Ytm1 (Ulbrich *et al*, 2009; Bassler *et al*, 2010; Leidig *et al*, 2014; Wu *et al*, 2016; Kater *et al*, 2020). Both proteins consist of a WD40 repeat β‐propeller domain with an N‐terminal extension, containing a ubiquitin‐like (UBL) domain interacting by a conserved mechanism with the C‐terminal MIDAS domain of Rea1. Thus, specific mutations in the UBL domains of Ytm1 and Rsa4 cause a lethal phenotype in yeast (Ulbrich *et al*, 2009; Bassler *et al*, 2010; Thoms *et al*, 2016). Considering the enormous size and complexity of the Rea1‐Rix1 machinery and its many contact points on the pre‐60S particle, this process serves as an important quality control mechanism, integrating the final conformational rotation of the 5S rRNA into its mature position, dissociation of Rpf2‐Rrs1 from the pre‐60S particle and Rsa4 release as an exit of the checkpoint control and clearance for progression toward the export‐competent pre‐60S particle (Barrio‐Garcia *et al*, 2016; Kater *et al*, 2020; Micic *et al*, 2020).

During ribosome biogenesis, an often‐observed strategy is the transitory stabilization of rRNA domains in distinct premature conformations in order to avoid misfolding or interference with the maturation of other pre‐rRNA domains. A prominent example is a unique pre‐mature 5S rRNA arrangement in nucleolar pre‐60S particles, which eventually requires a 180° rotation of the entire 5S RNP into its mature conformation during a distinct nucleoplasmic maturation step. This rotation is thought to be triggered by the Rea1 AAA ATPase and terminated by Rsa4 removal, and was suggested to be timely coordinated with the processing and removal of the ITS2, a spacer RNA inserted between 5.8S and 25S rRNAs. Importantly, this pre‐rRNA processing step is initiated by the Las1‐Grc3 endo‐nuclease complex and completed by the Rat1‐Rai1 5′ > 3′ exonuclease and Mtr4‐exosome for 3′ > 5′ ITS2 degradation (Gasse *et al*, 2015; Thoms *et al*, 2015; Fromm *et al*, 2017; Pillon *et al*, 2017; Schuller *et al*, 2018). Yet, the underlying mechanism of coordinating these different maturation steps is largely unclear.

Here, we report the usage of the ribosome assembly factor Rsa4 from *C. thermophilum*, converted into a dominant‐negative mutant (*ct*Rsa4 E117D) to block interaction with the Rea1‐MIDAS domain, in order to purify nuclear pre‐60S intermediates accumulating in the nucleoplasm. The obtained series of pre‐60S particles was subjected to cryo‐EM analysis, which to our surprise exhibited the Rix1 complex in states before and after the 5S RNP rotation, pointing to a direct role of the Rix1 complex in triggering 5S RNP rotation on the pre‐60S independent of the Rea1 ATPase. Subsequent isolation of the wild‐type Las1‐Grc3 complex, known to initiate ITS2 processing in yeast (see above), from *C. thermophilum* uncovered a stoichiometric association with the *ct* Rix1 complex. Notably, the Rix1 complex and the Las1‐Grc3 were previously described in *Schizosaccharomyces pombe* and humans as being part of the “rixosome” that has a second role in gene silencing at heterochromatin (Shipkovenska *et al*, 2020; Zhou *et al*, 2022). Thus, we analyzed the purified *ct* rixosome by cryo‐EM, which uncovered its structural organization into the spherical Rix1‐Ipi3‐Ipi1 complex and the flexibly attached Las1‐Grc3 sub‐module but in distinct orientation toward the Rix1 complex. Based on these findings, we were able to propose a model of how the Rix1 complex can trigger 5S RNP rotation, whereas the flexibly attached Las1‐Grc3 endonuclease sub‐module becomes strategically positioned on the pre‐60S for its role in ITS2 processing.

## Results

### Dominant‐negative *Chaetomium thermophilum rsa4 E117D* induces a growth defect with pre‐60S accumulation

In the past, we were able to purify and structurally characterize several 90S pre‐ribosomal particles as part of the 40S biogenesis pathway from *C. thermophilum* (Kornprobst *et al*, 2016; Cheng *et al*, 2017, 2019), but failed to isolate pre‐60S particles by affinity‐purification of epitope‐tagged assembly factors. Recently, however, we circumvented this problem by affinity‐purification of a mutant pre‐60S bait, *ct* Ytm1E88D, which provided us with a number of nucleolar pre‐60S intermediates (Lau *et al*, 2023).

In order to isolate nucleoplasmic pre‐60S particles from *C. thermophilum*, we expressed another mutant pre‐60S factor, *ct rsa4 E117D*, based on findings in yeast (*S. cerevisiae*, *sc*), where overexpression of the homologous *sc rsa4 E114D* mutation induced a dominant‐lethal phenotype with arrest in nucleoplasmic 60S biogenesis (Ulbrich *et al*, 2009). For our purpose, we tagged the *ct rsa4 E117D* allele with ProtA‐TEV‐Flag (PTF), placed it under the control of the constitutive actin promoter and connected the gene construct to the *erg1* selection marker (Kellner *et al*, 2016). When protoplasts were transformed not only with *PTF‐rsa4 E117D* but also with a wild‐type *PTF‐RSA4* construct for a mock control, terbinafine‐resistant colonies could be selected, however, *PTF‐rsa4 E117D* expressing mycelium grew significantly slower than wild‐type *PTF‐RSA4* (see below).

**Figure 1:**
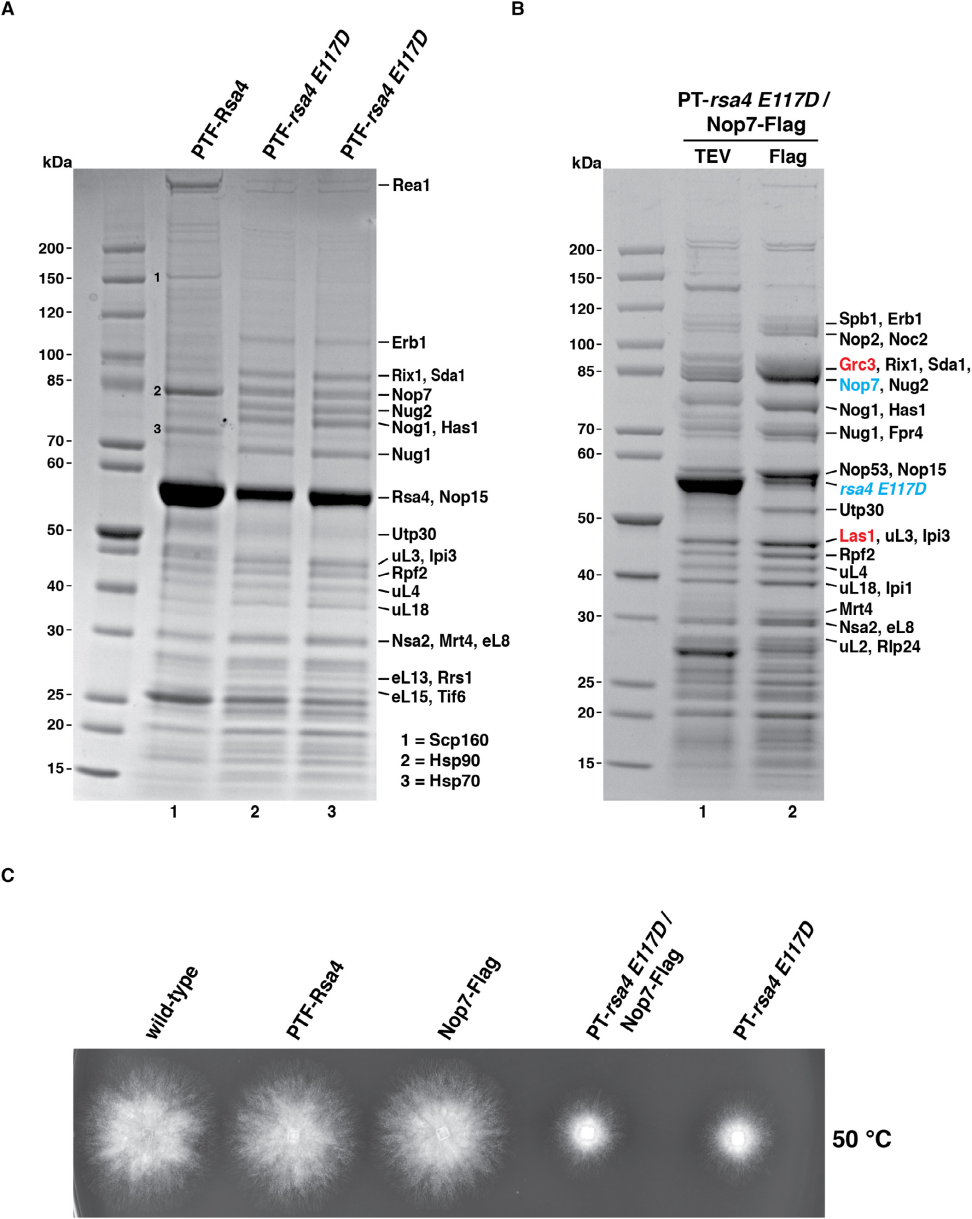
The dominant‐negative *C. thermophilum rsa4 E117D* mutant allows isolation of pre‐60S particles Affinity‐purification of wild‐type PTF‐Rsa4 (lane 1) and the PTF‐Rsa4 E117D mutant from *C. thermophilum* lysates (lane 2 and 3; two different transformants). The final eluates were analyzed by SDS–PAGE and Coomassie staining. The labeled bands were identified by mass spectrometry. In the case of wild‐type PTF‐Rsa4, Scp160 (band 1), Hsp90 (band 2), and Hsp70 (band 3) were identified by mass spectrometry as possible contaminants. The PTF‐Rsa4 wt purifies predominantly mature ribosomes whereas the PTF‐Rsa4 E117D mainly purifies pre‐60S particles.The split‐pair combination PT‐Rsa4 E117D and Nop7‐Flag was used to affinity‐purify pre‐60S particles from *C. thermophilum* lysates. Both the first TEV eluate (lane 1) and second Flag eluate (lane 2) were analyzed by SDS–PAGE and Coomassie staining. Bands labeled on the right were identified by mass spectrometry, with bait proteins labeled in blue and Las1‐Grc3 complex in red, respectively. The affinity‐purifications have been performed at least twice with similar outcome.Growth analysis of ectopically expressed *RSA4* wild‐type and *rsa4 E117D* mutant genes in *C. thermophilum*. Small mycelium pieces of similar size, containing the indicated pre‐grown *C. thermophilum* strains, were plated on CCM agar and mycelium growth was analyzed after 13 h at 50°C. Source data are available online for this figure.

To test which types of pre‐60S particles enrich in this *rsa4* mutant, *ct* PTF‐Rsa4 E117D was affinity‐purified from mycelium lysate, which showed a co‐enrichment of a number of typical pre‐60S factors known from yeast, but the wild‐type Rsa4 eluate was largely devoid of these components. However, the wild‐type Rsa4 not only co‐purified its direct binding partner Rea1 but also heat shock proteins and ribosomal proteins, which are frequent contaminants during affinity‐purifications (Fig 1A).

To improve and confine the purification of such pre‐60S particles, we considered split‐tag affinity‐purification. Hence, we generated a thermophile transformant harboring not only the mutant ProtA‐TEV‐tagged (PT) Rsa4 E117D (called strain pChaetomium1) but also a second epitope‐tagged wild‐type pre‐60S factor, Nop7‐Flag (pChaetomium2) (see Materials and Methods). Double transformants expressing these two tagged baits were not only identified by Western blot but also subjected to whole genome sequencing. This revealed random insertion of *PT‐rsa4 E117D* and *NOP7‐Flag* at different genome loci, but each with only one copy per genome (Appendix Fig S1). Based on this sequencing data, we could further assemble an improved genome sequence of wild‐type *C. thermophilum*, established on the currently available reference genome (see Materials and Methods). When analyzed for mycelium growth, the PT‐*rsa4 E117D* strain and the *PT‐rsa4 E117D NOP7‐Flag* split‐tag strain grew significantly slower than the wild‐type *PT‐RSA4* or the single *NOP7‐Flag* strain (Fig 1C), confirming that constitutive expression of *ct rsa4 E117D* causes slow growth but not a lethal phenotype in *C. thermophilum*.

Next, the split‐tag *PT‐rsa4 E117D NOP7‐Flag* strain was subjected to tandem affinity‐purification (Cheng *et al*, 2019), which showed a co‐enrichment of more than 20 conserved pre‐60S factors in the final Flag eluate, among several GTPases (Nog1, Nog2, Nug1), all members of the Rix1 complex (Rix1‐Ipi3‐Ipi1), Nop53 (a recruitment factor for the exosome co‐factor Mtr4 helicase), Utp30 (a Cic1/Nsa3 homolog), and the endonuclease Las1 with its co‐factor, the polynucleotide kinase Grc3 (Gasse *et al*, 2015) (Fig 1B). We did not detect Rlp7 (Ribosomal‐like protein 7) in our preparation, consistent with the previous finding that Rlp7 does not exist in *C. thermophilum* but is replaced by the homologous Rpl7, which is the authentic ribosomal protein L7/uL30 (Lau *et al*, 2023). Arx1, which is another characteristic pre‐60S factor in yeast, was also absent from the Flag eluate, although it exists in *C. thermophilum*, but also the human homolog Ebp1 was not found on nucleoplasmic pre‐60S particles (Liang *et al*, 2020; Bhaskar *et al*, 2021).

**Figure 2:**
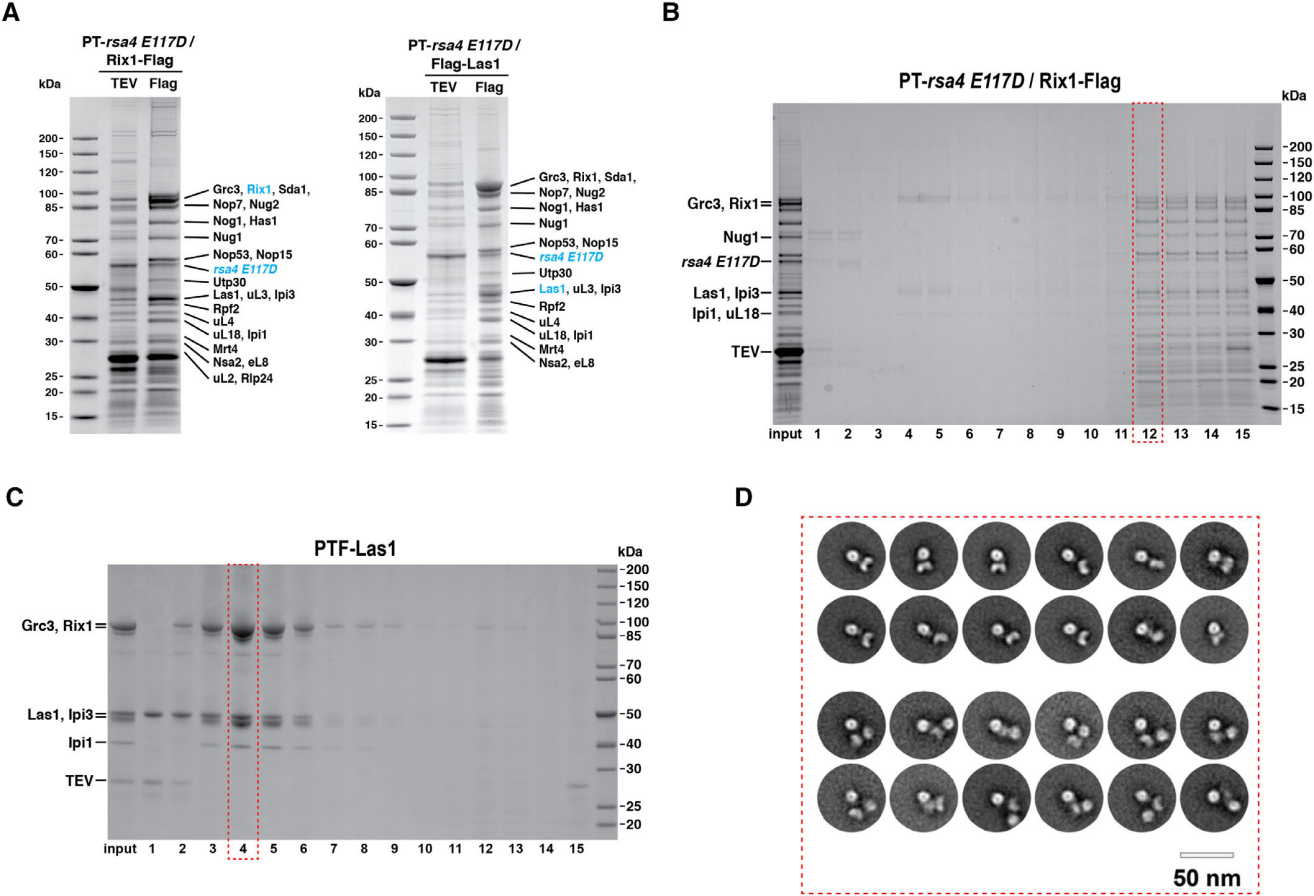
Affinity‐purification of the *C. thermophilum* pre‐60S particles enriched in the rixosome (Rix1‐Ipi3‐Ipi1‐Las1‐Grc3) Split‐tag affinity‐purification using PT‐Rsa4 E117D as first bait, followed by Rix1‐Flag (left) or Flag‐Las1 (right) as second bait enriching pre‐60S particles that contain the Rix1‐Ipi1‐Ipi3‐Las1‐Grc3 (rixosome) complex. Both the first TEV and second Flag eluate were analyzed by SDS–PAGE and Coomassie staining. Labeled bands were identified by mass spectrometry, with bait proteins labeled in blue. Both affinity‐purifications were performed at least twice with similar outcome.Sucrose gradient centrifugation (10–40%) of the final PT‐Rsa4 E117D Rix1‐Flag split‐tag eluate as shown in (A). The input and gradient fractions 1–15 were analyzed by SDS–PAGE and Coomassie staining. The labeled bands were excised from the gel and identified by mass spectrometry.Affinity‐purification of PTF‐Las1 from wild‐type *C. thermophilum* lysates (final eluate, Input), which was further fractionated by sucrose gradient centrifugation (10–40%). Input and gradient fractions 1–15 were finally analyzed by SDS–PAGE and Coomassie staining. Bands labeled on the left were identified by mass spectrometry.Negative‐stain electron microscopy of sucrose gradient fraction #4 (see C) from the final PTF‐Las1 Flag eluate containing the highly purified rixosome complex (see also C). Shown are typical 2D classes of the rixosome, consisting of the Rix1 subcomplex (hollow sphere) in association with the Las1‐Grc3 heterodimer (butterfly‐like). Scale bar, 50 nm. Source data are available online for this figure.

The Las1‐Grc3 and the Rix1 complex were previously described in *S. pombe* and humans as members of the “rixosome” (Shipkovenska *et al*, 2020; Zhou *et al*, 2022), but were also called RIXC in another study (Holla *et al*, 2020). As we could find these factors in the *ct* Rsa4 E117D Nop7‐Flag preparation as prominent bands (see Fig 1B), we sought to examine the rixosome‐carrying pre‐60S particles in more detail. Thus, we generated *ct* double transformants with *PT‐rsa4 E117D* as first and either *Rix1‐Flag* or *Flag‐Las1* as second affinity baits. As anticipated, the final PT‐Rsa4 E117D Rix1‐Flag and PT‐Rsa4 E117D Flag‐Las1 eluates isolated from these strains were strongly enriched in Rix1‐Ipi1‐Ipi3 and Las1‐Grc3 (Fig 2A). Such an enrichment of Las1‐Grc3 has not been observed in related yeast pre‐60S particles, although *sc* Las1 has been shown to endonucleolytically cleave ITS2 at site C_2_, followed by Grc3‐catalyzed phosphorylation of the freshly generated 5′‐OH end of the 26S pre‐rRNA. This latter reaction facilitates subsequent Rat1‐Rai1‐mediated 5′ > 3′ exonucleolytic trimming toward 25S rRNA (Gasse *et al*, 2015), before final 3′ > 5′ processing of the 7S rRNA by the RNA Mtr4/exosome and release of the foot factors Cic1/Nsa3, Nop15, Rlp7, and Nop7 can occur (Castle *et al*, 2013; Gasse *et al*, 2015; Fromm *et al*, 2017).

Finally, the *ct* Rsa4 E117D Rix1‐Flag eluate was analyzed by sucrose gradient centrifugation, which showed that the Rix1 complex and the Las1‐Grc3 co‐sedimented with the pre‐60S particles, suggesting a physical interaction of both sub‐modules with the pre‐60S subunit (Fig 2B).

### Purification and EM analysis of the purified rixosome from *Chaetomium thermophilum*

Based on the previous finding that isolated yeast Las1 is part of a Las1‐Grc3‐Rat1‐Rai1 complex (Gasse *et al*, 2015), we performed a similar purification from *C. thermophilum* lysate. However, wild‐type *ct* PTF‐Las1 still co‐enriched Las1‐Grc3 together with Rix1‐Ipi1‐Ipi3, but not Rat1‐Rai1, and all five subunits remained stably associated during extended sucrose gradient centrifugation (Fig 2C). Interestingly, the “rixosome” recently described in *S. pombe* and humans was suggested to have a dual role in ITS2 processing (Castle *et al*, 2010, 2012; Schillewaert *et al*, 2012) and heterochromatin spreading linked with epigenetic inheritance (Shipkovenska *et al*, 2020; Zhou *et al*, 2022). Thus, it is possible that the *ct* Las1‐Grc3‐Rix1‐Ipi3‐Ipi1 complex, for which we also use the synonym “rixosome”, performs a similar twofold function.

To gain insight into the structure of the *ct* rixosome, we started with negative‐stain electron microscopy of the purified *ct* Las1‐Grc3‐Rix1‐Ipi3‐Ipi1 complex (see Fig 2C, fraction #4). We often observed two characteristic sub‐structures in close proximity, a sphere‐like structure of 10 nm ± 1 nm and a butterfly‐like structure of 13 × 8 nm ± 1 nm but without a connecting element (Fig 2D, upper rows). In addition, we also found classes containing two ball‐like structures adjacent to one butterfly‐like base structure (Fig 2D, lower rows).

Since the Rix1‐Ipi3‐Ipi1 complex is known to exhibit a large hollow sphere with a diameter of ~9 nm in yeast (Barrio‐Garcia *et al*, 2016; Kater *et al*, 2020) and the reconstituted *ct* Las1‐Grc3 heterodimer a butterfly‐like structure of 13 nm × 7 nm (Pillon *et al*, 2017), we conclude that we can observe the entire rixosome by electron microscopy.

**Figure 3:**
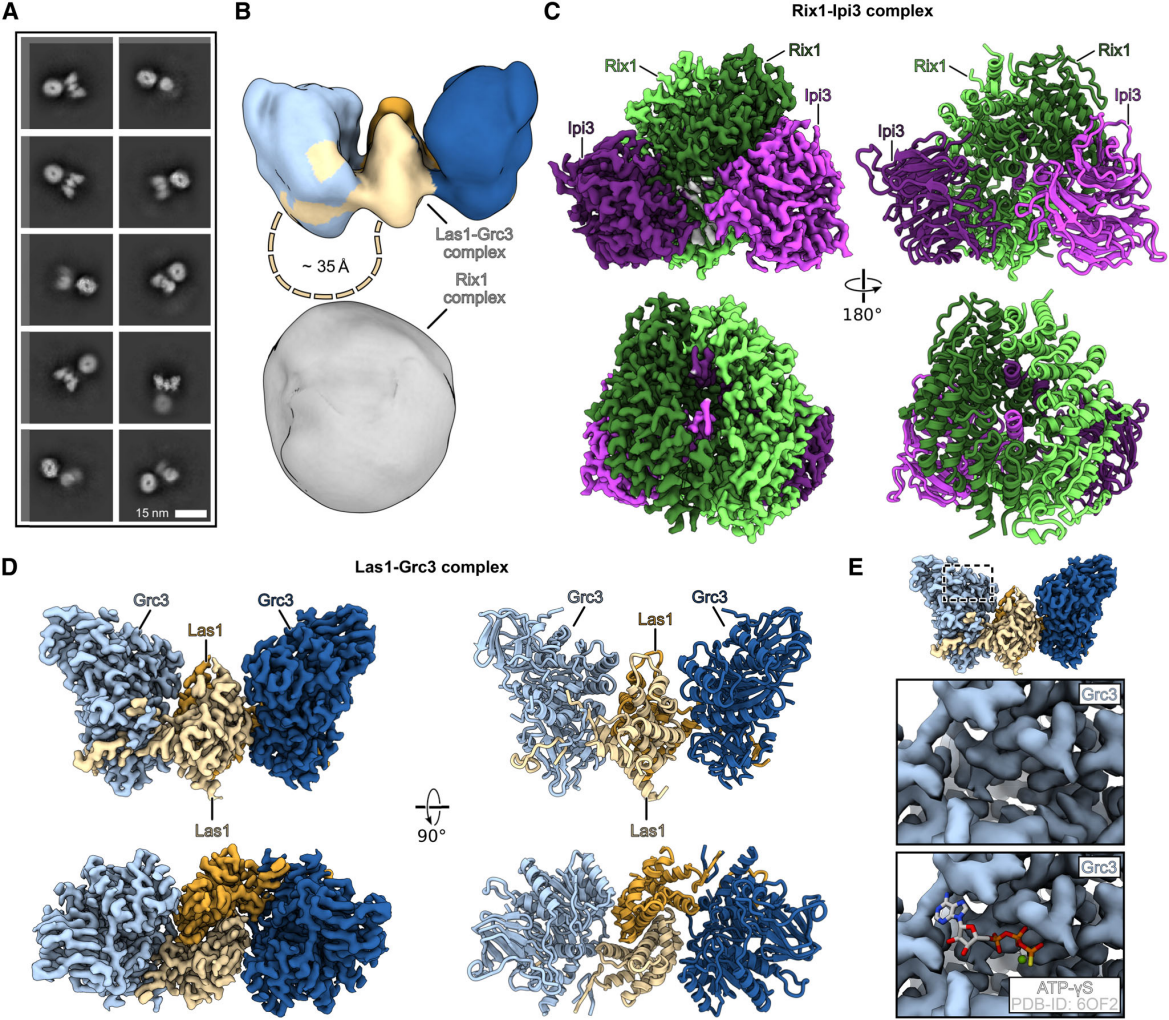
Cryo‐EM structure of the isolated rixosome from *C. thermophilum* A
Selected 2D classes of the rixosome complex.B
Low‐resolution cryo‐EM map of the rixosome. The surface is colored according to the Las1‐Grc3 complex shown in (D). The approximate distance between the Las1‐Grc3 complex and the globular Rix1 complex is indicated, and the unresolved Las1 CC domain is indicated as dashed line.C, D
Colored cryo‐EM maps (left) and molecular models (right) of the individually processed Rix1 complex (C) and the Las1‐Grc3 complex (D) shown in two orientations.E
The Las1‐Grc3 was found in the apo‐state. Close‐up of the nucleotide‐binding pocket of Grc3 (middle) and overlay with the ATP‐γS model of PDB‐ID 6OF2 (bottom).

Next, we performed cryo‐EM of the *ct* rixosome to unravel additional structural details (Appendix Fig S2 and Appendix Table S2). Once again, we observed the spherical density, which we attribute to the Rix1‐complex, and the butterfly‐shaped Las1‐Grc3 (Fig 3A and B), both arranged in such a way that the shorter edge of Las1‐Grc3 is preferentially oriented toward the Rix1 complex, with a distance of about 35 Å. While the cryo‐EM reconstruction of the complete rixosome could only be resolved at low resolution and did not reveal any apparent connecting density, both sub‐complexes could be resolved individually to resolutions of 2.9 Å (Rix1 complex) and 3.0 Å (Las1‐Grc3 complex) (Fig 3C and D). Accordingly, the Rix1 complex resembles essentially the structure observed on yeast pre‐60S particles, composed of two copies Rix1 and two copies Ipi3 (Kater *et al*, 2020), but without a rigidly bound Ipi1 subunit (Appendix Fig S2). This suggests a flexible connection of Ipi1 to the spherical Rix1‐Ipi3 core on the isolated rixosome, but on pre‐60S particles Ipi1 becomes fixed upon its binding to Sda1 (Fig 3C; see below). Characteristically, the Las1‐Grc3 assembly displayed a two‐fold symmetry as observed previously, with two copies of Las1 and Grc3 and without densities for nucleotides or RNA, thus representing the apo‐state (Fig 3E) (Pillon *et al*, 2019).

### Modeling of the linker between Rix1 complex and Las1‐Grc3

**Figure EV1:**
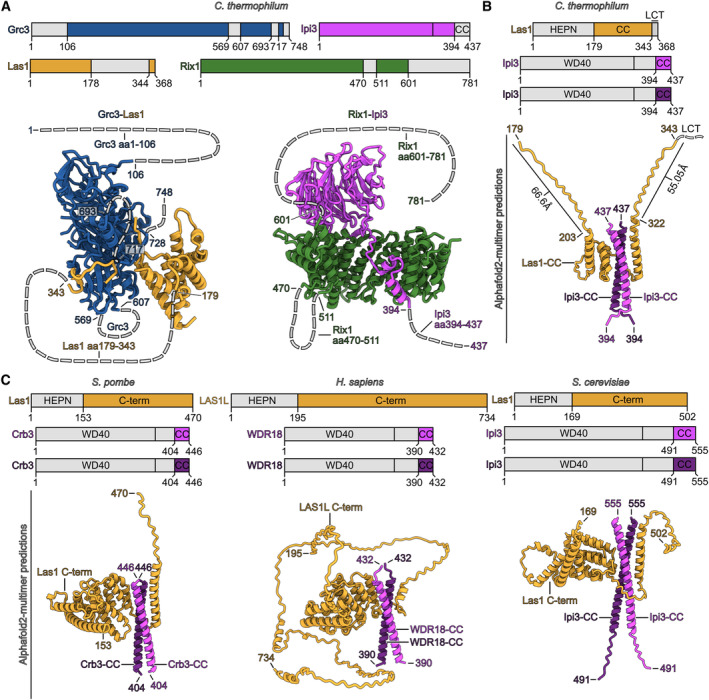
Alphafold predictions of the linker regions between Rix1 complex and Las1‐Grc3 complex within the rixosome Schematic representation and molecular models of Grc3, Las1, Ipi3, and Rix1 shown in different colors. Regions not included in the molecular models are indicated in gray.Alphafold2‐multimer model of the Las1 coiled‐coil domain (Las1‐CC, aa179‐343) in complex with a dimer of the C‐terminal coiled‐coil helix of Ipi3 (Ipi3‐CC, aa394‐437). The maximal length of the unstructured regions are indicated in Å.Alphafold2‐multimer predictions of the Las1 C‐term/Ipi3‐CC interactions from *S. pombe*, *H. sapiens*, and *S. cerevisiae*.

Since we did not observe additional densities on the rixosome, which could explain how the sub‐modules are physically connected, we wondered which sequences not detectable in our maps could make this link and probed this possibility using Alphafold 2 (AF2) multimer (Fig EV1A). Thus, we investigated whether the invisible sequences of the Las1 C‐terminal coiled‐coil (CC) domain, Las1 (aa179‐344), Grc3 N‐terminal domain (aa1‐107), Ipi3 (aa394‐437), Rix1 (aa601‐781), and the Ipi1 subunit could create such a putative connection. Strikingly, AF2 multimer predicted with high consistency an interaction between the Las1‐CC and an intertwined bundle of two CC α‐helices formed by the two C‐termini of Ipi3 (Fig EV1B). The predictions agree with the previous finding that the Ipi3 CC domain is essential for Ipi3 homo‐dimerization (Barrio‐Garcia *et al*, 2016). In contrast, AF2multimer did not predict reliable interactions, neither between Las1 and Rix1 C‐terminus (aa601‐781) nor between Las1 and Ipi1. The validity of the Las1–Ipi3 interaction was further strengthened by AF2 multimer, which consistently predicted highly similar interactions between other Las1 and Ipi3 homologs from *S. cerevisiae*, *S. pombe* and *H. sapiens*, suggesting a conserved mechanism for rixosome sub‐module linkage (Fig EV1C). Finally, the predicted interaction agrees well with the observed distance and orientation between the two sub‐complexes in the *ct* rixosome cryo‐EM structure, allowing us to further speculate how the flexible Las1‐Grc3 module as part of the rixosome could be located within the pre‐60S particle (see below).

### Cryo‐EM structures of *ct* pre‐60S particles before and after 5S RNP rotation

**Figure 4:**
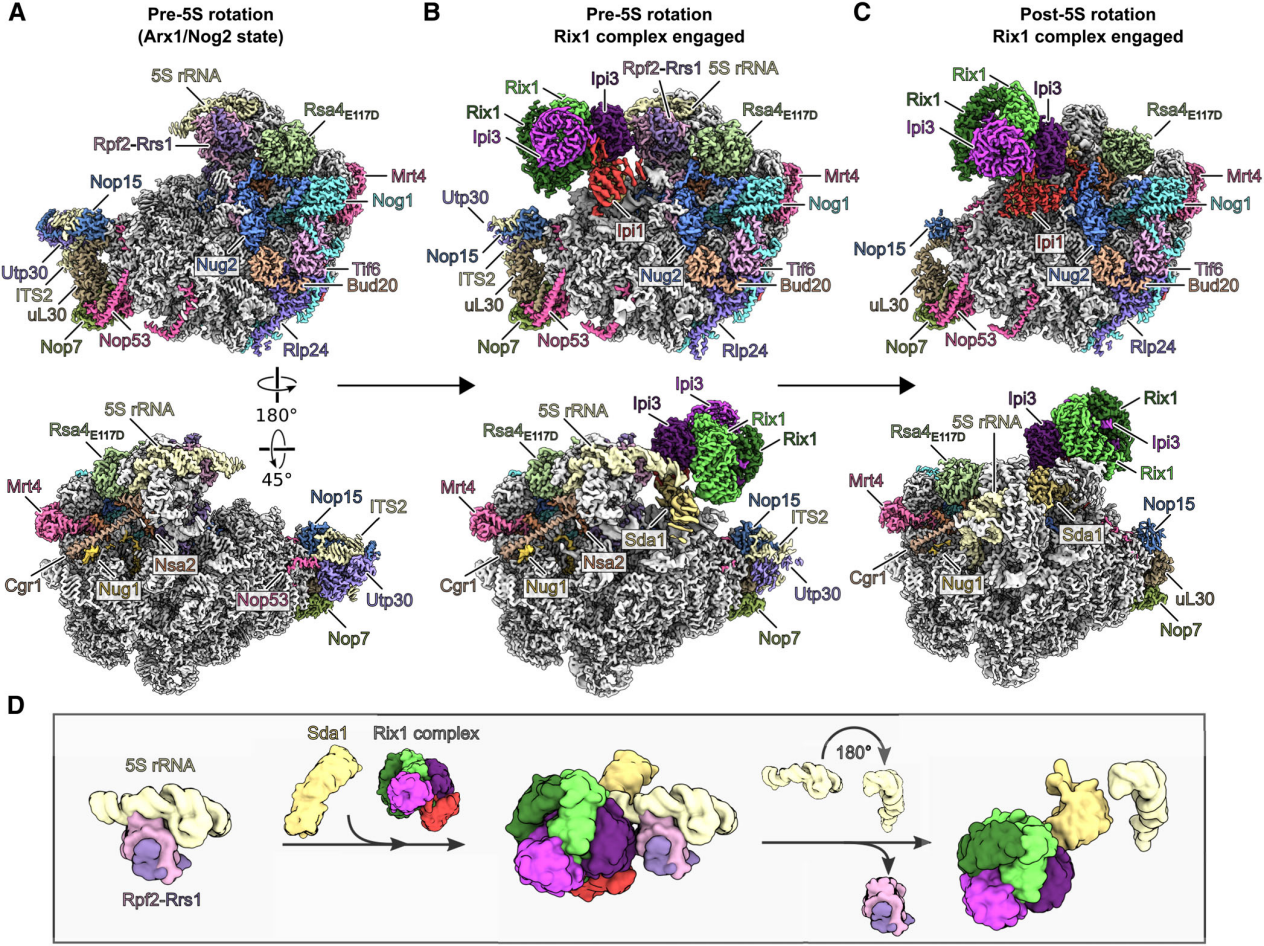
Cryo‐EM structure of pre‐60S particles before and after 5S rRNA rotation A–C
Cryo‐EM maps of three sequential pre‐60 states shown in two orientations. The maps are colored according to the respective molecular models and biogenesis factors, 5S rRNA and ITS2 are labeled. Ribosomal rRNA and ribosomal proteins are shown in gray.D
Model illustrating the molecular steps in the rotation of the 5S rRNP throughout the three states. The 5S rRNA, Rpf2‐Rrs1, Rix1 complex, and Sda1 are shown.

**Figure EV2:**
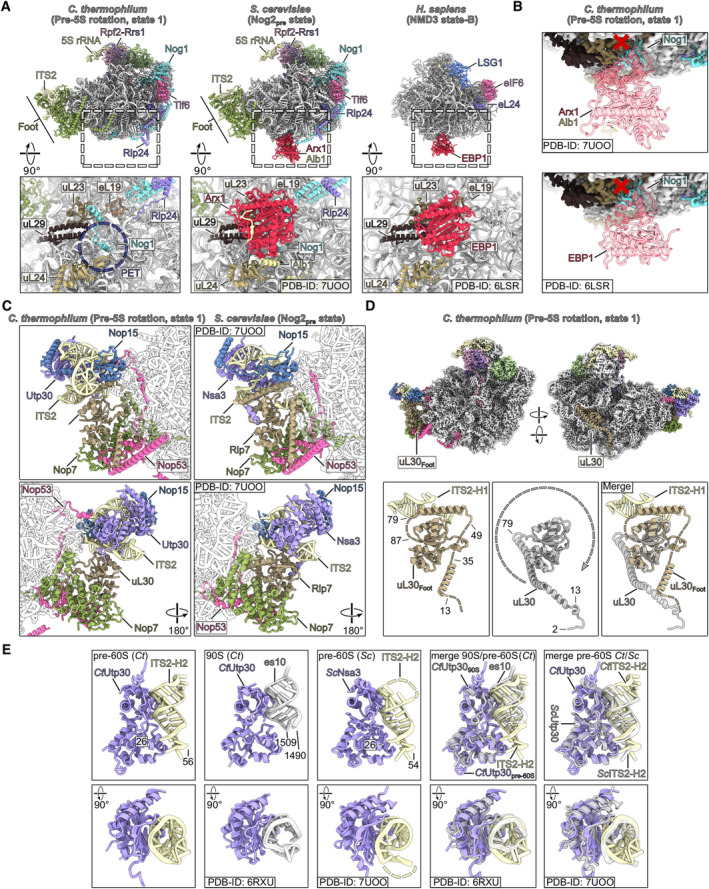
Structural comparison of Arx1 and foot Nucleoplasmic pre‐60S particle from *C. thermophilum* lack the polypeptide exit tunnel (PET) bound export factor Arx1. Comparison between nucleoplasmic pre‐60S states from *ct* (left, state 1) and yeast (middle, PDB‐ID: 7UOO) and cytoplasmic pre‐60S particles after release of Nog1 and Rlp24 from human bound to EBP1 (human Arx1 homolog, right, PDB‐ID: 6LSR). The upper panels show the molecular models of the different states, and the lower panels focus on the polypeptide exit tunnel.Superposition of Arx1‐Alb1 model from yeast and the Ebp1 model from human onto the pre‐5S rotation state from *C. thermophilum* (state 1). The Nog1 C‐terminus from *C. thermophilum* sterically clashes with the binding sites of Arx1 and EBP1 rationalizing the lack of *ct* Arx1 on the here described nucleoplasmic pre‐60S states.Comparison of the *ct* ITS2 containing Foot structure (state 1) and the equivalent yeast state (Nog2_pre_, PDB‐ID: 7UOO).Colored cryo‐EM map of the *ct* pre‐5S rotation state 1 in two orientations highlighting the two copies of uL30 within the particle (upper panels). Detailed view of the individual uL30 copies (lower panel, left and middle) and merge of the two copies (lower panel, right). The N‐terminus of uL30_Foot_ has to rotate in comparison to the uL30 copy within the pre‐60S core to adapt the binding mode of Rlp7 within the yeast Foot.Structural comparison of ct Utp30 bound to helix 2 (H2) of the ITS2 within the Foot structure, *ct* Utp30 as part of the *ct* 90S bound to es10 (PDB‐ID: 6RXU) and the yeast foot factor Nsa3 bound to the H2 of the ITS2 (PDB‐ID: 7UOO). The models are labeled and shown in two orientations and as merge comparing pre‐60S *ct* Utp30/ITS2‐H2 with 90S *ct* Utp30/es10 and pre‐60S *sc* Nsa3/ITS2‐H2, respectively.

To find out how the *ct* rixosome is structurally integrated into the various thermophile pre‐60S particles obtained by split‐tag affinity‐purification (see above), we analyzed the Flag eluates from PT‐rsa4 E117D/Nop7‐Flag, PT‐rsa4 E117D/Rix1‐Flag and PT‐rsa4 E117D/Las1‐Flag by single particle cryo‐EM (Appendix Figs S3–S7 and Appendix Table S2). After extensive classification, we obtained several distinct states facilitating reconstruction of three successive nucleoplasmic pre‐60S states that, in contrast to yeast but consistent with our biochemical data (see Figs 1 and 2), are devoid of the nuclear export factor Arx1 (Fig EV2A and B). Among these *ct* pre‐60S particles, the first one in the series resolved at 2.1 Å resolution exhibits the 5S RNP in the pre‐rotation stage, but still lacks the Rix1 complex (Fig 4A), hence resembling the yeast Arx1/Nog2 particles (Leidig *et al*, 2014; Wu *et al*, 2016). The following two *ct* pre‐60S intermediates carry the Rix1 complex, where one state shows the 5S RNP in the pre‐rotation (2.9 Å resolution) and the other one in the post‐rotation position (2.9 Å resolution) (Fig 4B and C). The latter state resembles the yeast Rix1‐Rea1 pre‐60S particle (Barrio‐Garcia *et al*, 2016; Kater *et al*, 2020), but without the Rea1 AAA‐ATPase consistent with our biochemical data (see Figs 1 and 2). During this transition, the pre‐60S factors Rpf2 and Rrs1, which decorate the 5S RNP prior to relocation, dissociate from the 5S RNP during the ~180° rotation before it finally locks in its mature‐like conformation on the subsequent pre‐60S intermediate (Fig 4D). Importantly, while the *ct* pre‐60S carrying the Rix1 complex and the 5S RNP in pre‐rotation state has not been observed in yeast yet, this intermediate gives insight into to the possible mechanism of 5S RNP rotation during 60S biogenesis (see below). Despite the strong and stable biochemical enrichment of Las1‐Grc3 in the *ct* pre‐60S preparations, no cryo‐EM density for these two factors could be assigned, consistent with a flexible connection between the Rix1 complex and Las1‐Grc3 heterodimer (see above).

**Figure 5:**
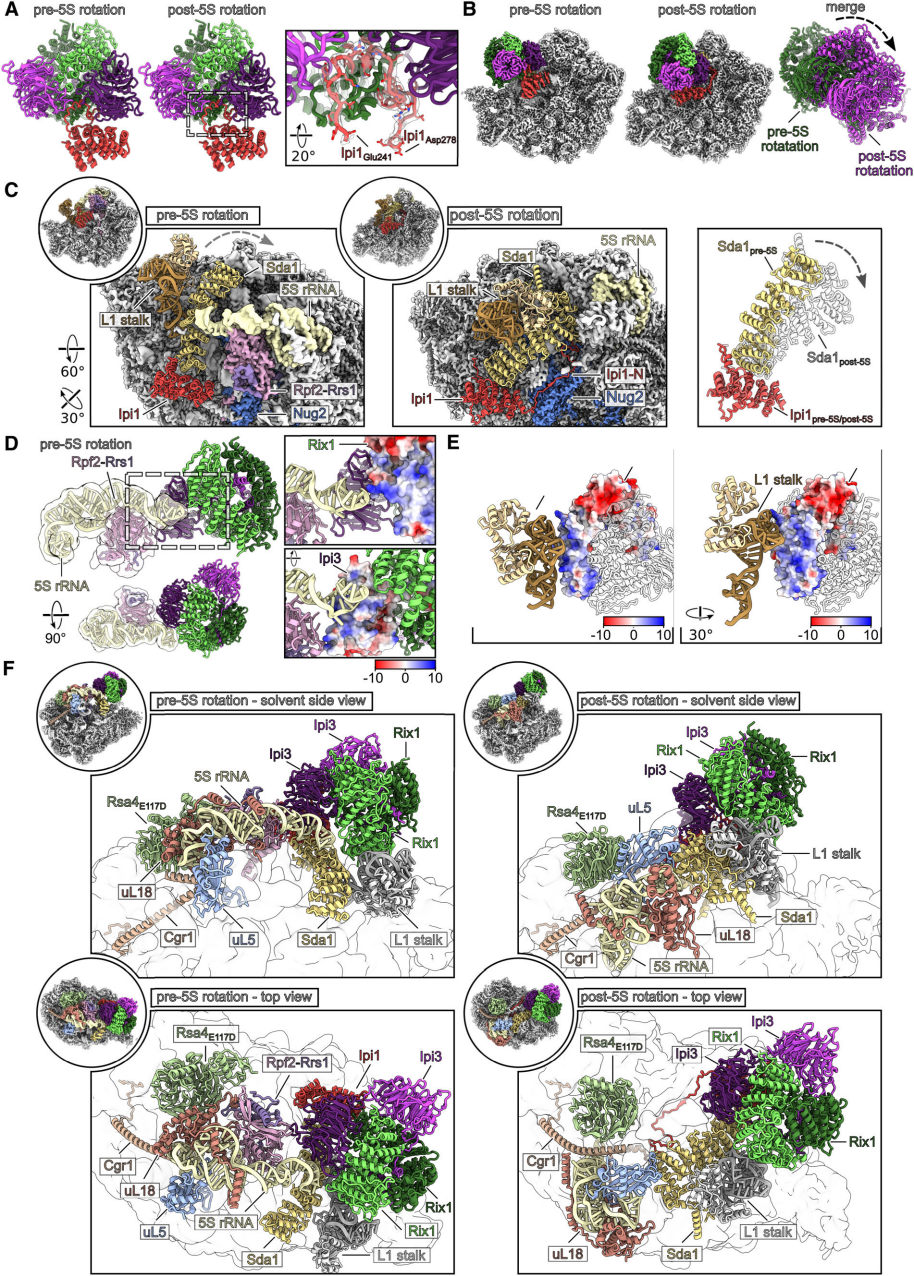
Rix1 containing pre‐60S particles before and after 5S rRNA rotation Molecular model of the Rix1 complex on the pre‐5S and post‐5S rotation particles. Close‐up showing the Ipi1 loop (aa241‐278) interacting with the Rix1‐Ipi3 sphere.Colored maps of the pre‐5S and post‐5S rotation particles highlighting the Rix1 complex and molecular models of the Rix1 complex from both states illustrating its movement.Sda1 and the L1 stalk rearrange during 5S rRNA rotation (left and middle panel). The pre‐60S moieties of both states are shown as gray density and 5S rRNA, Rpf2‐Rrs1, and Nog2 are colored. Sda1‐Ipi1 and the L1 stalk are shown as molecular model and the Rix1‐Ipi3 complex was omitted for clarity. Overlay of the Sda1‐Ipi1 complex from the pre‐5S and post‐5S rotation particles (right panel). The two Ipi1's were rigid body fitted to indicate the movement of Sda1 between both states.The Rix1 complex interacts with the 5S rRNA tip and Rpf2‐Rrs1 in the pre‐5S rRNA rotation state (left side). Models of 5S rRNA, Rpf2‐Rrs1, and Rix1‐Ipi3 and the Gaussian filtered segmented densities of 5S rRNA and Rpf2‐Rrs1 are shown in two orientations. One copy of each Ipi3 and Rix1 form a positively charged surface interacting with the 5S rRNA tip (right side). Magnifications show the surface views of Rix1 and Ipi3 colored according to the respective electrostatic potential.The positively charged N‐terminal half of one of the Rix1 copies is in contact with the L1 stalk rRNA in the pre‐5S and post‐5S rotation state. The surface colored according to the electrostatic potential for the interacting Rix1 copy and transparent models for the other Rix1 complex members are shown. The L1 stalk is shown as molecular model.Overview of the intrinsic 5S rRNP rotation network comparing the pre‐5S (right side) and post‐5S rotation state (left side). Factors involved in stabilization and rotation of the 5S RNP are shown as colored molecular models and the pre‐60S moieties as filtered transparent densities.

**Figure EV3:**
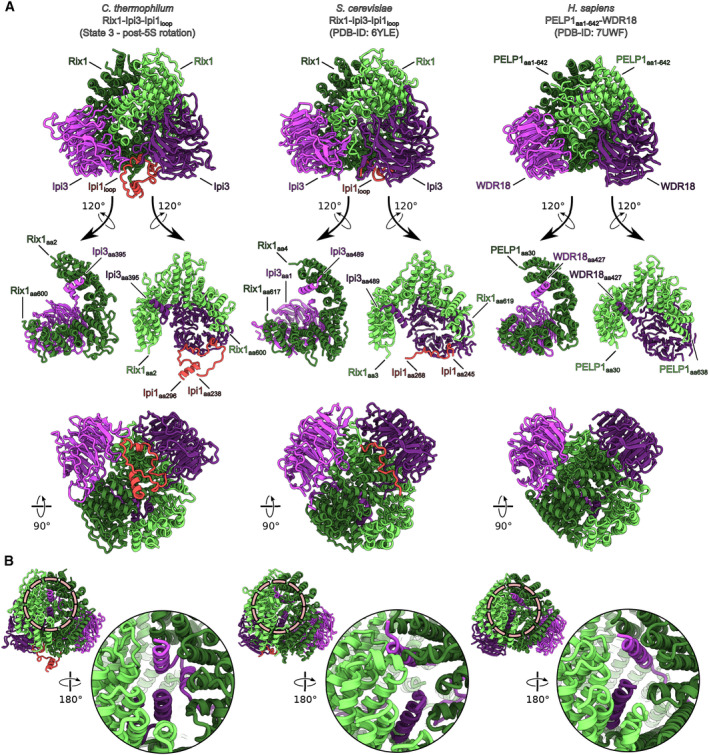
Comparison of the Rix1 complex structures from *C. thermophilum* and *S. cerevisiae* and the human PELP1‐WDR18 complex The Rix1 complex shows strong structural conservation between organisms. Comparison of the molecular models of the Rix1 complex from *C. thermophilum*, *S. cerevisiae*, and the PELP1‐WDR18 complex from *H. sapiens*.Detailed view of the C‐termini of Ipi3/WDR18 for each species. The termini are similarly positioned and able to protrude from the Rix1 complex, potentially form coiled‐coils and interact with Las1 (see Fig EV1).

The Rix1 complex from *C. thermophilum* exhibits a highly similar structural organization when compared to the structures described in *S. cerevisiae* and human (Fig EV3; Pillon *et al*, 2019; Kater *et al*, 2020). The hollow sphere‐like structure is formed by two copies each of Rix1 and Ipi3. Similar to yeast the single copy of Ipi1 is bound to this structure through a loop insertion within its alpha‐helical domain. The arrangement of the Rix1‐Ipi3‐Ipi1 complex is essentially identical in both states (Fig 5A). Nevertheless, we observe a large movement between pre‐ and post‐rotation states (Fig 5B). This movement is facilitated by Sda1 via its N‐terminal region interacting in both states with Ipi1 which serves as an anchoring pivot. In the pre‐5S rotation state Sda1 is flexible bound to the intersubunit space (ISS) of the particle between the L1 stalk and the tip of the 5S rRNA (Fig 5C). Here, we observed contacts between the Rix1 complex and Rpf2‐Rrs1, as well as with the tip of the unrotated 5S rRNA, to which one of each Ipi3 and Rix1 copies form a positive interaction patch (Fig 5D). However, Rpf2‐Rrs1 as well as the connecting rRNA helices H80‐88 of the immature CP show no structural differences between the pre‐5S rotation states with and without engaged Sda1 and Rix1 complex. Another prominent interaction side in both states is mediated by the positively charged N‐terminal part of one of the Rix1 subunits and the L1 stalk rRNA (Fig 5E). For the 5S RNP rotation the entire Rix1 complex, the adjacent L1 stalk, and Sda1 move together toward the central protuberance that eventually brings the Sda1 C‐terminal domain to its final position to bind to the L1 protein (Fig 5C). Notably, before the 5S RNP rotation the Rix1 complex is already in contact with the tip of the pre‐rotated 5S rRNA. The concerted movement then generates a steric clash between the L1 stalk and the tip of the 5S RNA, and thus initiates the overall 5S RNP relocation (Fig 5F).

### Role of the rixosome in coordinating 5S RNP rotation with ITS2 processing

As Las1 and Gcr3 are biochemically incorporated in our nucleoplasmic pre‐60S particles but remained invisible in cryo‐EM, we wondered where this flexible rixosome submodule could be located. Thus, we superimposed the cryo‐EM structure of the free rixosome with the pre‐60S structure by merging the ball‐like volume of the free rixosome with the Rix1 complex structure bound to the pre‐rotation state. As ITS2 and the herein C_2_ cleavage site are embedded into the pre‐60S foot structure, we first had a closer look into this hallmark structure within our *ct* pre‐60S particles. As previously found for nucleolar *ct* pre‐60S particles (Lau *et al*, 2023), the Rsa4 E117D derived nascent 60S also exhibits this typical foot structure, containing the Rpl7/uL30 instead of the classical but homologous yeast foot factor Rlp7 (see above), and the 90S factor Utp30 instead of the homologous yeast foot factor Cic1/Nsa3 (Fig EV2C and D). Like Cic1/Nsa3 in yeast, *ct*Utp30 interacts with the highly conserved assembly factor *ct* Nop15, and in addition has direct contact to ITS2 RNA helices H1 and H2 (Fig EV2C and E). However, the following ITS2 H3 is barely visible in our nucleoplasmic *ct* pre‐60S, suggesting that the first steps in ITS2 processing, that is, endo‐nucleolytic C_2_ cleavage and subsequent 5′‐end RNA modification by Las1‐Grc3, as well as 5′ trimming of the generated 27S rRNA toward mature 25S rRNA by the Rat1/Rai1, might have already occurred.

**Figure 6:**
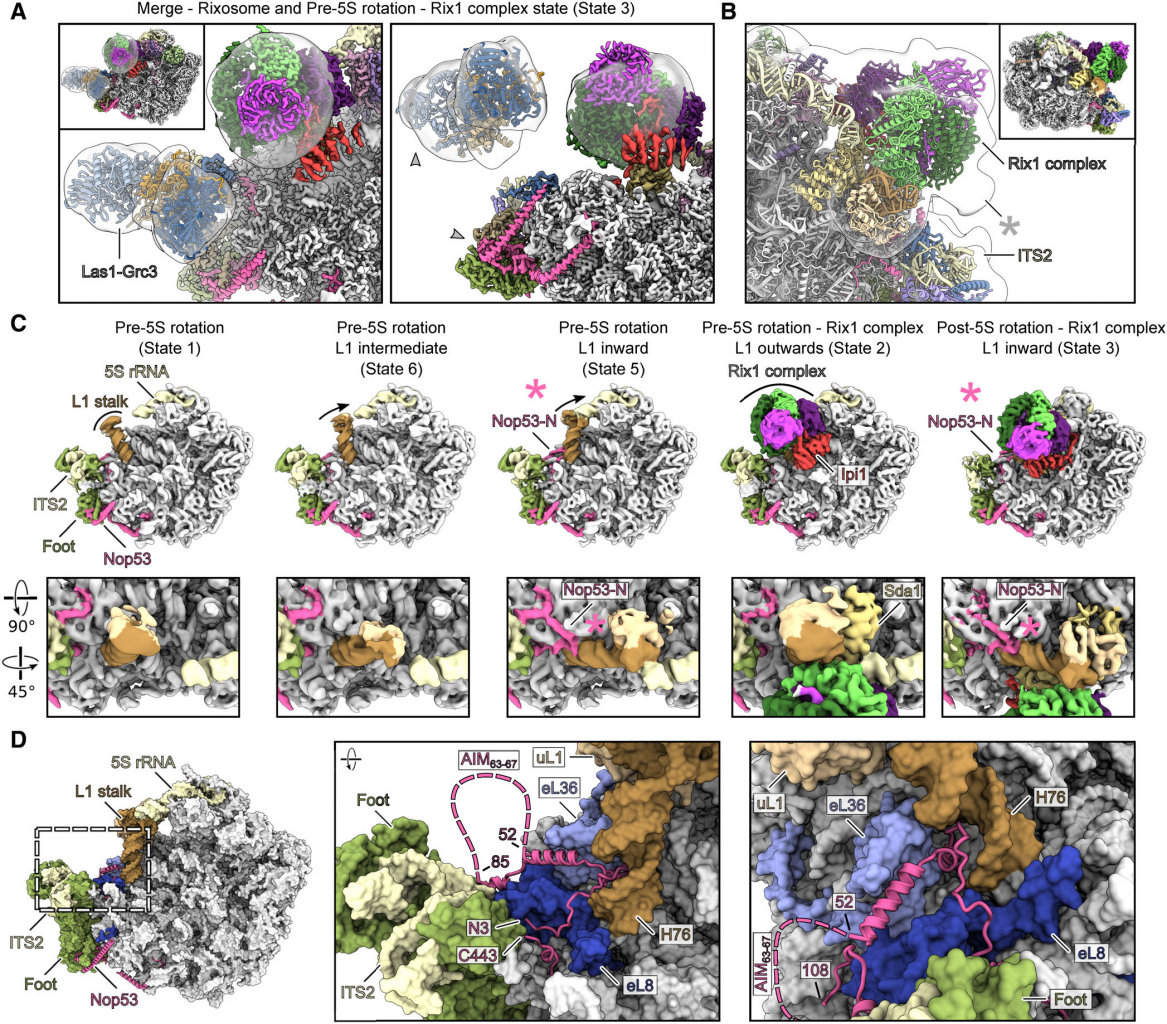
Orientation of the rixosome and Nop53 N‐terminus carrying the AIM motif during 5S RNP rotation Superposition of the rixosome density onto the colored map of the pre‐5S rotation state with an engaged Rix1 complex shown in two orientations. The globular ball‐like part of the rixosome map was superimposed with the Rix1 complex density and the butterfly‐like Las1‐Grc3 density faces toward the ITS2‐foot structure. The colored model of the Las1‐Grc3 complex was rigid body fitted.Close‐up of the Rix1 complex area within the pre‐5S rotation state, showing the molecular model and the corresponding density low pass filtered to 25 Å. An asterisk denotes an additional density protruding from the Rix1 complex at the location where the C‐terminal coiled‐coil helices are presumed to emerge.Low pass filtered and colored maps of different pre‐60S states. The movement of the L1 stalk is indicated. The N‐terminus of Nop53 (modeled from aa3‐52) binds the pre‐60S particle when the L1 stalk is in an inward position either contacting the 5S rRNA tip in the pre‐5S rotation state (middle) or in the post‐5S rotation state (right), marked with an asterisk.Colored surface model of the pre‐60S state with the L1 stalk in the inward position. The molecular model on Nop53 is shown. Close‐up views highlight the N‐terminus of Nop53 and its interaction partners. The unresolved region between aa52 and aa85 containing the AIM motif is indicated as dashed line. The N‐ and C‐termini of the Nop53 model are labeled as N3 and C433, respectively.

When we superimpose the free rixosome density into the *ct* pre‐60S particle with 5S RNP in the pre‐rotation state, we notice that the Las1‐Gcr3 submodule becomes well positioned between the Rix1 complex and the foot structure, so that Las1 should be able to reach its C_2_ cleavage site within ITS2 that also flexibly protrudes from the pre‐60S foot (Fig 6A; see also Lau *et al*, 2023). In agreement with this superimposition depicting Las1‐Grc3 adjacent to ITS2, we observe a density protruding from the Rix1 ball after filtering the Rix1 complex to 25 Å resolution (Fig 6B). This density might be the beginning of the flexible linker connecting the ball‐like Rix1 complex with Las1‐Grc3 and composed of the Ipi3‐CC and the two attached Las1‐CC motifs (Figs 6B and EV1B). Thus, the relative neighborhood of the Las1‐Grc3 subcomplex involved in the ITS2 processing and the Rix1 complex with a facilitating role in 5S RNP rotation potentially allows for the coordination of both processes (see Discussion).

### Rixosome‐containing pre‐60S particles exhibit an exposed Nop53 AIM motif for exosome recruitment

**Figure EV4:**
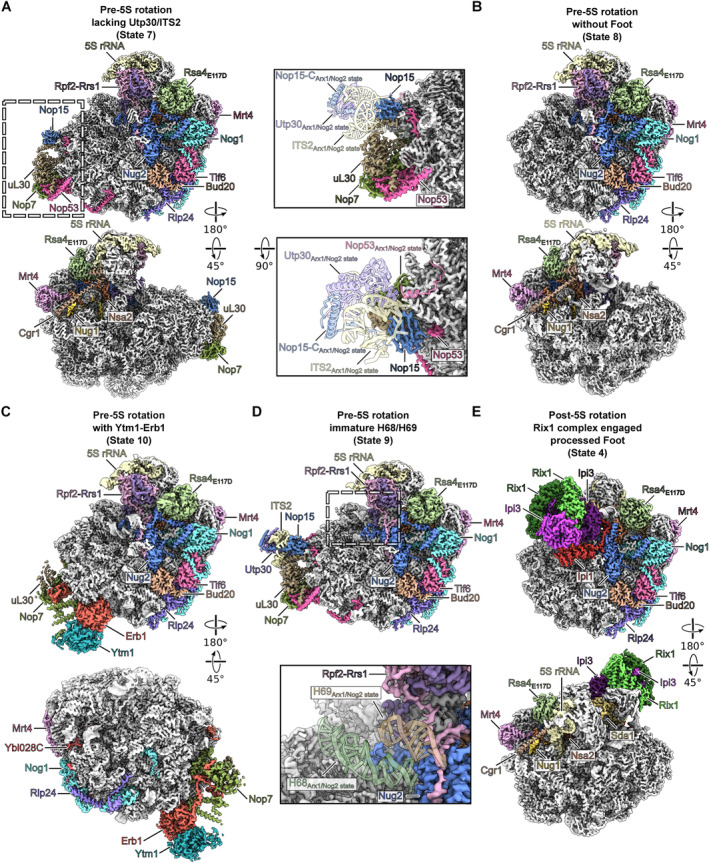
Additional states A–E
Colored cryo‐EM maps of additional pre‐60S states identified in the study. (A) Close‐ups showing the colored density map of the partly processed foot structure of the pre‐5S rotation‐lacking Utp30/ITS2 state. Superimposed models of the pre‐5S rotation state (Arx1/Nog2 state) indicating the missing parts: Utp30, ITS2, the C‐terminal part of Nop15 (aa277‐335), and Nop53 (aa85‐107). (D) Close‐up of the immature H68/H69 area with superimposed transparent model of H68 and H69 of the pre‐5S rotation state (Arx1/Nog2 state).

Notably, ITS2 and Utp30 (Cic1/Nsa3 in yeast) were barely detectable in the foot structure of the pre‐60S particles that already have reached the 5S RNP post‐rotation stage, although other foot factors such as uL30 and Nop7 as well as Nop53 (see below), remained clearly visible (Figs 4C and 6C). Moreover, even a few 5S RNP post‐rotation stages could be found, in which the ITS2 foot structure was completely absent (Fig EV4E, Appendix Fig S5D). Thus, it is conceivable that 5S RNP rotation, which is linked to the above described Rix1 complex movements, may play a role in allowing the final ITS2 processing reactions catalyzed by the nuclear exosome (see Discussion).

**Figure EV5:**
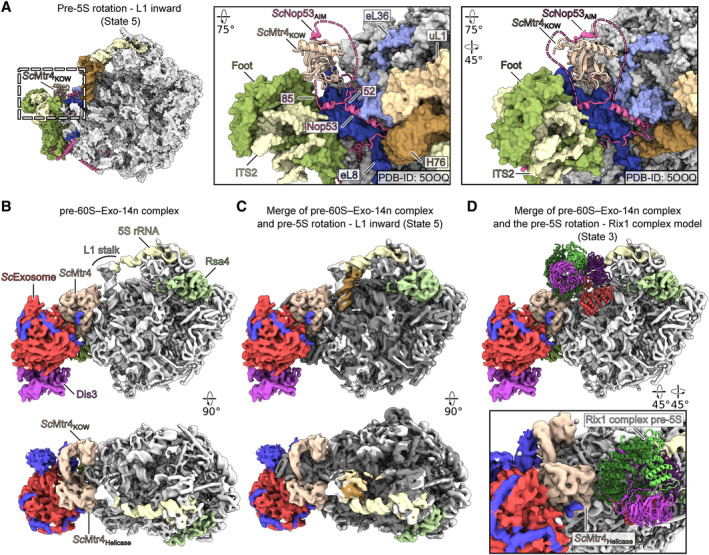
Structural comparison with the pre‐60S‐Exo‐14n complex Colored surface representation of the pre‐5S rotation state with L1 in the inward position (State 5). Nop53 is shown as model. The superimposed KOW domain together with the Nop53 AIM motif from *S. cerevisiae* is shown (PDB‐ID: 5OOQ). Only aa666‐818 Mtr4‐KOW and aa59‐70 Nop53‐AIM are shown for clarity. The model was rigid body fitted into the merged pre‐60S‐Exo‐14n structure. Dashed lines indicate the flexible, unmodeled parts of Nop53 wrapping around the Mtr4‐KOW domain.Filtered composite map of the pre‐60S‐Exo‐14n complex from *S. cerevisiae* arrested at the 5.8S+30 pre‐rRNA step (EMDB‐IDs: 4301 and 4302, PDB‐IDs: 6FT6 and 6FSZ).The filtered pre‐60S‐Exo‐14n complex composite map superimposed with the *C. thermophilum* pre‐5S rotation–L1 inward state (State 5) shown in dark gray and the L1 stalk, 5S rRNA, and Rsa4 in brown, yellow, and green, respectively.Overview (upper panel) and detailed view (lower panel) of the merge between the pre‐60S‐Exo‐14n density and the pre‐5S rotation state with engaged Rix1 complex (State 3). Only the Rix1 complex model is shown for clarity. Mtr4 and the Rix1 complex do not sterically interfere with each other.

Since the recruitment of the Mtr4‐exosome complex to nucleoplasmic pre‐60S particles is dependent on an AIM motif present in the N‐terminal extension of Nop53 (Thoms *et al*, 2015), we analyzed the possible local exposure of this exosome binding motif (aa63‐67) in the different nucleoplasmic *ct* pre‐60S particles. While the Nop53 C‐domain is always seen embedded into the foot structure of all three nucleoplasmic *ct* pre‐60S states, its unstructured N‐terminal extension becomes only visible when inserted into the base of the L1 stalk at helix H76 of the 25S rRNA. This fixation can only occur when the L1 stalk is twisted inwards (i.e., post‐rotation states with bound Rix1 complex; Fig 6C). Although we did not see the density of the AIM motif itself, the positioning of the adjacent upstream and downstream Nop53 N‐terminal sequences defines rather precisely the location of the AIM motif, directly neighboring to the ITS2 (Fig 6D). Consequently, upon subsequent Mtr4‐exosome recruitment by the Nop53 AIM motif, the exosome may be able to degrade the ITS2 toward the 3′ end of the 5.8S rRNA (Fig EV5A). This model agrees well with previous observations of a reconstituted *S. cerevisiae* pre‐60S particle, in which the Mtr4‐exosome was bound to the foot structure arrested at the 5.8S+30 processing step, again with the L1 stalk in an inward position (Fig EV5B and C) (Schuller *et al*, 2018). Superposition of the Rix1 complex onto the exosome bound to the pre‐60S indicates that both can co‐exist at the same particle (Fig EV5D). Thus, our proposed mechanism allows to couple Rix1 complex‐driven 5S RNP rotation with final ITS2 processing through the timely recruited Mtr4‐exosome RNA degradation machinery.

## Discussion

The findings of our study allowed us to derive at a better understanding of how the 5S RNP, which is first embedded into nucleolar pre‐60S particles in a premature conformation, can reach its mature position later in the nucleoplasm by a 180° rotation during a cascade of distinct remodeling steps. Moreover, this elaborate relocation can be potentially coupled with another important and complex pre‐rRNA processing step, namely the removal of the ITS2, an internal transcribed spacer sequence between 5.8S and 25S/28S rRNA occurring in all eukaryotic organisms. For ITS2 elimination, the Las1‐Grc3 complex plays a key role, which is best studied in yeast. Here, the 27S pre‐rRNA (5.8S‐ITS2‐25S) is first endonucleolytically cleaved at site C_2_ within ITS2 that generates the 7S and 26S pre‐rRNA forms. Next, Grc3 phosphorylates the 5′ end of the 26S rRNA that allows subsequent 5′ > 3′ processing toward the 25S rRNA by the Rat1‐Rai1 exonuclease complex, whereas the other half, the 7S pre‐rRNA, is 3′ > 5′ trimmed toward the 5.8S rRNA by the nuclear exosome.

Our model, which plausibly explains a possible coordination of these two processes at a molecular level, is based on the evaluation of different cryo‐EM structures obtained from a series of nucleoplasmic pre‐ribosomal particles isolated from our thermophile. Accordingly, the Rix1 complex of the rixosome could function like a central hub to trigger the 5S RNP rotation, and at the same time can position the other Las1‐Grc3 submodule close to the pre‐60S foot structure for ITS2 processing. Key for these two reactions is the unique structure of the rixosome, composed of the (Rix1)_2_‐(Ipi3)_2_‐Ipi1 complex and the attached but flexible Las1‐Grc3 heterodimer. Our data further demonstrated that the Rea1 AAA‐ATPase appears to be not needed for the 5S RNP relocation. This suggests that Rea1 has a role in final proofreading of these re‐arrangement steps on the pre‐60S. Thus, the binding site for the Rea1 ATPase on the pre‐60S may be only fully formed after 5S RNP rotation, and Rea1 recruitment to this pre‐60S might further stabilize the post‐rotation state (Kater *et al*, 2020). We thus provide a molecular mechanism for one of the most central and coupled remodeling events during 60S biogenesis.

However, the two events, 5S RNP rotation and ITS2 processing, are not strictly coupled. On one hand, maturation of the pre‐60S can proceed even in the absence of ITS2 processing as observed when using for example a nuclease‐dead Las1 mutant. Here, the pre‐60S were exported to the cytoplasm and entered the active translation cycle despite the presence of the foot structure (Sarkar *et al*, 2017). On the other hand, we observed partial or complete removal of the foot structure in particles prior to 5S rRNA rotation and in the absence of a tightly bound rixosome (Fig EV4) indicating that C_2_ cleavage and trimming can occur to some extent even before the rixosome could interact with the pre‐60S and trigger 5S RNP rotation. This could mean that there is no strict sequential order of these events, but that also branching and parallel maturation pathways are possible in principle. This speculation is corroborated by our identification of a pre‐60S particle class, in which the nucleolar Erb1‐Ytm1 complex was still bound while the ITS2 was already processed. While we cannot interpret the significance of this state at present, it should be mentioned that it was only observed with the Rsa4 E117D‐Nop7 split purification.

The unraveling of the Rix1 complex structure when physically bound to the Las1‐Grc3 heterodimer allows suggesting an analogous model for rixosome's second role in gene silencing and epistatic inheritance. Accordingly, the rixosome could be recruited to specific sites on heterochromatin to initiate the processing and degradation of heterochromatic RNAs. Since the Rix1 complex can associate with both nucleic acid (e.g., L1 stalk rRNA in our case) and protein factors (e.g., Sda1 in our case), we assume similar multiple contacts on chromatin can take place that guide the Rix1 complex to the marked sites on heterochromatin or silenced gene loci, where the adjacent Las1‐Grc3 module could trigger RNA cleavage and 5′ end phosphorylation, followed by final 5′ > 3′ degradation (via Rat1/Xrn2/Dhp1) and 3′ > 5′ degradation by the nuclear exosome.

Our findings are in good agreement with two recently published studies performed in the human system (Vanden Broeck & Klinge, 2023; Zhang *et al*, 2023). The study by Vanden Broeck and Klinge (2023) also identifies parts of the conserved NOP53 N‐terminus in a state where the L1 stalk is in its bent conformation interacting with the human RIX1 complex to facilitate exosome recruitment. Moreover, in the presence of the RIX1 complex an interaction of the very C‐terminus of LAS1L (Las1 homolog) and TEX10 (Ipi1 homolog) was observed, however, which appears to be specific to higher eukaryotes. Also these data support the notion of a conserved mechanism for coupling ITS2 processing with 5S RNP rotation. However, in both studies, different pre‐60S states with and without processed ITS2/Foot have been observed before as well as after 5S RNP rotation. These findings are in agreement with our interpretation of a loose coupling and the potential of parallel pathways.

The lack of visualization of the Las1‐Grc3 complex as part of the rixosome in our cryo‐EM structures of pre‐60S intermediates does not diminish our conclusions on how it mechanistically participates in coordinating distinct steps during pre‐60S maturation, but future work should aim to also visualize the structure of this conserved submodule on its pre‐60S target. The Las1‐Grc3 has a high degree of flexibility when attached to the Rix1 complex, but this is probably necessary to fulfill its specific function on the pre‐60S particles, reaching the C_2_ cleavage site for endonucleolytic cleavage and RNA 5′‐end phosphorylation, which is also in the highly flexible and exposed part of ITS2. On the other hand, this intrinsic mobility is probably key for the other function of the rixosome in gene silencing and heterochromatin formation. Thus, the usage of an artificial positioning sequences or reducing the linker length between the two submodules of the rixosome could help not only to further prove this mechanism but also to obtain stable structures amenable for cryo‐EM analysis.

## Materials and Methods

### Bacterial strains

For plasmid construction, the *E. coli* Dh5α (Thermo Fisher Scientific) strain was used.

### Chaetomium thermophilum strains

All *C. thermophilum* strains used in this study are derived from the DSM 1495 wild‐type strain (DSMZ, Braunschweig, Germany) and are listed in Appendix Table S1. Genetic manipulations of *C. thermophilum* strains expressing epitope‐tagged genes were generated as described previously (Kellner *et al*, 2016; Cheng *et al*, 2019).

The *NOP7‐Flag* construct was first transformed using the *erg1* selectable marker, followed by *PT*‐*rsa4 E117D* under the actin promoter with selection based on a heat‐adapted hygromycin resistance marker (Cannio *et al*, 2001; Cheng *et al*, 2019).

### Isolation of genomic DNA

Isolation of genomic DNA from *C. thermophilum* for whole genome sequencing was performed by two rounds of phenol‐chloroform extraction (Al‐Samarrai & Schmid, 2000).

### Tandem affinity purification of pre‐ribosomal particles and rixosome

For isolation of pre‐ribosomal particles by tandem affinity purification from *C. thermophilum*, mycelium was harvested after incubation for 20 h at 50°C in a rotary shaker, washed with water, dried under vacuum and subsequently frozen in liquid nitrogen. Cells were mechanically disrupted using a cryogenic grinding mill (Retsch MM400) in lysis buffer (60 mM Tris– HCl, pH 8.0, 40 mM KCl, 50 mM NaCl, 2 mM MgCl2, 5% glycerol, 1 mM DTT, 0.1% NP‐40, EDTA‐free protease inhibitors [SIGMA‐FAST], 0.013 U/l RiboLock RNase Inhibitors [Thermo Scientific]). The whole cell lysate was cleared by centrifugation for 10 min at 4,600 *g*, 4°C followed by a second round of centrifugation for 20 min at 35,000 *g*, 4°C. Supernatant was transferred to immune‐globulin G Sepharose 6 Fast Flow beads (GE Healthcare) and incubated for 12 h at 4°C. Bound proteins were washed with 15 ml wash buffer (60 mM Tris–HCl, pH 8.0, 40 mM KCl, 15 mM NaCl, 2 mM MgCl2, 5% glycerol, 1 mM DTT, 0.01% NP‐40), followed by TEV cleavage for 2 h at 16°C (60 mM Tris– HCl, pH 8.0, 40 mM KCl, 15 mM NaCl, 2 mM MgCl_2_, 5% glycerol, 1 mM DTT, 0.01% NP‐40, 1 U/l RiboLock RNase Inhibitors [Thermo Scientific]). After incubation the TEV eluate was loaded onto Flag‐agarose beads (Anti‐Flag M2 Affinity Gel, Sigma–Aldrich) and incubated for 10 h at 4°C. Flag‐beads were washed with 10 ml wash buffer and eluted with buffer containing the Flag peptide. The elution buffer for cryo‐EM analysis contained 60 mM Tris–HCl (pH 8.0), 50 mM NaCl, 5 mM MgCl_2_, 2% glycerol, 0.01% NP‐40, and 1 mM DTT.

### Sucrose gradient centrifugation of pre‐60S–rixosome particles and free rixosome

Flag‐eluates obtained from tandem affinity purifications were directly transferred onto a linear 10–40% (w/v) sucrose gradient (60 mM Tris–HCl (pH 8.0), 50 mM NaCl, 2 mM MgCl_2_, 0.003% NP‐40, and 1 mM DTT), and centrifuged for 16 h at 129,300 *g* at 4°C. After centrifugation the sucrose gradient was fractioned into 15 fractions, and each fraction was either precipitated with 10% trichloroacetic acid (TCA) or analyzed by negative‐stain EM. TCA‐precipitated proteins were resuspended in sample buffer and analyzed by SDS–PAGE followed by staining with colloidal Coomassie (Roti Blue, Roth).

### Mass spectrometry

Prominent bands labeled in the Coomassie‐stained gels were excised and identified by mass spectrometry (MALDI‐TOF).

### Negative‐stain electron microscopy and image analysis

Negative staining, data collection, and processing were performed as described previously (Gasse *et al*, 2015). In brief, the samples were generated as follows: 5 μl of the fractions were applied to a glow‐discharged grid covered with an approximately 6–8‐nm‐thick layer of continuous carbon. After incubation for 10 s, the sample was blotted on a Whatman filter paper 50 (1450‐070) and washed with three drops of water. Samples on grids were stained with 3% aqueous uranyl acetate. Images were acquired on a Thermo Fisher Talos L120C electron microscope equipped with a Ceta 16 M camera, operated at 120 kV. The micrographs were acquired at 45,000 × magnification using EPU software, resulting in 3.28 Å per pixel respectively. For 2D classification, 7,363 particles were selected using the boxer in EMAN2 (Tang *et al*, 2007). Image processing was carried out using the IMAGIC‐4D package (van Heel *et al*, 1996). Particles were band‐pass filtered, normalized in their gray value distribution and mass centered. Two‐dimensional alignment, classification, and iterative refinement of class averages were performed as previously described (Liu & Wang, 2011).

### Cryo‐electron microscopy and image processing

Samples obtained from affinity purifications (see Figs 1 and 2) were applied to TEM grids and plunge frozen in liquid ethane using a Vitrobot Mark IV (FEI). The PT‐Rsa4 E117D Nop7‐Flag, PT‐Rsa4 E117D Rix1‐Flag, and PT‐Rsa4 E117D Flag‐Las1 pre‐60S samples (3.5 μl) were applied to carbon‐coated R3/3 holey‐carbon support copper grids (Quantifoil) with 45‐s wait and 3‐s blot time at 4°C and 100% humidity. The PTF‐Las1 sample (3.5 μl) was applied to R1.2/1.3 holey gold grids (Quantifoil) with 3‐s blot time at 4°C and 100% humidity. For all datasets, movies were recorded on a Titan Krios (FEI) equipped with a K2 summit (Gatan) direct electron detector at 300 kV with a nominal pixel size of 1.045 Å. Movies were dose‐weighted, summed, and motion corrected using MotionCor2 (Zheng *et al*, 2017). For the summed micrographs CTF (contrast transfer function) parameters were estimated with CTFFIND4 (Rohou & Grigorieff, 2015). Finally, micrographs were manually curated.

For the PTF‐Las1 rixosome dataset 2,328 micrographs were selected, and all further data processing was performed in cryoSPARC (Punjani *et al*, 2017) (see sorting scheme PTF‐Las1, Appendix Fig S2). 1,409,772 particles were obtained using Blob Picker and subjected to 2D classification. Then Topaz (Bepler *et al*, 2019) was trained with particles from good 2D classes for both Rix1 complex and Las1‐Grc3 complex. After another round of 2D classification particles corresponding to each complex were separated and processed individually. For the Rix1 complex (588,317 particle) an ab‐initio model was generated, and low‐quality particles were sorted out by Heterogeneous Refinement. Further sorting yielded the (Rix1)_2_‐(Ipi3)_2_ complex (239,033 particle) and the complex with engaged but flexible Ipi1 (232,826 particle). Finally, particles for both subcomplexes were subjected to Non‐uniform Refinement with defocus and global CTF refinement. For the Las1‐Grc3 complex three additional rounds of Topaz training and 2D classification were performed. An ab‐initio model was generated with the good 2D classes (97,605 particle). 2D classes representing potential side views of the complex (89,802 particle) were added for the Heterogenous Refinement and low‐quality particles were sorted out. The obtained particles were re‐extracted with a box size of 320 pix and 104,264 particles were subjected to a final round of Non‐uniform Refinement with defocus and global CTF refinement. The 2D classes of the Las1‐Grc3 complex showed additional densities at the shorter edge of the butterfly‐shaped complex. Therefore, the box size was increased from 320 to 420 pixels and further sorted by 2D classification. Classes containing both the butterfly like Las1‐Grc3 complex and the spherical Rix1 complex in similar orientations were used for Ab‐Initio Reconstruction and Non‐uniform Refinement leading to the low‐resolution structure of the rixosome.

For the PT‐Rsa4 E117D Nop7‐Flag dataset, 12,836 micrographs were manually selected. 2,110,727 particles were auto‐picked and extracted (3.135 Å/pix) with RELION‐3.1 (Zivanov *et al*, 2018), then imported to cryoSPARC for 2D classification (1,975,047 particle) and initial 3D refinement with EMD‐6615 lowpass filtered to 60 Å as initial reference. The pre‐60S particles were re‐imported into Relion 3.1 (3.135 Å/pix) and subjected to extensive 3D classification and focused 3D classification (see sorting scheme PT‐Rsa4 E117D Nop7‐Flag, Appendix Fig S3) yielding States 1, 2, 5, 6, 7, and 10 (compare Table S2). Particles of the different states were extracted with a box size of 500 pixel and 1.045 Å/pix. For all states, final Homogeneous Refinements including CTF parameter refinement and Local Refinements were performed in cryoSPARC. The PT‐Rsa4 E117D Rix‐Flag dataset was processed using the same general procedure as for the PT‐Rsa4 E117D Nop7‐Flag dataset (Appendix Fig S4). 1,295,971 particles were obtained from 9,982 selected micrographs and after 2D Classification 732,587 particle were used for further processing in Relion 3.1. Classification yielded States 3, 4 and 8 (compare Table S2). For the PT‐Rsa4 E117D Flag‐Las1 dataset the same processing procedure as for the PT‐Rsa4 E117D Nop7‐Flag dataset was applied (Appendix Fig S4). Initially, 402,260 particles were auto‐picked and extracted from 3,534 micrographs. 2D classification (285,836 particle) and initial Homogeneous Refinement were performed in CryoSPARC and 3D classification in Relion 3.1 yielded States 3 and 4 (compare Table S2) were obtained. To improve reconstruction quality and resolution for State 3 and State 4 we combined the corresponding particles sets from the PT‐Rsa4 E117D Rix1‐Flag and the PT‐Rsa4 Flag‐Las1 datasets (see above).

### Model building and refinement

For model building of State 1 (pre‐5S rotation) the equivalent yeast model (PDB‐ID: 3JCT) was rigid body fitted into the cryo‐EM density and used as an initial reference. Homology models of the ribosomal protein were generated with SWISS‐MODEL (Waterhouse *et al*, 2018) and Alpha‐Fold (Jumper *et al*, 2021) predictions were used as initial models for the biogenesis factors. The factors were docked into the density and compared to the yeast models as well as to the model of the mature 80S ribosome from *C. thermophilum* (PDB‐ID: 7OLC). Models were manually adjusted or build *de novo* according to the density map in Coot (Emsley & Cowtan, 2004). The rRNA was manually inspected and mutated into the *C. thermophilum* sequence. For the immature central protuberance with pre‐rotated 5S RNP the yeast model (PDB‐ID: 3JCT) was used as reference. The molecular model of State 1 was used as initial model for the other states and models were manually checked and adjusted in Coot. References from yeast (PDB‐ID: 6YLH) and *C. thermophilum* (PDB‐ID: 7OLC) were used for State 3 and State 4 to build and refine the central protuberance after 5S RNP rotation. For the generation of the Rix1 complex model particles of State 3 and State 4, which differ only in the presence or absence of the Foot, they were combined, and the better resolved local refinement of the Rix1 complex density was used for model building. Alpha‐Fold models of Rix1, Ipi3, and Ipi1 from *C. thermophilum* were rigid body fitted and manually corrected. The resulting model was used for States 2, 3, and 4. Again the model was docked into the densities and manually corrected. For State 10, models for Ytm1, Erb1, Nop7, and uL30 from the nucleolar *C. thermophilum*–State Spb4 (PDB‐ID: 8I9Z) were fitted in the corresponding densities and adjusted with Coot. The model of the L1 stalk containing nucleotides 2,400–2,471 (helix 76–78) of the 26S rRNA and the ribosomal protein uL1 was built with State 3 and State 4. The resulting model was rigid body fitted into the corresponding uL1 stalk density of State 2 and State 5 and refined with Isolde (Croll, 2018). The model was inspected in Coot and manually corrected. All models were real‐space refined in Phenix with secondary structure restraints (Liebschner *et al*, 2019). Cryo‐EM maps and molecular models were visualized using ChimeraX (Pettersen *et al*, 2021).

### Whole genome sequencing and analysis

#### DNA sequencing

The gDNA Illumina libraries were prepared according to the manufacturer (Nextera DNA Flex) and sequenced on an Illumina NovaSeq 6000 by the Bern Genomics Platform (https://www.ngs.unibe.ch). Both the wild‐type strain and the double transformant strain Nr_15 were sequenced at 150 bp PE generating about 20mio reads (raw coverage ~200×) each. The raw reads were deposited at ENA within the project PRJEB46383.

#### Quality control

The QC was performed with FastQC v0.11.7 (https://www.bioinformatics.babraham.ac.uk/projects/fastqc/) revealing excellent quality reads for all samples. The reads were cleaned using fastp v0.19.5 (Chen *et al*, 2018) with a special focus on removing polyG trails and keeping only full‐length reads (150 bp).

#### Reference sequences

The reference genome sequence and its annotations were obtained from ENSEMBLgenomes (Yates *et al*, 2020): CTHT_3.0, INSDC assembly GCA_000221225.1, database version 102.1, Golden path length 28,322,806 bp. The two plasmid sequences pChaetomium1 (HPH1 TpA‐Rsa4 E117D) 9,981 bp and pChaetomium2 (Nop7_FLAG) 8,302 bp were concatenated to the reference genome.

#### Remapping of reads on the reference genome CTHT_3.0

The reference genome was indexed for bwa v0.7.17 (Li & Durbin, 2010). The cleaned reads for each wild‐type and transformant were remapped independently to the reference genome with bwa mem v0.7.17. The final bam files were sorted and indexed with samtools v1.10 (Li, 2011).

#### Plasmid insertions detection

The remapped reads (bam files) were used with different tools to help detecting the plasmid insertion. DELLY v0.8.7 (Rausch *et al*, 2012) and TIDDIT v2.12.0 (Eisfeldt *et al*, 2017) were applied to identify break points and Samclip v0.4.0 (https://github.com/tseemann/samclip) to detect reads with hard or soft clips (partial hits on two chromosomes or plasmids). The clipped reads were filtered using samtools v1.10 to keep only those with a partial hit on the plasmid. The read alignments were manually inspected based on the TIDDIT output with IGV v2.12.2 (Thorvaldsdottir *et al*, 2013) and potential break points were identified and evaluated.

#### Detection by *de novo* genomic assembly

SPAdes v3.12.0 (Bankevich *et al*, 2012) was used to assemble the cleaned reads using various kmers (21,33,55,77,99,127) into a draft genome. The drafts were compared with QUAST v4.6.0 (Gurevich *et al*, 2013). The draft genomes were aligned to the reference + plasmid sequences with Nucmer v4.0.0beta1 (Marcais *et al*, 2018) and the contig with plasmid insertions identified with show‐coords (Nucmer).

#### New reference genome assembly

Using the wild‐type reads, we assembled a draft genome with SPAdes v3.12.0 (Bankevich *et al*, 2012) (295 contigs, N50 729,905 bp, sum 28.41 Mbp) and in a second step, we combined the draft genome with the current reference genome with a tool called MAC2.0 modifying the length option of show‐coords to “‐L 10000” (Tang *et al*, 2019). After gap closing with GapCloser v1.12 (Luo *et al*, 2012), only 2 gaps remained in the mitochondrial chromosome. This led to an assembly of 10 scaffolds, 2 gaps, N50 3.1 Mbp, sum 28.44 Mbp. This assembly (CTHT_4.0) was annotated on the GenSAS platform (Humann *et al*, 2019) for the chromosomes and with PROKKA v1.12 (Seemann, 2014) for the mitochondrial chromosome. BUSCO v4.1.4 (Simao *et al*, 2015) evaluated the completeness against Sordariomyceta_odb9 revealing 84.4% complete genes (0.3% duplication), 9.7% fragmented genes, and 5.9% missing over *n*: 3,725 marker genes.

## Author contributions

**Ed Hurt:** Conceptualization; supervision; funding acquisition; validation. **Roland Beckmann:** Conceptualization; supervision; funding acquisition; validation; investigation. **Matthias Thoms:** Conceptualization; data curation; validation; investigation; visualization; methodology. **Benjamin Lau:** Conceptualization; data curation; validation; investigation; visualization; methodology. **Jingdong Cheng:** Data curation; formal analysis; validation; visualization; methodology. **Lisa Fromm:** Data curation; formal analysis; validation; investigation; visualization; methodology. **Nikola Kellner:** Conceptualization; data curation; formal analysis; validation; investigation; visualization; methodology. **Dirk Flemming:** Data curation; formal analysis; validation; investigation; visualization; methodology. **Paulina Fischer:** Data curation; validation; investigation; visualization. **Timo Denk:** Data curation; formal analysis; validation. **Otto Berninghausen:** Data curation; formal analysis; validation; investigation; methodology. **Laurent Falquet:** Data curation; formal analysis; validation; investigation; visualization; methodology.

## Disclosure and competing interests statement

The authors declare that they have no conflict of interest.

## Supporting information



Appendix

Expanded View Figures PDF

PDF+

Source Data for Figure 1

Source Data for Figure 2

## Data Availability

All cryo‐EM maps and molecular models have been deposited in the Electron Microscopy Data Bank (EMDB) with accession codes EMD‐17956, EMD‐17881, EMD‐17882, EMD‐17883, EMD‐17884, EMD‐17885, and EMD‐17886 for State 1–pre‐5S rotation (Arx1/Nog2 state); EMD‐17953, EMD‐17887, EMD‐17888, EMD‐17889, EMD‐17890, EMD‐17891, and EMD‐17892 for State 2–pre‐5S rotation with Rix1 complex; EMD‐17955, EMD‐17893, EMD‐17894, EMD‐17895, EMD‐17896, EMD‐17897, and EMD‐17898 for State 3–post‐5S rotation with Rix1 complex with Foot; EMD‐17957, EMD‐17899, EMD‐17900, EMD‐17901, and EMD‐17902 for State 4–post‐5S rotation with Rix1 complex without Foot; EMD‐17969, EMD‐17903, EMD‐17904, EMD‐17905, EMD‐17906, and EMD‐17907 for State 5–pre‐5S rotation–L1 inward; EMD‐17950, EMD‐17908, EMD‐17909, and EMD‐17910 for State 6–pre‐5S rotation–L1 intermediate; EMD‐17970, EMD‐17911, EMD‐17912, and EMD‐17913 for State 7–pre‐5S rotation lacking Utp30/ITS2; EMD‐17954, EMD‐17914, and EMD‐17915 for State 8–pre‐5S rotation without Foot; EMD‐17952, EMD‐17916, EMD‐17917, and EMD‐17918 for State 9–pre‐5S rotation–immature H68/H69; EMD‐17951, EMD‐17919, EMD‐17920, EMD‐17921, and EMD‐17922 for State 10–pre‐5S rotation with Ytm1‐Erb1; EMD‐17879 for Rix1 complex ((Rix1)_2_‐(Ipi3)_2_); EMD‐17923 for Rix1 complex ((Rix1)_2_‐(Ipi3)_2_‐Ipi1); EMD‐17949 for Las1‐Grc3 complex; EMD‐17880 for low‐resolution rixosome. Protein Data Bank (PDB) with the accession codes 8PV7 (State 1 ‐ pre‐5S rotation (Arx1/Nog2 state)), 8PV4 (State 2 ‐ pre‐5S rotation with Rix1 complex), 8PV6 (State 3 ‐ post‐5S rotation with Rix1 complex with Foot), 8PV8 (State 4 ‐ post‐5S rotation with Rix1 complex without Foot), 8PVK (State 5 ‐ pre‐5S rotation ‐ L1 inward), 8PV1 (State 6 ‐ pre‐5S rotation ‐ L1 intermediate), 8PVL (State 7 ‐ pre‐5S rotation lacking Utp30/ITS2), 8PV5 (State 8 ‐ pre‐5S rotation without Foot), 8PV3 (State 9 ‐ pre‐5S rotation ‐ immature H68/H69), 8PV2 (State 10 ‐ pre‐5S rotation with Ytm1‐Erb1), 8PTW (Rix1‐complex ‐ (Rix1)_2_‐(Ipi3)_2_) and 8PUW (Las1‐Grc3‐complex). For genome sequencing, raw reads and assembled genome were deposited under ENA project PRJEB46383 with accession IDs ERS6666837 (SAMEA8984131, CTHT4_WT), ERS6666840 (SAMEA8984134, CTHT4_Nr15), ERZ2863203 (CTHT4.fasta).
