# Supplementary material for: Structural insights into coordinating 5S RNP rotation with ITS2 pre‐RNA processing during ribosome formation
Source: EMBO Rep. 2023 Nov 3;24(12):e57984. doi: 10.15252/embr.202357984 (PMC10702828; doi:10.15252/embr.202357984)
Supplement: Supplementary file 1 — Appendix [file EMBR-24-e57984-s002.pdf]

Appendix

Structural Insights into Coordinating 5S RNP Rotation with ITS2 pre-RNA Processing During Ribosome Formation

Matthias Thoms[1,*], Benjamin Lau[2,*], Jingdong Cheng[3] , Lisa Fromm[2], Timo Denk[1], Nikola Kellner[2], Dirk Flemming[2], Paulina Fischer[2], Laurent Falquet[4], Otto Berninghausen[1], Roland Beckmann[1,**] and Ed Hurt[2,**]

Table of content

Appendix Figure S1. Whole genome sequencing of *Chaetomium thermophilum PT-rsa4 E117D NOP7-Flag* split-tag strain. 2

Appendix Figure S2. Cryo-EM data processing scheme, local resolution, and Fourier shell correlation of the PTF-Las1 rixosome dataset. 3

Appendix Figure S3. Cryo-EM data processing scheme of the PT-Rsa4 E117D Nop7-Flag dataset. 5

Appendix Figure S4. Cryo-EM data processing scheme of the PT-Rsa4 E117D Rix-Flag and PT-Rsa4 E117D Flag-Las1 datasets. 6

Appendix Figure S5. Local resolution of the presented pre-60S states. 8

Appendix Figure S6. FSC curves of the presented states and corresponding local refinements.9

Appendix Figure S7. Model validation 10

Appendix Table S1. *C. thermophilum* strains used in this study. 11

Appendix Table S2. Data collection, refinement and model statistics. 12

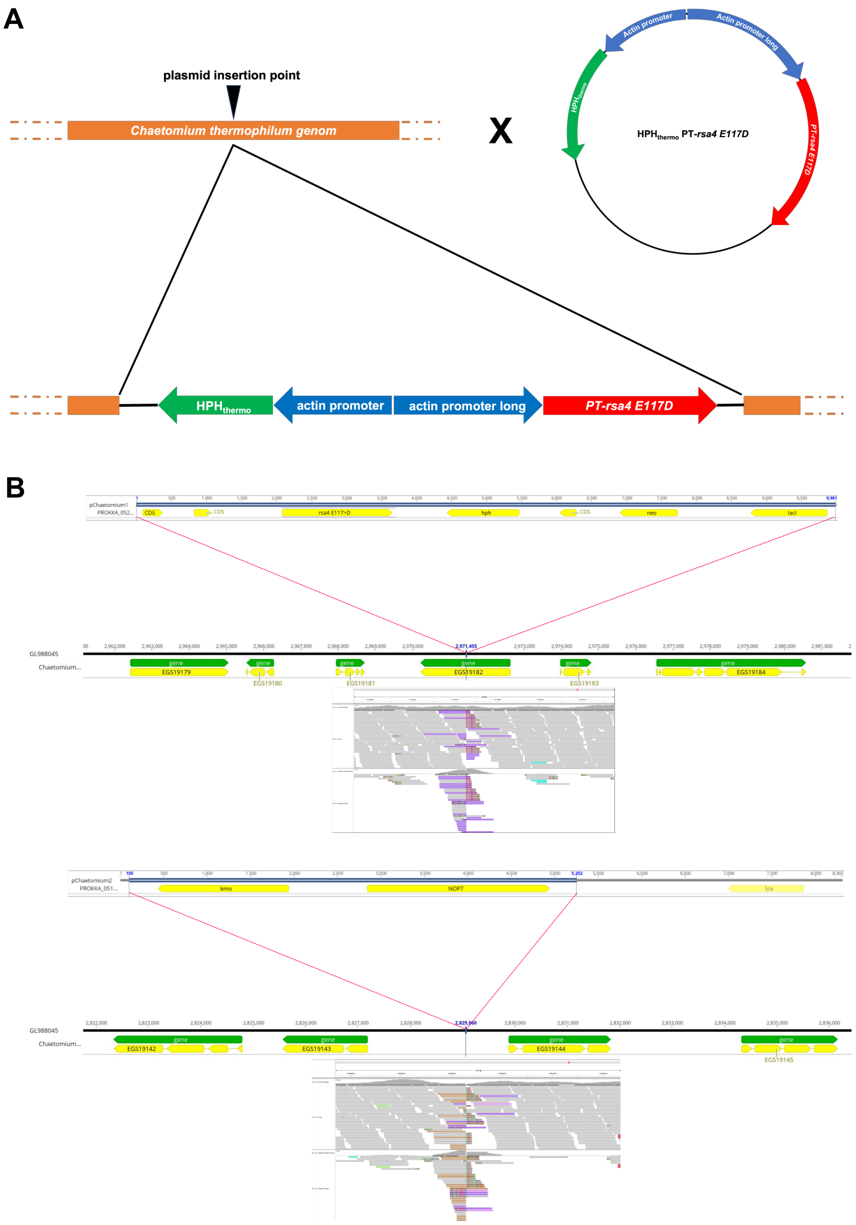

Appendix Figure S1. Whole genome sequencing of *Chaetomium thermophilum PT-rsa4 E117D NOP7-Flag* split-tag strain.

(A) Schematic representation of plasmid insertion into the *Chaetomium thermophilum* genome, here shown for *PT-Rsa4 E117D* together with the heat adapted hygromycin resistance marker.

(B) Whole genome sequencing of the double transformant revealed random insertion of *PT-rsa4 E117D* and *NOP7-Flag* at different genome loci, but each with only one copy per genome. The two plasmids were inserted into the coding region of gene EGS19182 (putative heat shock protein) for pChaetomium1 (upper panel) and into the intergenic region near gene EGS19144 (Beta-hexosaminidase) for pChaetomium2 (lower panel). Colored reads indicate that the mate read is located on a different molecule (brown and purple for the plasmid).

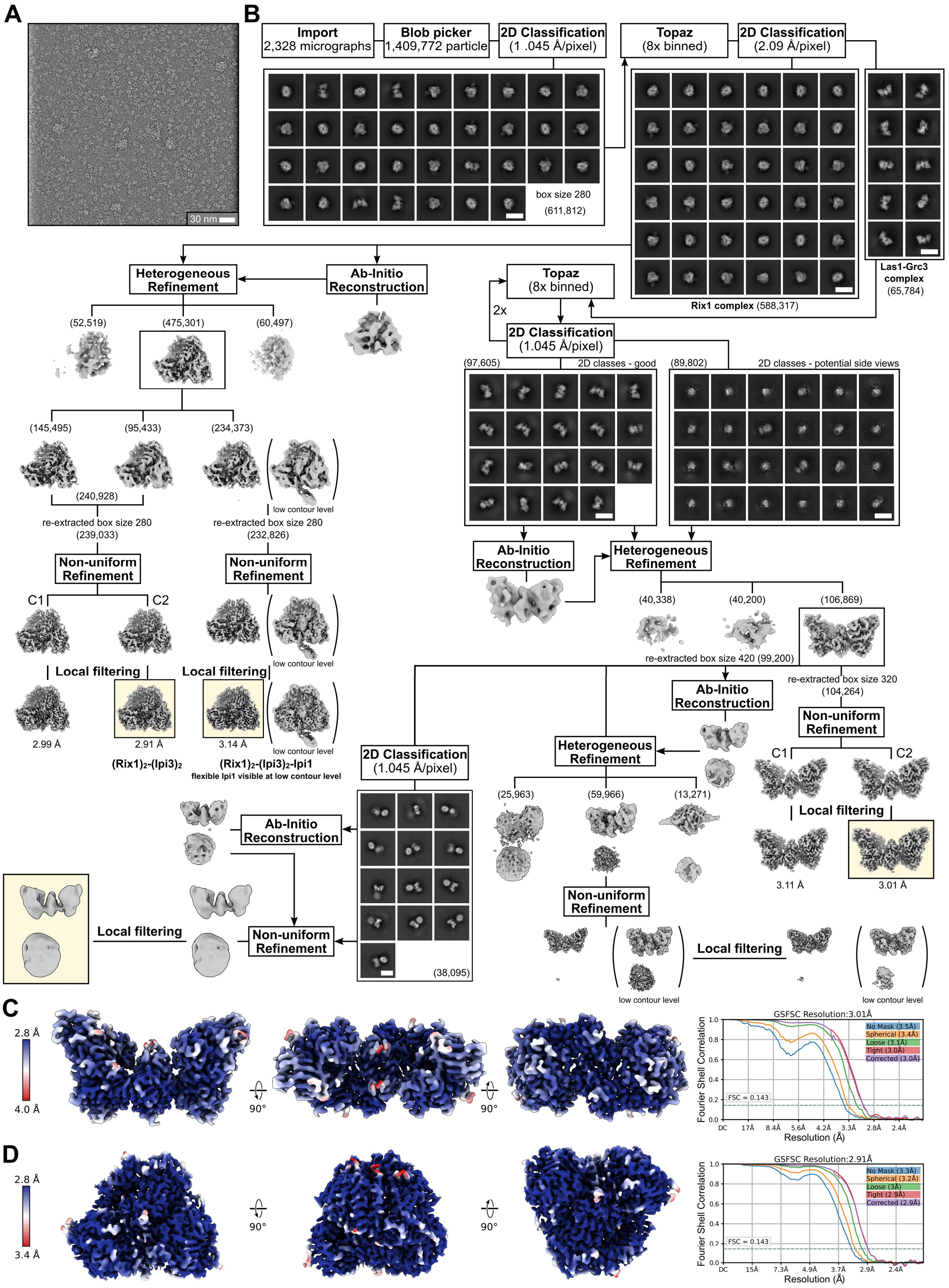

Appendix Figure S2. Cryo-EM data processing scheme, local resolution and Fourier shell correlation of the PTF-Las1 rixosome dataset.

(A) Representative electron micrograph of the PTF-Las1 sample. Scale bar: 30 nm. (B) Data processing and classification scheme. Numbers in brackets indicate the respective particle count. Final classes of the Las1-Grc3 complex, the Rix1 complex and the low-resolution reconstruction of the rixosome are highlighted by yellow boxes. The scale bars for the different 2D classes correspond to 15 nm. (C-D) Local resolution filtered maps colored according to local resolution of the Las1-Grc3 complex (C) and the Rix1 complex (D) and the respective Fourier shell correlation (FSC) curves.

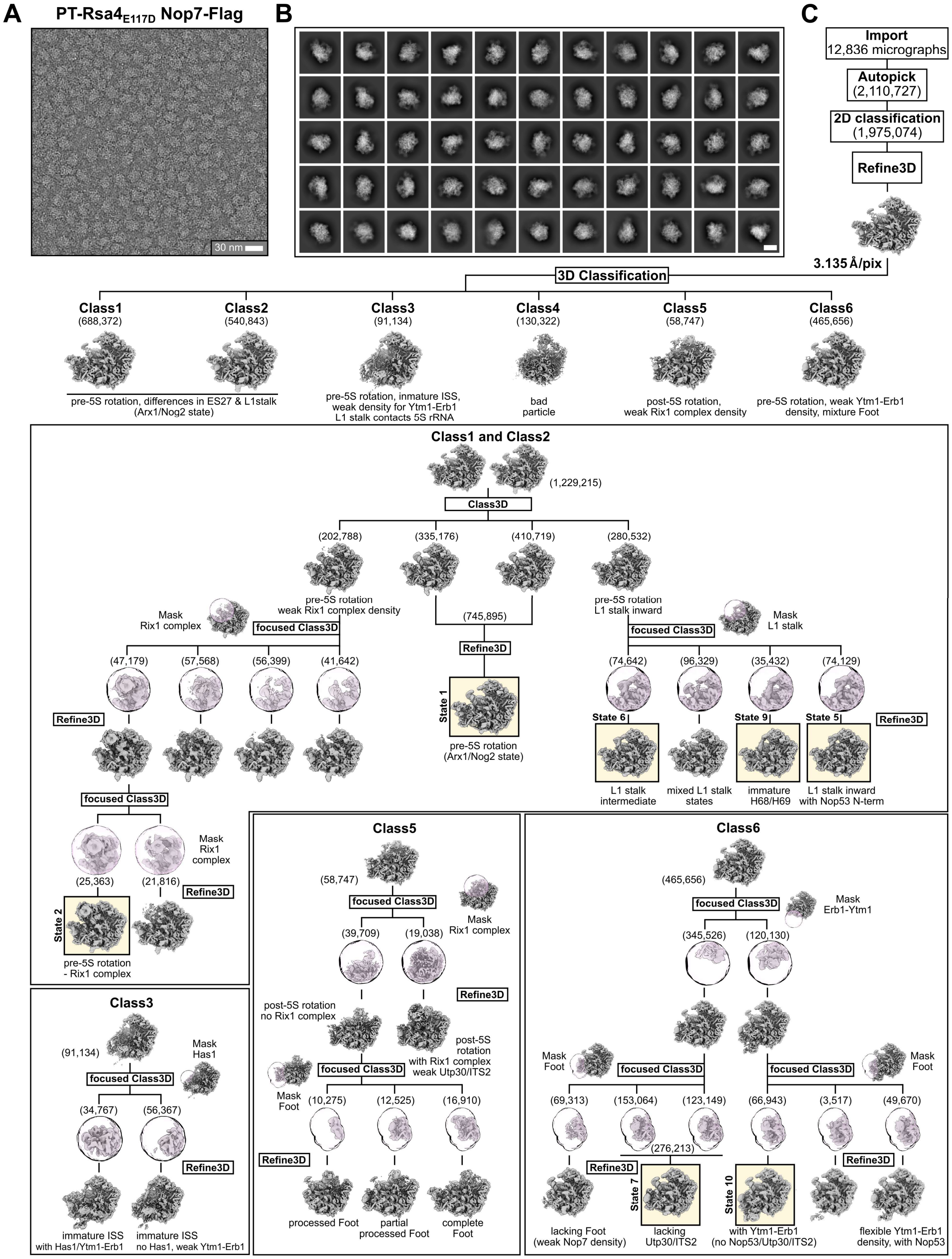

Appendix Figure S3. Cryo-EM data processing scheme of the PT-Rsa4 E117D Nop7-Flag dataset.

(A-B) Representative electron micrograph (A) and 2D class averages (B). Scale bars: 30 nm (A) and 15 nm (B). (C) Data processing and classification scheme of the PT-Rsa4 E117D Nop7-Flag dataset. Particle numbers are shown in brackets and final classes used for undecimated refinement and subsequent local refinements are highlighted by yellow boxes. (ISS – inter subunit space).

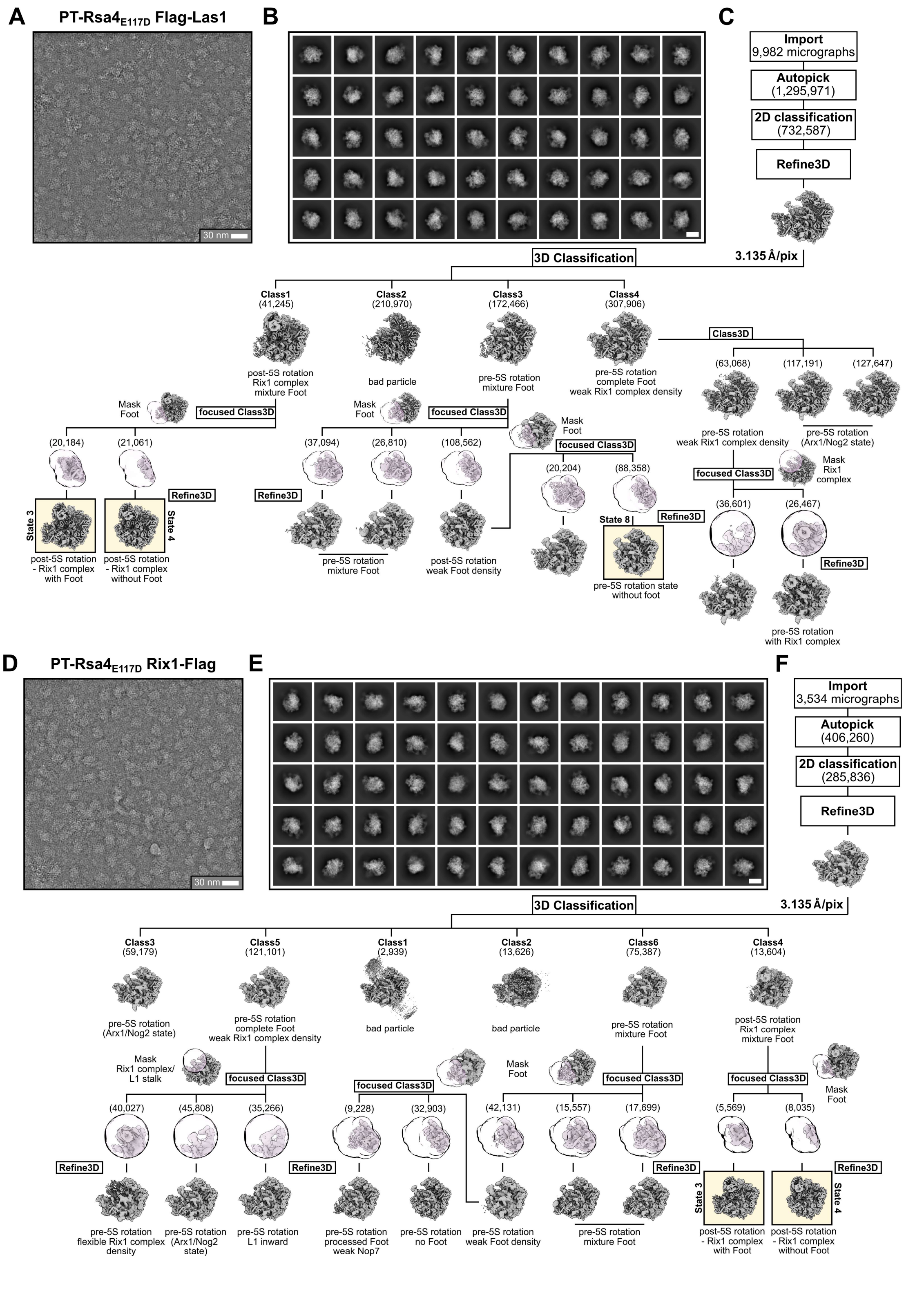

Appendix Figure S4. Cryo-EM data processing scheme of the PT-Rsa4 E117D Rix-Flag and PT-Rsa4 E117D Flag-Las1 datasets.

(A, B, D, E) Representative electron micrographs (A, D) and 2D class averages (B, E) of the PT-Rsa4 E117D Rix-Flag and PT-Rsa4 E117D Flag-Las1 datasets. The scale bars for the electron micrographs and the 2D class averages correspond to 30 nm and 15 nm, respectively. (C, F) Data processing and classification scheme of the PT-Rsa4 E117D Rix-Flag (C) and the PT-Rsa4 E117D Flag-Las1 (F) datasets. Particle numbers are shown in brackets and final classes used for undecimated refinement and subsequent local refinements are highlighted by yellow boxes.

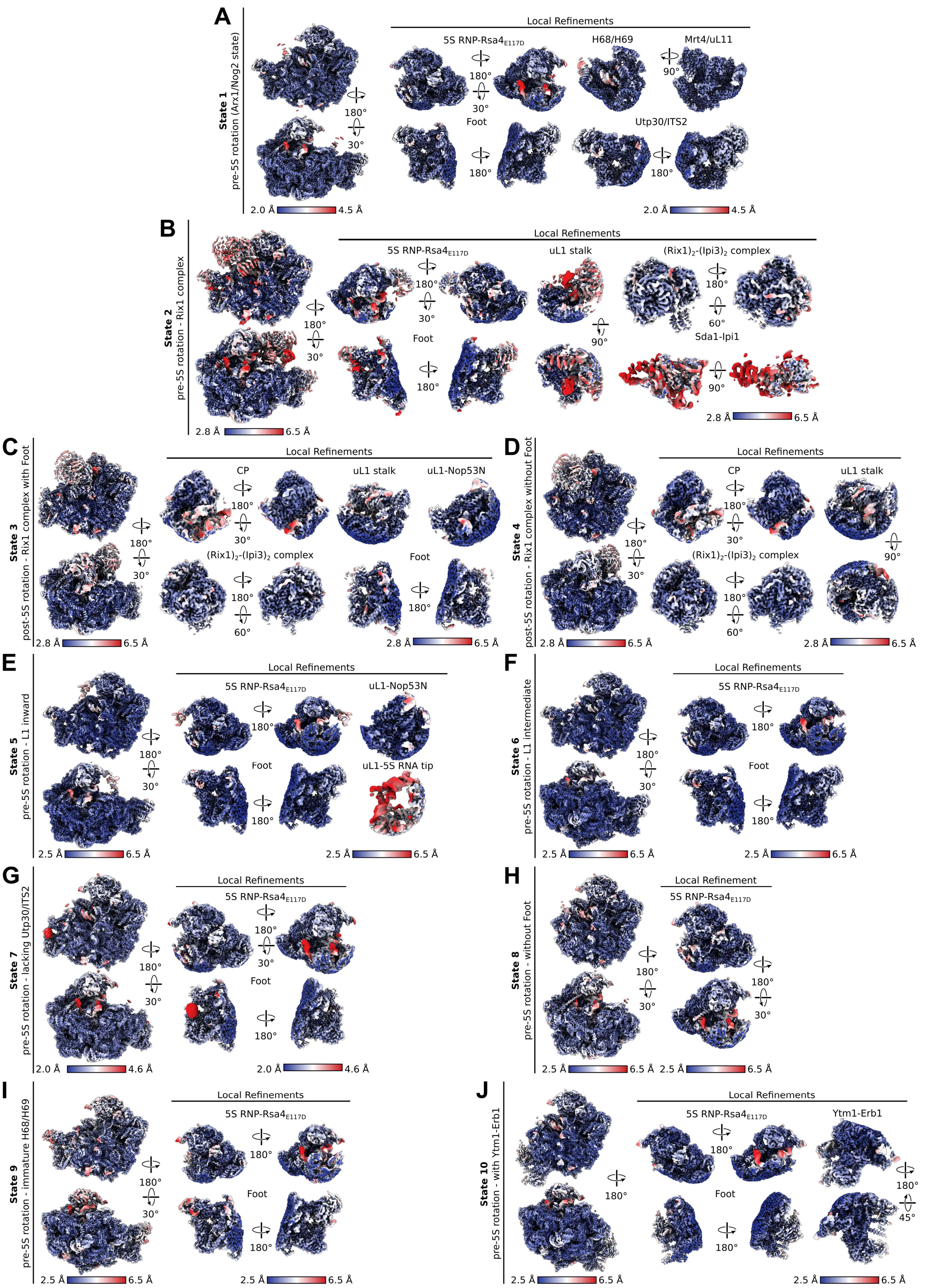

Appendix Figure S5. Local resolution of the presented pre-60S states.

For all states the local resolution filtered maps of the consensus densities and the individual local refinement maps are shown. The maps are colored according to local resolution.

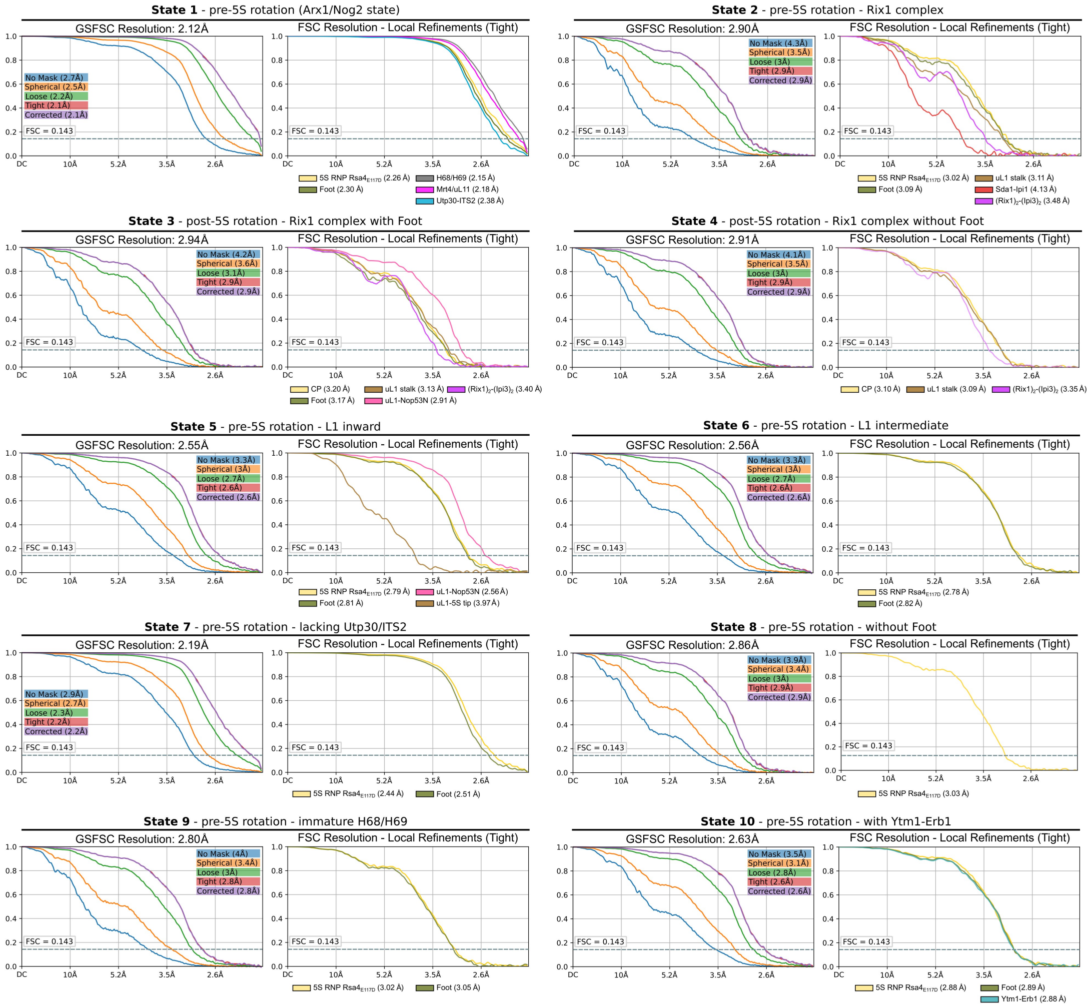

Appendix Figure S6. FSC curves of the presented states and corresponding local refinements. For all states (see Appendix Figure S5) the FSC curves for the consensus reconstructions (left graphs) and the related local refinements (right graphs) are shown. (FSC – Fourier shell correlation)

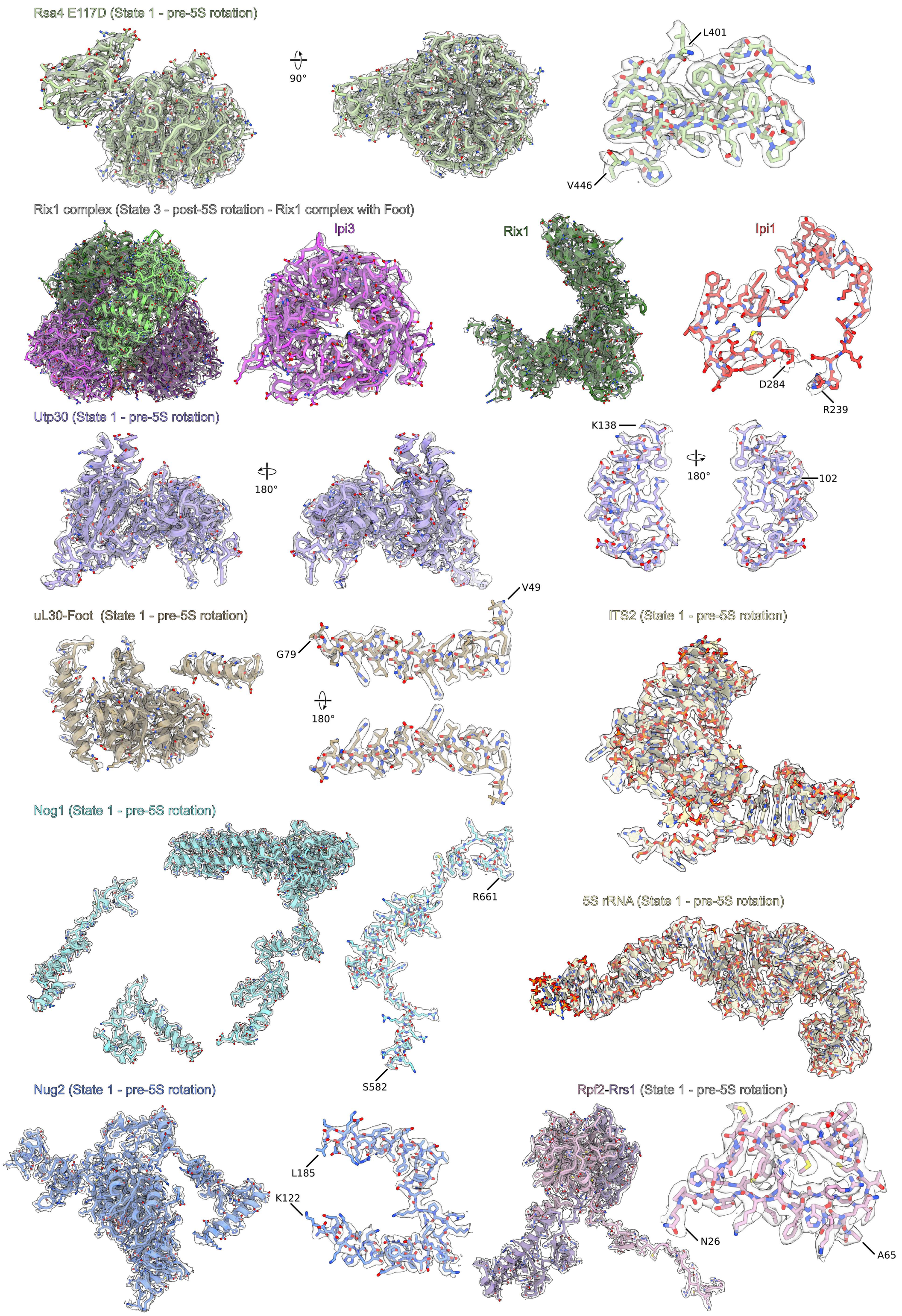

Appendix Figure S7. Model validation

Segmented cryo-EM densities and models of different biogenesis factors, ITS2 and 5S rRNA. The corresponding states are indicated in brackets.

Appendix Table S1. *C. thermophilum* strains used in this study.

Experimental strain	Source	Identifier
Chaetomium thermophilum wild-type strain	DMSZ, Braunschweig	DSM No.: 1495 https://www.dsmz.de
P_{ACT1}-*HPHNT1*-T_{GPD} / P_{ACT1}-PT-*RSA4 E117D*-T_{GPD}, P_{ACT1}-*ERG1*-T_{GPD} / P_{NOT7}-*NOP7*-Flag-T_{GPD}	This study	PT-Rsa4 E117D, Nop7-Flag
P_{ACT1}-*HPHNT1*-T_{GPD} / P_{ACT1}-PT-*RSA4 E117D*-T_{GPD}, P_{ACT1}-*ERG1*-T_{GPD} / P_{RIX1}-*RIX1*-Flag-T_{GPD}	This study	PT-Rsa4 E117D, Rix1-Flag
P_{ACT1}-*HPHNT1*-T_{GPD} / P_{ACT1}-PT-*RSA4 E117D*-T_{GPD}, P_{ACT1}-*ERG1*-T_{GPD} / P_{LAS1}-Flag-*LAS1*-T_{GPD}	This study	PT-Rsa4 E117D, Flag-Las1
P_{ACT1}-*ERG1*-T_{GPD} / P_{ACT1}-PTF-*RSA4 E117D*-T_{GPD}	This study	PTF-Rsa4 E117D #14
P_{ACT1}-*ERG1*-T_{GPD} / P_{ACT1}-PTF-*RSA4 E117D*-T_{GPD}	This study	PTF-Rsa4 E117D #15
P_{ACT1}-*ERG1*-T_{GPD} / P_{LAS1}-PTF-*LAS1*-T_{GPD}	This study	PTF-Las1
P_{ACT1}-*ERG1*-T_{GPD} / P_{NOT7}-*NOP7*-Flag-T_{GPD}	This study	Nop7-Flag
P_{ACT1}-*HPHNT1*-T_{GPD} / P_{ACT1}-PT-*RSA4 E117D*-T_{GPD}	This study	PT-Rsa4 E117D

Appendix Table S2. Data collection, refinement and model statistics

	**State 1** Pre-5S rotation (EMD-17956) (PDB-8PV7)	**State 2** Pre-5S rotation – Rix1 complex (EMD-17953) (PDB-8PV4)	**State 3** Post-5S rotation – Rix1 complex with Foot (EMD-17955) (PDB-8PV6)	**State 4** Post-5S rotation – Rix1 complex without Foot (EMD-17957) (PDB-8PV8)	**State 5** Pre-5S rotation – L1 inward (EMD-17969) (PDB-8PVK)
**Data collection & processing**					
Camera	Gatan K2 Summit	Gatan K2 Summit	Gatan K2 Summit	Gatan K2 Summit	Gatan K2 Summit
Magnification	130,000	130,000	130,000	130,000	130,000
Voltage (kV)	300	300	300	300	300
Electron exposure (e^{-}/Å^2)	45.6	45.6	46.0	46.0	45.6
Defocus range (μm)	0.5 - 3.5	0.5 - 3.5	0.4 - 3.5	0.4 - 3.5	0.5 - 3.5
Pixel size (Å)	1.045	1.045	1.045	1.045	1.045
Symmetry imposed	C1	C1	C1	C1	C1
Micrographs collected (no.)					
Initial particle images (no.)	1,975,074	1,975,074	1,018,423	1,018,423	1,975,074
Final particle images (no.)	745,895	25,363	25,753	29,096	74,129
Map resolution (Å)	2.12	2.90	2.94	2.91	2.55
FSC threshold	0.143	0.143	0.143	0.143	0.143
**Refinement**					
Model resolution (Å)	2.1	3.0	2.9	2.9	2.5
FSC threshold	0.5	0.5	0.5	0.5	0.5
Map sharpening B factor (Å^2)	-65	-55	-55	-60	-60
Model composition					
Non-hydrogen atoms	157,262	178,878	172,014	163,368	160,598
Protein residues	10,514	13,274	12,679	11,673	10,777
Nucleotide residues	3,435	3,493	3,374	3,350	3,495
Ligands	10	10	10	10	10
R.m.s deviations					
Bond lengths (Å)	0.005	0.003	0.003	0.003	0.004
Bond angles (°)	0.753	0.678	0,683	0.689	0.718
Validation					
Molprobity score	1.04	1.16	1.16	1.18	1.10
Clash score	2.54	3.71	3.69	3.88	3.07
Poor rotamers (%)	0.91	0.00	0.02	0.04	0.02
Ramachandran plot					
Favored (%)	98.69	98.37	98.30	98.32	98.46
Allowed (%)	1.31	1.63	1.70	1.67	1.53
Disallowed (%)	0.00	0.00	0.01	0.02	0.01
Map vs. Model CC (mask)	0.88	0.88	0.88	0.88	0.88
**Local & Consensus Refinements**	EMD-17881 EMD-17882 EMD-17883 EMD-17884 EMD-17885 EMD-17886	EMD-17887 EMD-17888 EMD-17889 EMD-17890 EMD-17891 EMD-17892	EMD-17893 EMD-17894 EMD-17895 EMD-17896 EMD-17897 EMD-17898	EMD-17899 EMD-17900 EMD-17901 EMD-17902	EMD-17903 EMD-17904 EMD-17905 EMD-17906 EMD-17907

	State 6<br>Pre-5S rotation – L1 intermediate<br>(EMD-17950)<br>(PDB-8PV1)	State 7<br>Pre-5S rotation – lacking Utp30/ITS2<br>(EMD-17970)<br>(PDB-8PVL)	State 8<br>Pre-5S rotation – without Foot<br>(EMD-17954)<br>(PDB-8PV5)	State 9<br>Pre-5S rotation – immature H68/H69<br>(EMD-17952)<br>(PDB-8PV3)	State 10<br>Pre-5S rotation – with Ytm1-Erb1<br>(EMD-17951)<br>(PDB-8PV2)
**Data collection & processing**					
Camera	Gatan K2 Summit	Gatan K2 Summit	Gatan K2 Summit	Gatan K2 Summit	Gatan K2 Summit
Magnification	130,000	130,000	130,000	130,000	130,000
Voltage (kV)	300	300	300	300	300
Electron exposure (e^-/Å²)	45.6	45.6	46.0	45.6	45.6
Defocus range (μm)	0.5 - 3.5	0.5 - 3.5	0.4 - 3.5	0.5 - 3.5	0.5 - 3.5
Pixel size (Å)	1.045	1.045	1.045	1.045	1.045
Symmetry imposed	C1	C1	C1	C1	C1
Micrographs collected (no.)					
Initial particle images (no.)	1,975,074	1,975,074	732,587	1,975,074	1975,074
Final particle images (no.)	74,642	276,213	88,358	35,432	66,943
Map resolution (Å)	2.56	2.19	2.86	2.80	2.63
FSC threshold	0.143	0.143	0.143	0.143	0.143
**Refinement**					
Model resolution (Å)	2.5	2.2	2.9	2.8	2.6
FSC threshold	0.5	0.5	0.5	0.5	0.5
Map sharpening B factor (Å^2)	-65	-60	-65	-60	-60
Model composition					
Non-hydrogen atoms	157,250	152,760	144,398	155,443	155,881
Protein residues	10,512	10,162	9,204	10,501	10,783
Nucleotide residues	3,435	3,356	3.331	3,355	3,328
Ligands	10	10	10	10	10
R.m.s deviations					
Bond lengths (Å)	0.003	0.008	0.003	0.004	0.005
Bond angles (°)	0.680	0.920	0.682	0.688	0.733
Validation					
Molprobity score	1.10	1.02	1.20	1.13	1.11
Clash score	3.09	2.41	4.20	3.37	3.15
Poor rotamers (%)	0.02	0.91	0.01	0.02	0.02
Ramachandran plot					
Favored (%)	98.42	98.58	98.26	98.51	98.24
Allowed (%)	1.57	1.42	1.73	1.49	1.76
Disallowed (%)	0.01	0.00	0.01	0.00	0.00
Map vs. Model CC (mask)	0.88	0.90	0.81	0.86	0.89
**Local & Consensus Refinements**	EMD-17908<br>EMD-17909<br>EMD-17910	EMD-17911<br>EMD-17912<br>EMD-17913	EMD-17914<br>EMD-17915	EMD-17916<br>EMD-17917<br>EMD-17918	EMD-17919<br>EMD-17920<br>EMD-17921<br>EMD-17922

	**Las1-Grc3 complex** (EMD-17949) (PDB-8PUW)	**Rix1-complex** $(Rix1)_2$-$(Ipi3)_2$ (EMD-17879) (PDB-8PTW)	**Rix1-complex** $(Rix1)_2$-$(Ipi3)_2$-Ipi1 (EMD-17923)	**Rixosome** (EMD-17880)
**Data collection & processing**				
Camera	Gatan K2 Summit	Gatan K2 Summit	Gatan K2 Summit	Gatan K2 Summit
Magnification	130,000	130,000	130,000	130,000
Voltage (kV)	300	300	300	300
Electron exposure (e^-/Å²)	43.6	43.6	43.6	43.6
Defocus range (μm)	0.5 - 3.5	0.5 - 3.5	0.5 - 3.5	0.5 - 3.5
Pixel size (Å)	1.045	1.045	1.045	1.045
Symmetry imposed	C2	C2	C1	C1
Micrographs collected (no.)				
Initial particle images (no.)	187,407	588,317	588,317	104,264
Final particle images (no.)	104,264	239,033	232,826	38,095
Map resolution (Å)	3.01	2.91	3.14	10.02
FSC threshold	0.143	0.143	0.143	0.143
**Refinement**				
Model resolution (Å)	3.0	3.0		
FSC threshold	0.5	0.5		
Map sharpening B factor (Å²)	-80	-80		
Model composition				
Non-hydrogen atoms	11,160	14,242		
Protein residues	1,442	1,878		
Nucleotide residues	0	0		
Ligands	0	0		
R.m.s deviations				
Bond lengths (Å)	0.004	0.04		
Bond angles (°)	0.693	0.784		
Validation				
Molprobity score	1.10	1.18		
Clash score	3.07	3.99		
Poor rotamers (%)	0.00	0.00		
Ramachandran plot				
Favored (%)	98.44	98.17		
Allowed (%)	1.56	1.83		
Disallowed (%)	0.00	0.00		
Map vs. Model CC (mask)	0.88	0.81		
