# Supplementary material for: Structural insights into coordinating 5S RNP rotation with ITS2 pre‐RNA processing during ribosome formation
Source: EMBO Rep. 2023 Nov 3;24(12):e57984. doi: 10.15252/embr.202357984 (PMC10702828; doi:10.15252/embr.202357984)
Supplement: Supplementary file 2 — Expanded View Figures PDF [file EMBR-24-e57984-s005.pdf]

Expanded View Figures

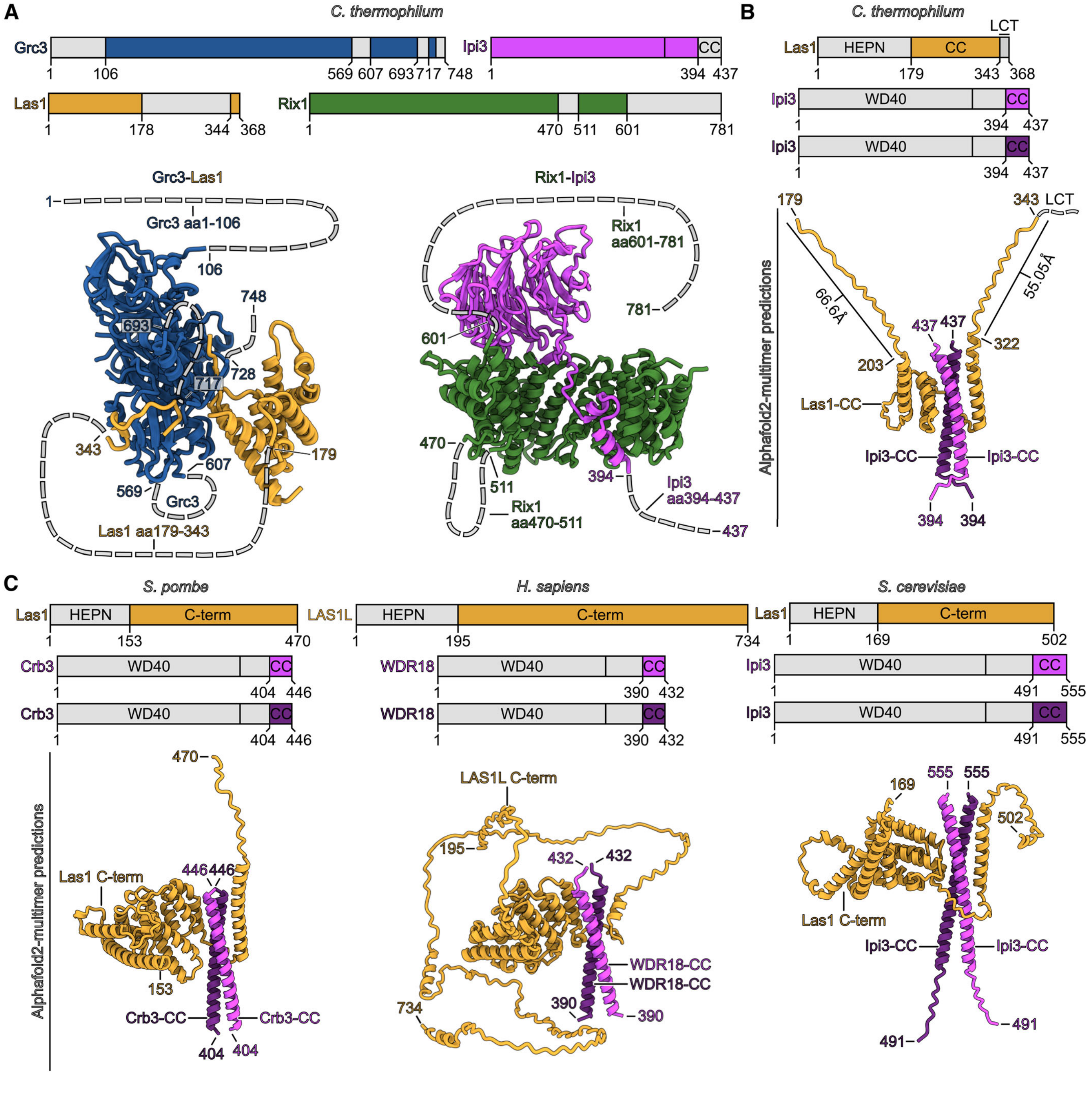

Figure EV1. Alphafold predictions of the linker regions between Rix1 complex and Las1-Grc3 complex within the rixosome.

A Schematic representation and molecular models of Grc3, Las1, Ipi3, and Rix1 shown in different colors. Regions not included in the molecular models are indicated in gray.

B Alphafold2-multimer model of the Las1 coiled-coil domain (Las1-CC, aa179-343) in complex with a dimer of the C-terminal coiled-coil helix of Ipi3 (Ipi3-CC, aa394-437). The maximal length of the unstructured regions are indicated in Å.

C Alphafold2-multimer predictions of the Las1 C-term/Ipi3-CC interactions from *S. pombe*, *H. sapiens*, and *S. cerevisiae*.

© 2023 The Authors

Figure EV2. Structural comparison of Arx1 and foot.

A Nucleoplasmic pre-60S particle from *C. thermophilum* lack the polypeptide exit tunnel (PET) bound export factor Arx1. Comparison between nucleoplasmic pre-60S states from *ct* (left, state 1) and yeast (middle, PDB-ID: 7UOO) and cytoplasmic pre-60S particles after release of Nog1 and Rlp24 from human bound to EBP1 (human Arx1 homolog, right, PDB-ID: 6LSR). The upper panels show the molecular models of the different states, and the lower panels focus on the polypeptide exit tunnel.

B Superposition of Arx1-Alb1 model from yeast and the Ebp1 model from human onto the pre-5S rotation state from *C. thermophilum* (state 1). The Nog1 C-terminus from *C. thermophilum* sterically clashes with the binding sites of Arx1 and EBP1 rationalizing the lack of *ct* Arx1 on the here described nucleoplasmic pre-60S states.

C Comparison of the *ct* ITS2 containing Foot structure (state 1) and the equivalent yeast state ($Nog2_{pre}$, PDB-ID: 7UOO).

D Colored cryo-EM map of the *ct* pre-5S rotation state 1 in two orientations highlighting the two copies of uL30 within the particle (upper panels). Detailed view of the individual uL30 copies (lower panel, left and middle) and merge of the two copies (lower panel, right). The N-terminus of $uL30_{Foot}$ has to rotate in comparison to the uL30 copy within the pre-60S core to adapt the binding mode of Rlp7 within the yeast Foot.

E Structural comparison of ct Utp30 bound to helix 2 (H2) of the ITS2 within the Foot structure, *ct* Utp30 as part of the *ct* 90S bound to es10 (PDB-ID: 6RXU) and the yeast foot factor Nsa3 bound to the H2 of the ITS2 (PDB-ID: 7UOO). The models are labeled and shown in two orientations and as merge comparing pre-60S *ct* Utp30/ITS2-H2 with 90S *ct* Utp30/es10 and pre-60S *sc* Nsa3/ITS2-H2, respectively.

© 2023 The Authors

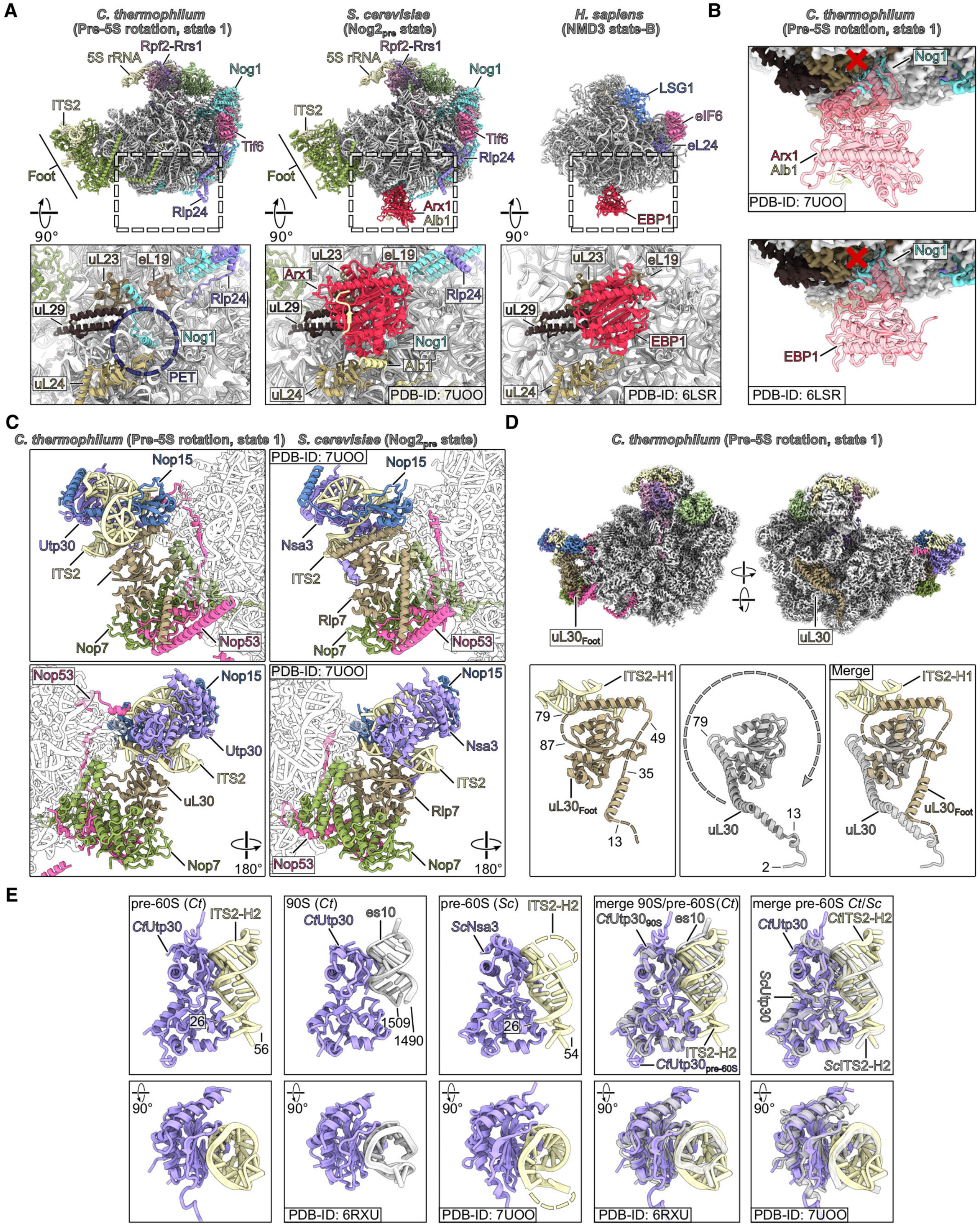

Figure EV2.

 © 2023 The Authors

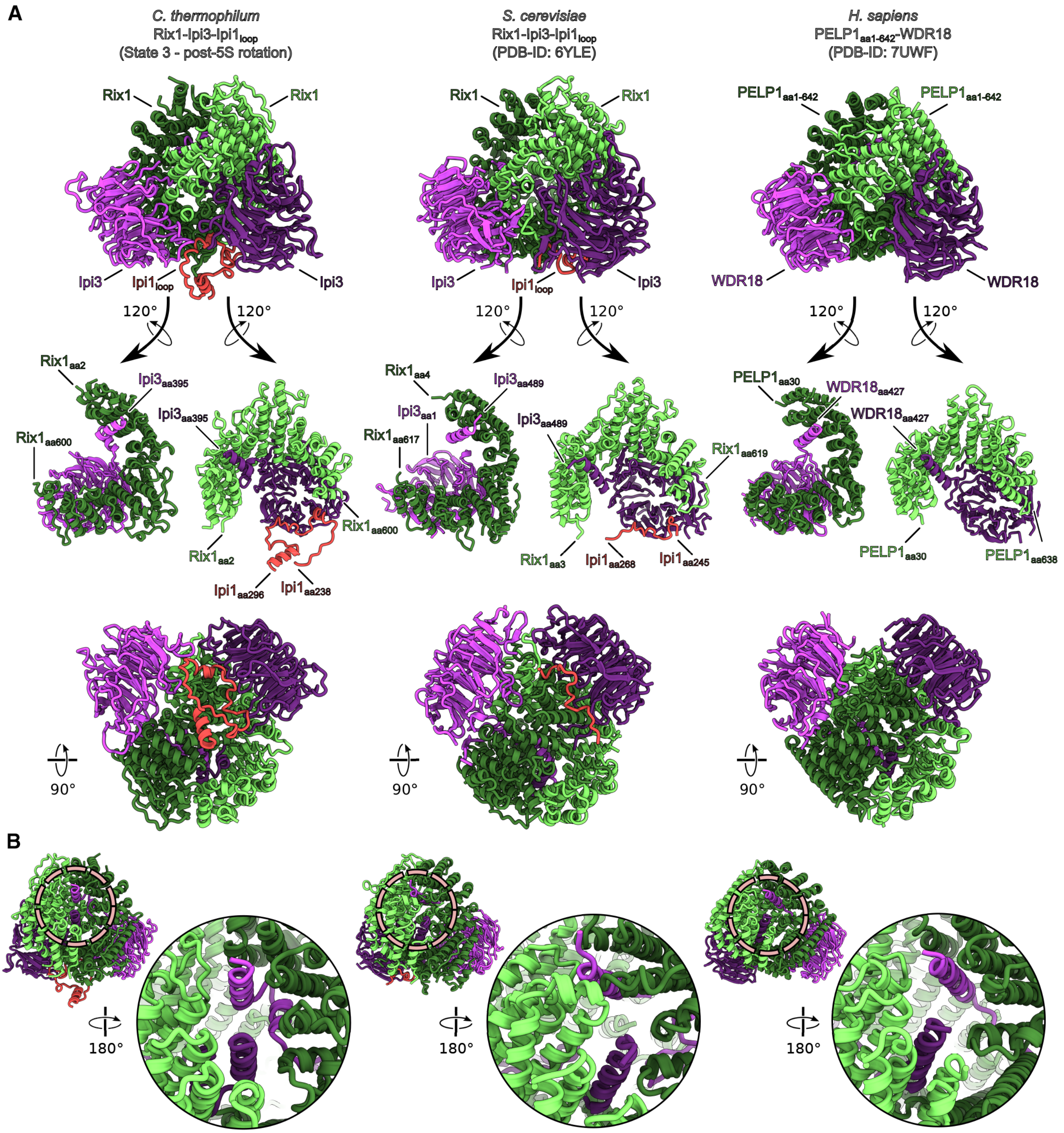

Figure EV3. Comparison of the Rix1 complex structures from *C. thermophilum* and *S. cerevisiae* and the human PELP1-WDR18 complex.

A The Rix1 complex shows strong structural conservation between organisms. Comparison of the molecular models of the Rix1 complex from *C. thermophilum*, *S. cerevisiae*, and the PELP1-WDR18 complex from *H. sapiens*.

B Detailed view of the C-termini of Ipi3/WDR18 for each species. The termini are similarly positioned and able to protrude from the Rix1 complex, potentially form coiled-coils and interact with Las1 (see Fig EV1).

Figure EV4. Additional states.

A–E Colored cryo-EM maps of additional pre-60S states identified in the study. (A) Close-ups showing the colored density map of the partly processed foot structure of the pre-5S rotation-lacking Utp30/ITS2 state. Superimposed models of the pre-5S rotation state (Arx1/Nog2 state) indicating the missing parts: Utp30, ITS2, the C-terminal part of Nop15 (aa277-335), and Nop53 (aa85-107). (D) Close-up of the immature H68/H69 area with superimposed transparent model of H68 and H69 of the pre-5S rotation state (Arx1/Nog2 state).

 © 2023 The Authors

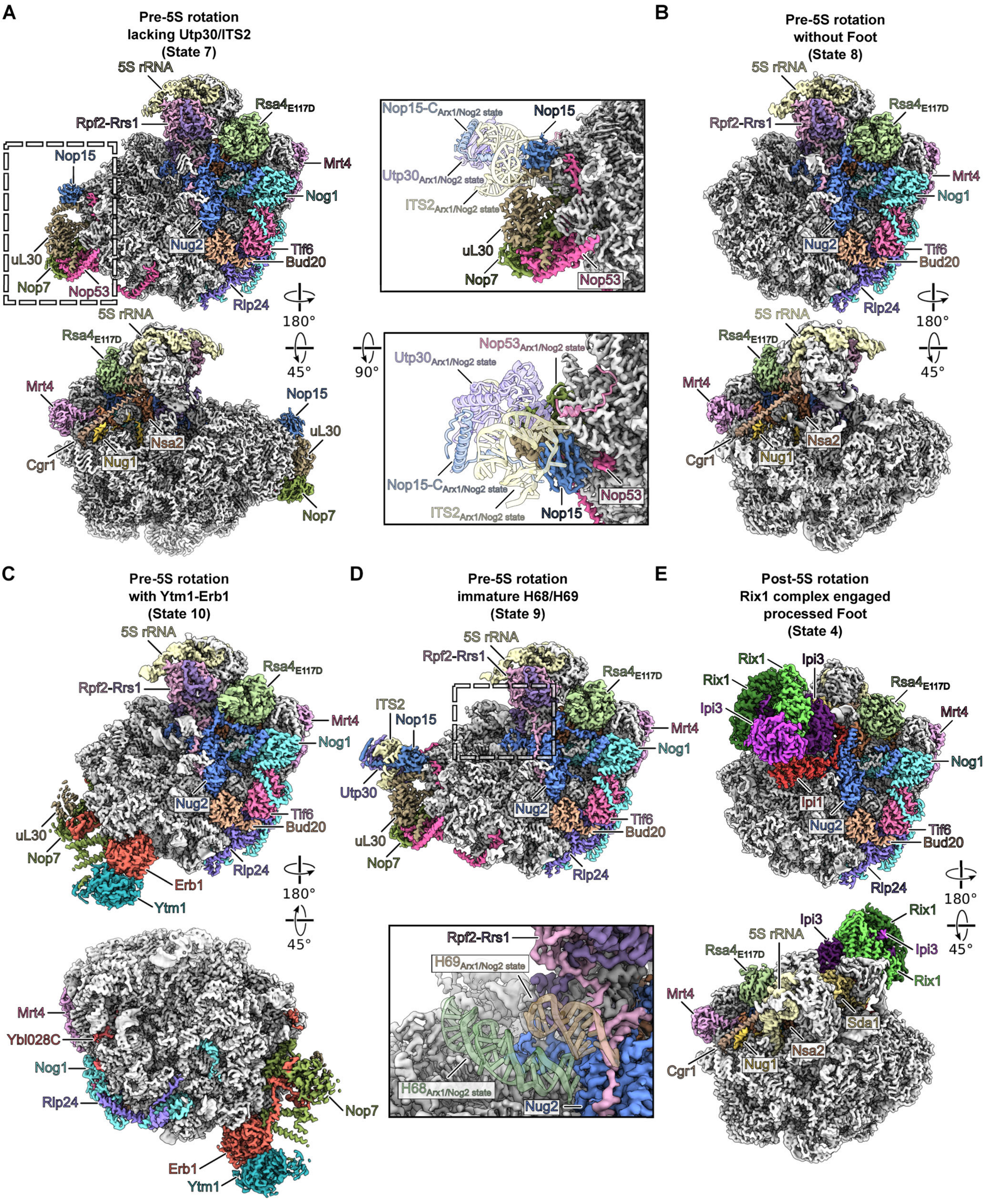
A
Pre-5S rotation
lacking Utp30/ITS2
(State 7)
5S rRNA
Rpf2-Rrs1
Rsa4E117D
Nop15
Mrt4
Nog1
Nug2
Tif6
Bud20
uL30
Nop7
Nop53
Rlp24
180°
45°
Cgr1
Nug1
Nsa2
Nop15-CArx1/Nog2 state
Utp30Arx1/Nog2 state
ITS2Arx1/Nog2 state
90°
Nop53Arx1/Nog2 state
B
Pre-5S rotation
without Foot
(State 8)
C
Pre-5S rotation
with Ytm1-Erb1
(State 10)
Erb1
Ytm1
Ybl028C
D
Pre-5S rotation
immature H68/H69
(State 9)
ITS2
Utp30
H69Arx1/Nog2 state
H68Arx1/Nog2 state
E
Post-5S rotation
Rix1 complex engaged
processed Foot
(State 4)
Rix1
Ipi3
Ipi1
Sda1

Figure EV4.

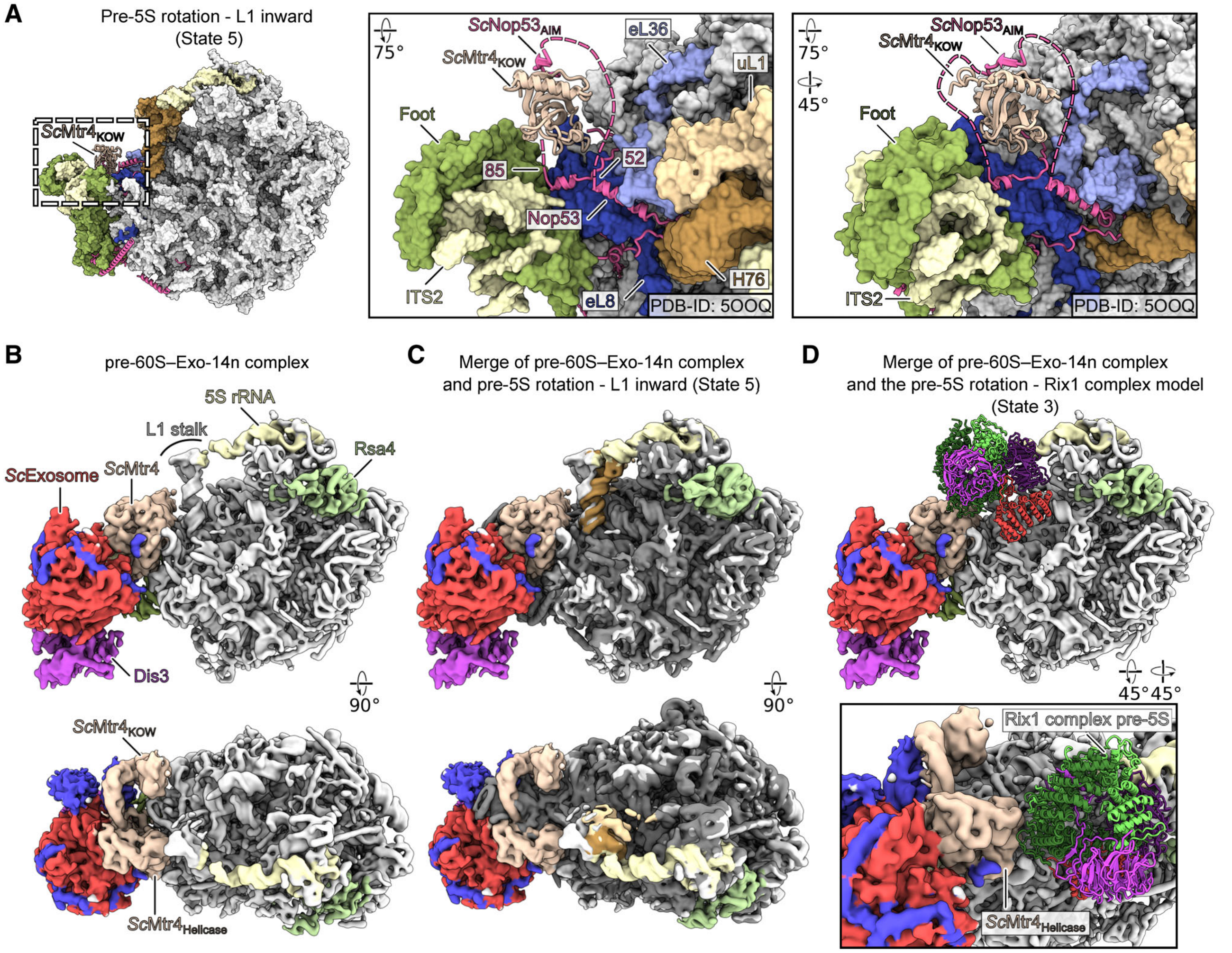

Figure EV5. Structural comparison with the pre-60S-Exo-14n complex.

A Colored surface representation of the pre-5S rotation state with L1 in the inward position (State 5). Nop53 is shown as model. The superimposed KOW domain together with the Nop53 AIM motif from *S. cerevisiae* is shown (PDB-ID: 5OOQ). Only aa666-818 Mtr4-KOW and aa59-70 Nop53-AIM are shown for clarity. The model was rigid body fitted into the merged pre-60S-Exo-14n structure. Dashed lines indicate the flexible, unmodeled parts of Nop53 wrapping around the Mtr4-KOW domain.

B Filtered composite map of the pre-60S-Exo-14n complex from *S. cerevisiae* arrested at the 5.8S+30 pre-rRNA step (EMDB-IDs: 4301 and 4302, PDB-IDs: 6FT6 and 6FSZ).

C The filtered pre-60S-Exo-14n complex composite map superimposed with the *C. thermophilum* pre-5S rotation–L1 inward state (State 5) shown in dark gray and the L1 stalk, 5S rRNA, and Rsa4 in brown, yellow, and green, respectively.

D Overview (upper panel) and detailed view (lower panel) of the merge between the pre-60S-Exo-14n density and the pre-5S rotation state with engaged Rix1 complex (State 3). Only the Rix1 complex model is shown for clarity. Mtr4 and the Rix1 complex do not sterically interfere with each other.

 © 2023 The Authors
