# Supplementary figures and images for: Structural insights into coordinating 5S RNP rotation with ITS2 pre‐RNA processing during ribosome formation
Source: EMBO Rep. 2023 Nov 3;24(12):e57984. doi: 10.15252/embr.202357984 (PMC10702828; doi:10.15252/embr.202357984)

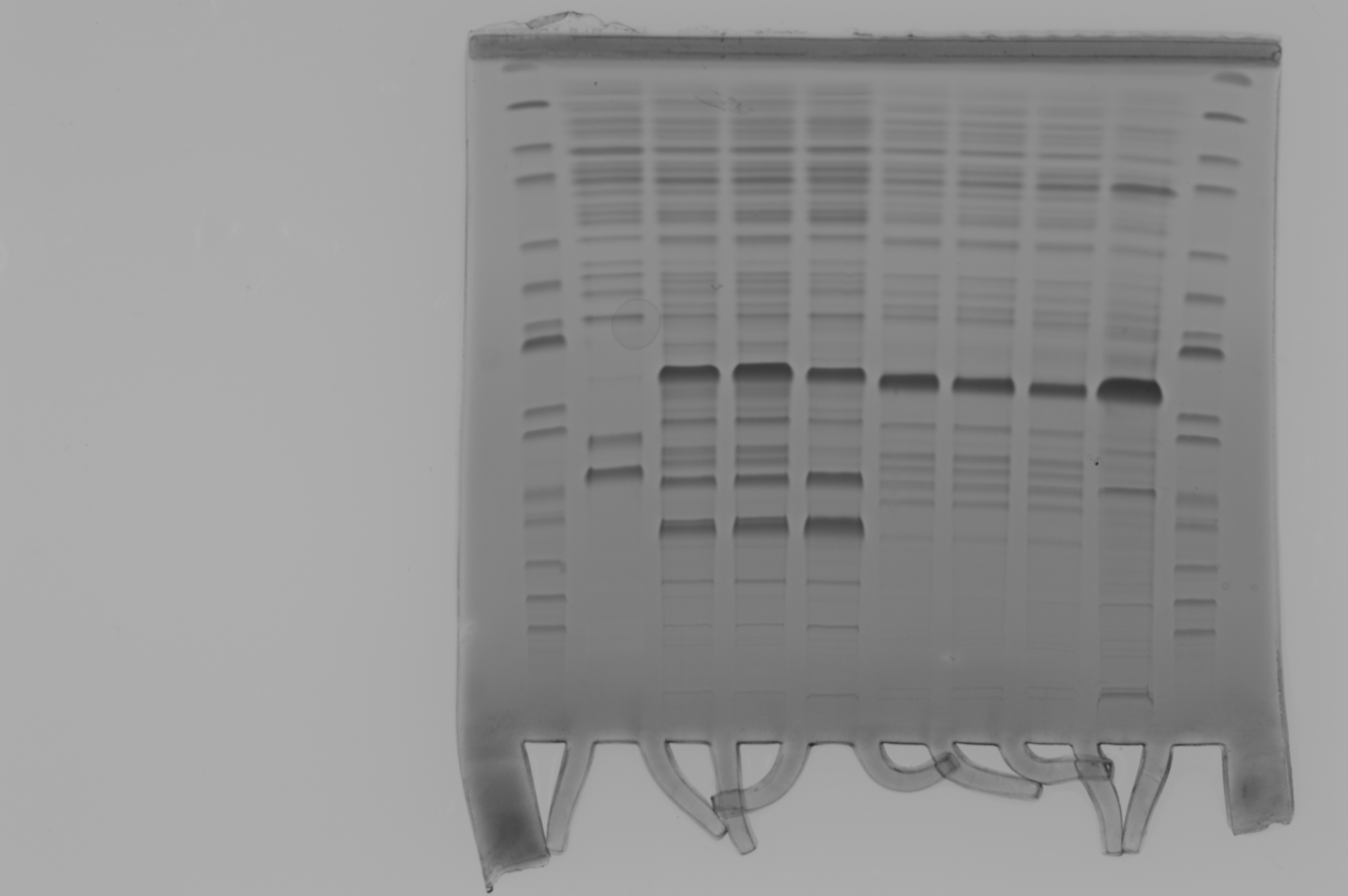

Supplement: Supplementary file 4 — Source Data for Figure 1 [file EMBR-24-e57984-s006.zip › Source Data Figure 1/1A/Source data Fig 1A SDS-PAGE FT-Rsa4.JPG]

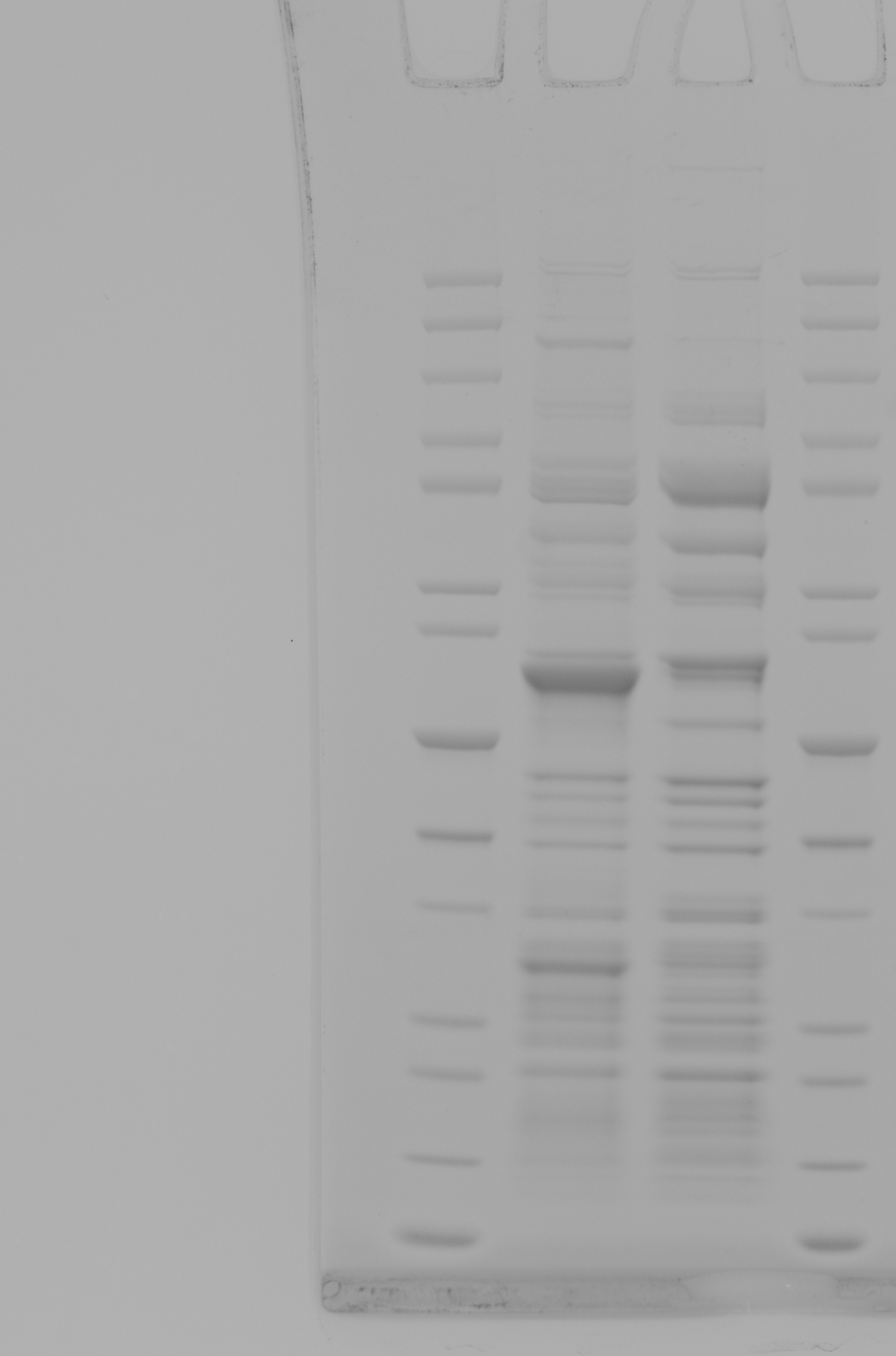

Supplement: Supplementary file 4 — Source Data for Figure 1 [file EMBR-24-e57984-s006.zip › Source Data Figure 1/1B/Source Data Fig 1B SDS-PAGE Rsa4-Nop7.JPG]

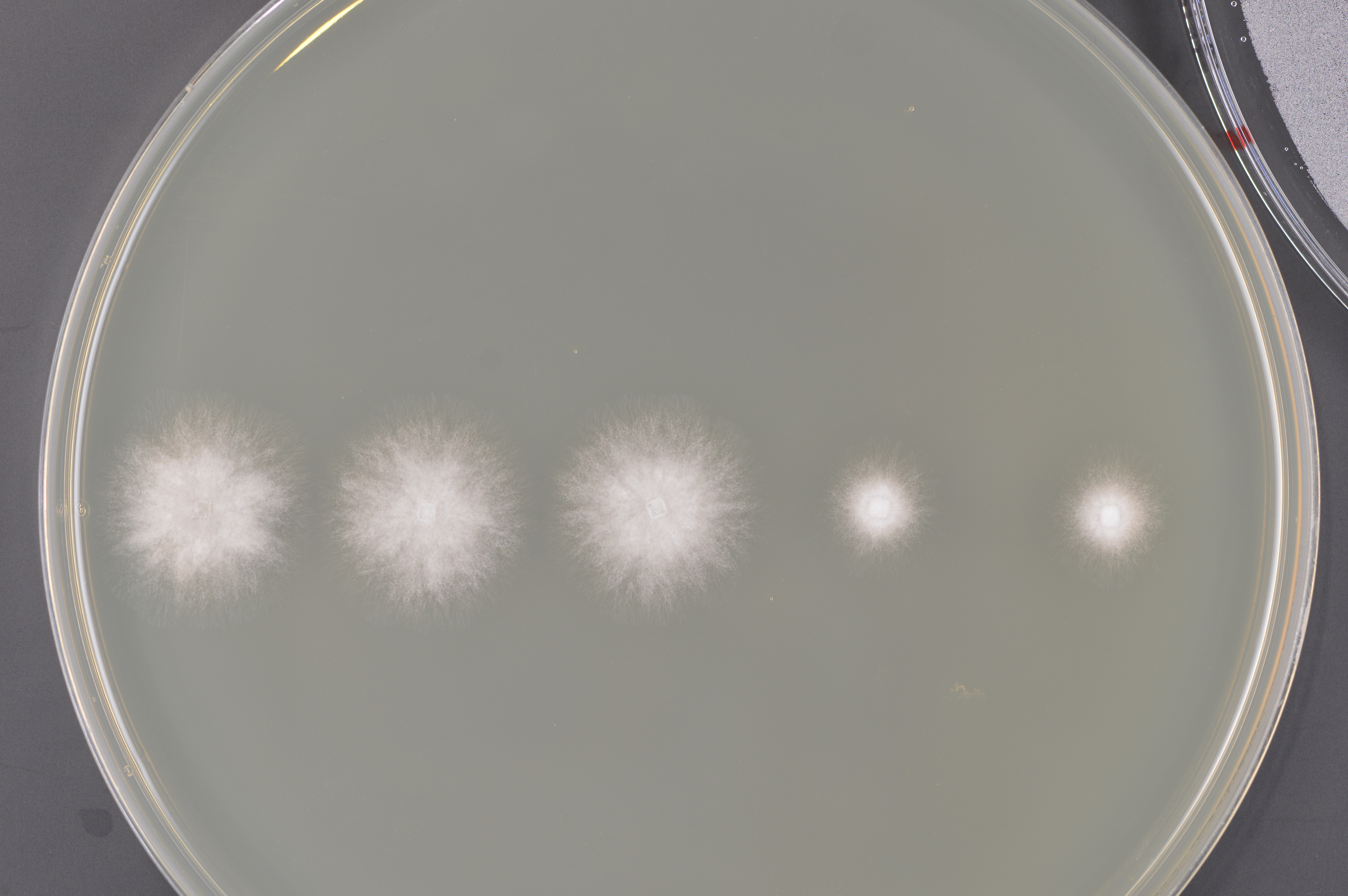

Supplement: Supplementary file 4 — Source Data for Figure 1 [file EMBR-24-e57984-s006.zip › Source Data Figure 1/1C/Source Data Fig 1C dot spot.JPG]

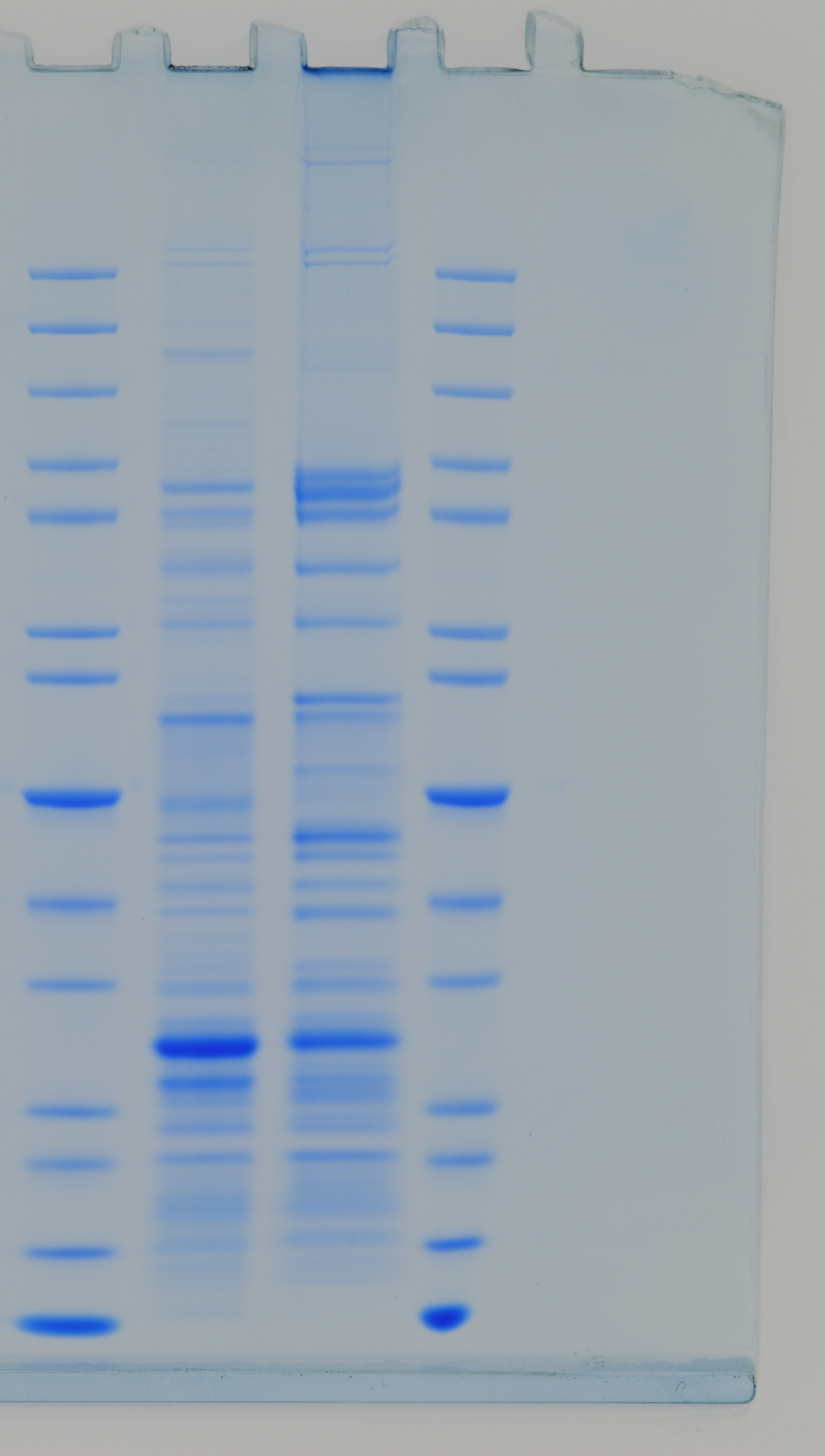

Supplement: Supplementary file 5 — Source Data for Figure 2 [file EMBR-24-e57984-s004.zip › Soruce Data Figure 2/2A/Source Data Fig 2A SDS_1_left_panel_Rsa4_Rix1.JPG]

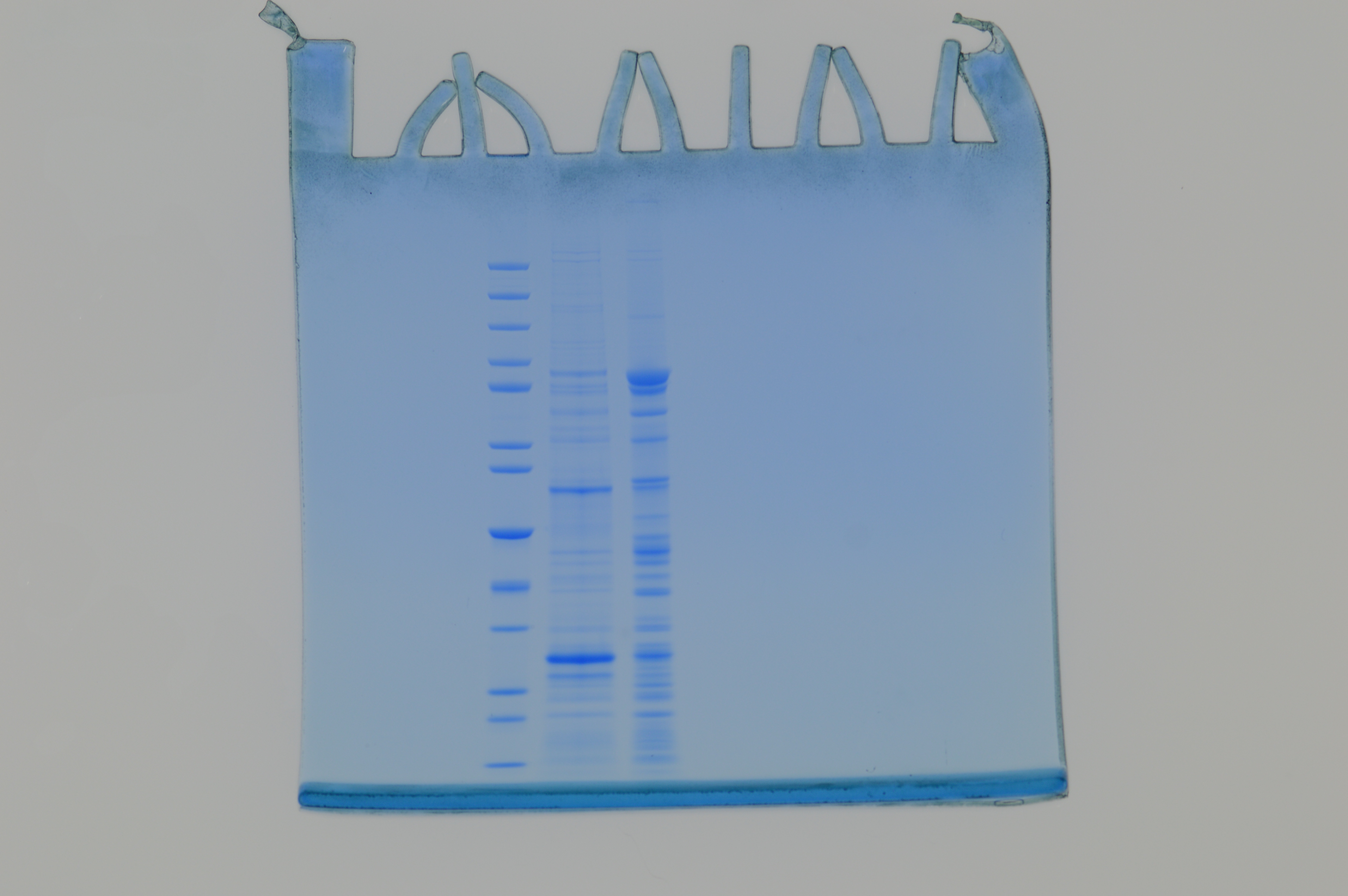

Supplement: Supplementary file 5 — Source Data for Figure 2 [file EMBR-24-e57984-s004.zip › Soruce Data Figure 2/2A/Source Data Fig 2A SDS_2_right_panel_Rsa4_Las1.JPG]

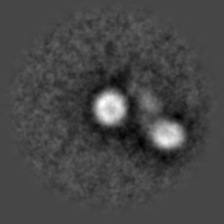

Supplement: Supplementary file 5 — Source Data for Figure 2 [file EMBR-24-e57984-s004.zip › Soruce Data Figure 2/ 2D/Source Data Fig. 2D individual 2D classes/50007.tif]

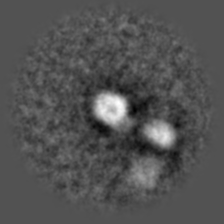

Supplement: Supplementary file 5 — Source Data for Figure 2 [file EMBR-24-e57984-s004.zip › Soruce Data Figure 2/ 2D/Source Data Fig. 2D individual 2D classes/50013.tif]

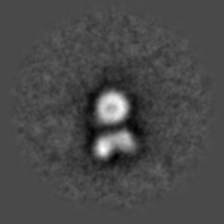

Supplement: Supplementary file 5 — Source Data for Figure 2 [file EMBR-24-e57984-s004.zip › Soruce Data Figure 2/ 2D/Source Data Fig. 2D individual 2D classes/50012.tif]

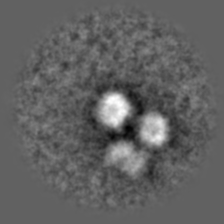

Supplement: Supplementary file 5 — Source Data for Figure 2 [file EMBR-24-e57984-s004.zip › Soruce Data Figure 2/ 2D/Source Data Fig. 2D individual 2D classes/50006.tif]

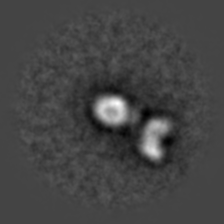

Supplement: Supplementary file 5 — Source Data for Figure 2 [file EMBR-24-e57984-s004.zip › Soruce Data Figure 2/ 2D/Source Data Fig. 2D individual 2D classes/50038.tif]

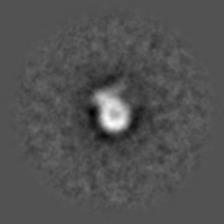

Supplement: Supplementary file 5 — Source Data for Figure 2 [file EMBR-24-e57984-s004.zip › Soruce Data Figure 2/ 2D/Source Data Fig. 2D individual 2D classes/50010.tif]

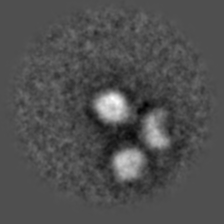

Supplement: Supplementary file 5 — Source Data for Figure 2 [file EMBR-24-e57984-s004.zip › Soruce Data Figure 2/ 2D/Source Data Fig. 2D individual 2D classes/50004.tif]

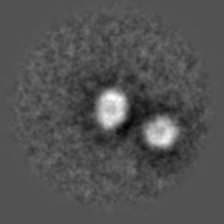

Supplement: Supplementary file 5 — Source Data for Figure 2 [file EMBR-24-e57984-s004.zip › Soruce Data Figure 2/ 2D/Source Data Fig. 2D individual 2D classes/50005.tif]

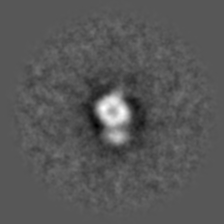

Supplement: Supplementary file 5 — Source Data for Figure 2 [file EMBR-24-e57984-s004.zip › Soruce Data Figure 2/ 2D/Source Data Fig. 2D individual 2D classes/50011.tif]

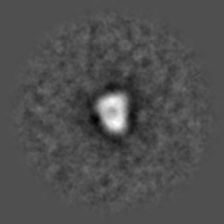

Supplement: Supplementary file 5 — Source Data for Figure 2 [file EMBR-24-e57984-s004.zip › Soruce Data Figure 2/ 2D/Source Data Fig. 2D individual 2D classes/50039.tif]

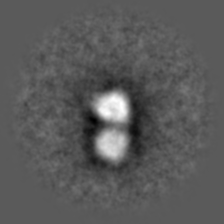

Supplement: Supplementary file 5 — Source Data for Figure 2 [file EMBR-24-e57984-s004.zip › Soruce Data Figure 2/ 2D/Source Data Fig. 2D individual 2D classes/50015.tif]

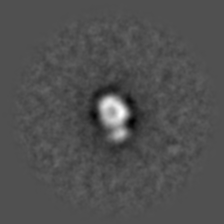

Supplement: Supplementary file 5 — Source Data for Figure 2 [file EMBR-24-e57984-s004.zip › Soruce Data Figure 2/ 2D/Source Data Fig. 2D individual 2D classes/50001.tif]

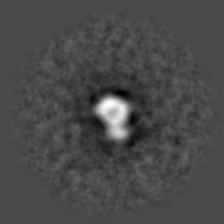

Supplement: Supplementary file 5 — Source Data for Figure 2 [file EMBR-24-e57984-s004.zip › Soruce Data Figure 2/ 2D/Source Data Fig. 2D individual 2D classes/50029.tif]

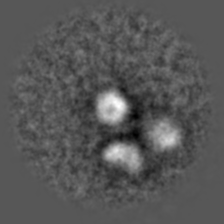

Supplement: Supplementary file 5 — Source Data for Figure 2 [file EMBR-24-e57984-s004.zip › Soruce Data Figure 2/ 2D/Source Data Fig. 2D individual 2D classes/50028.tif]

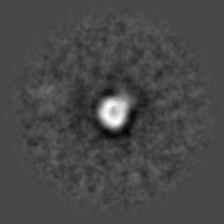

Supplement: Supplementary file 5 — Source Data for Figure 2 [file EMBR-24-e57984-s004.zip › Soruce Data Figure 2/ 2D/Source Data Fig. 2D individual 2D classes/50014.tif]

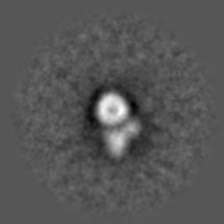

Supplement: Supplementary file 5 — Source Data for Figure 2 [file EMBR-24-e57984-s004.zip › Soruce Data Figure 2/ 2D/Source Data Fig. 2D individual 2D classes/50002.tif]

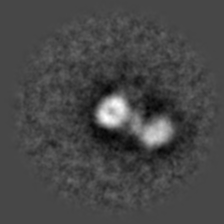

Supplement: Supplementary file 5 — Source Data for Figure 2 [file EMBR-24-e57984-s004.zip › Soruce Data Figure 2/ 2D/Source Data Fig. 2D individual 2D classes/50016.tif]

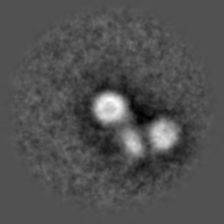

Supplement: Supplementary file 5 — Source Data for Figure 2 [file EMBR-24-e57984-s004.zip › Soruce Data Figure 2/ 2D/Source Data Fig. 2D individual 2D classes/50017.tif]

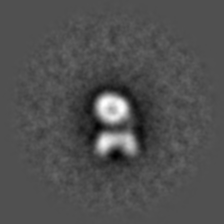

Supplement: Supplementary file 5 — Source Data for Figure 2 [file EMBR-24-e57984-s004.zip › Soruce Data Figure 2/ 2D/Source Data Fig. 2D individual 2D classes/50003.tif]

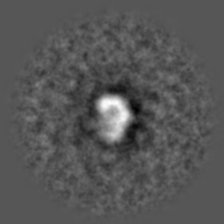

Supplement: Supplementary file 5 — Source Data for Figure 2 [file EMBR-24-e57984-s004.zip › Soruce Data Figure 2/ 2D/Source Data Fig. 2D individual 2D classes/50049.tif]

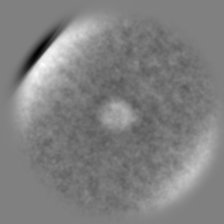

Supplement: Supplementary file 5 — Source Data for Figure 2 [file EMBR-24-e57984-s004.zip › Soruce Data Figure 2/ 2D/Source Data Fig. 2D individual 2D classes/50048.tif]

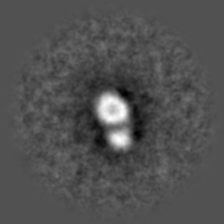

Supplement: Supplementary file 5 — Source Data for Figure 2 [file EMBR-24-e57984-s004.zip › Soruce Data Figure 2/ 2D/Source Data Fig. 2D individual 2D classes/50045.tif]

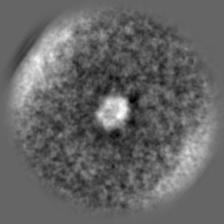

Supplement: Supplementary file 5 — Source Data for Figure 2 [file EMBR-24-e57984-s004.zip › Soruce Data Figure 2/ 2D/Source Data Fig. 2D individual 2D classes/50050.tif]

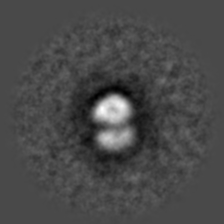

Supplement: Supplementary file 5 — Source Data for Figure 2 [file EMBR-24-e57984-s004.zip › Soruce Data Figure 2/ 2D/Source Data Fig. 2D individual 2D classes/50044.tif]

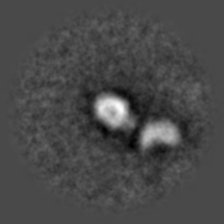

Supplement: Supplementary file 5 — Source Data for Figure 2 [file EMBR-24-e57984-s004.zip › Soruce Data Figure 2/ 2D/Source Data Fig. 2D individual 2D classes/50046.tif]

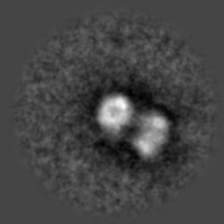

Supplement: Supplementary file 5 — Source Data for Figure 2 [file EMBR-24-e57984-s004.zip › Soruce Data Figure 2/ 2D/Source Data Fig. 2D individual 2D classes/50047.tif]

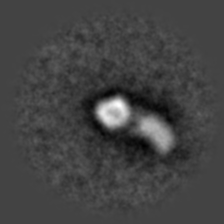

Supplement: Supplementary file 5 — Source Data for Figure 2 [file EMBR-24-e57984-s004.zip › Soruce Data Figure 2/ 2D/Source Data Fig. 2D individual 2D classes/50043.tif]

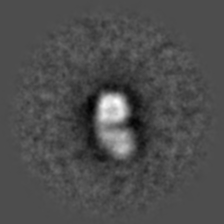

Supplement: Supplementary file 5 — Source Data for Figure 2 [file EMBR-24-e57984-s004.zip › Soruce Data Figure 2/ 2D/Source Data Fig. 2D individual 2D classes/50042.tif]

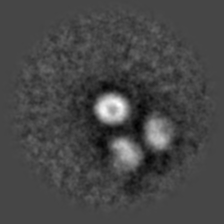

Supplement: Supplementary file 5 — Source Data for Figure 2 [file EMBR-24-e57984-s004.zip › Soruce Data Figure 2/ 2D/Source Data Fig. 2D individual 2D classes/50040.tif]

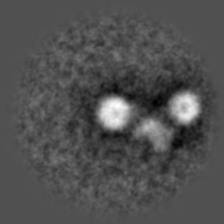

Supplement: Supplementary file 5 — Source Data for Figure 2 [file EMBR-24-e57984-s004.zip › Soruce Data Figure 2/ 2D/Source Data Fig. 2D individual 2D classes/50041.tif]

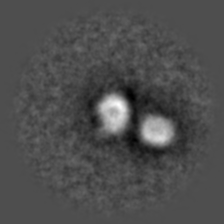

Supplement: Supplementary file 5 — Source Data for Figure 2 [file EMBR-24-e57984-s004.zip › Soruce Data Figure 2/ 2D/Source Data Fig. 2D individual 2D classes/50026.tif]

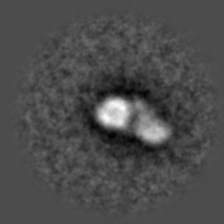

Supplement: Supplementary file 5 — Source Data for Figure 2 [file EMBR-24-e57984-s004.zip › Soruce Data Figure 2/ 2D/Source Data Fig. 2D individual 2D classes/50032.tif]

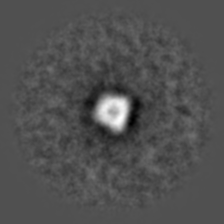

Supplement: Supplementary file 5 — Source Data for Figure 2 [file EMBR-24-e57984-s004.zip › Soruce Data Figure 2/ 2D/Source Data Fig. 2D individual 2D classes/50033.tif]

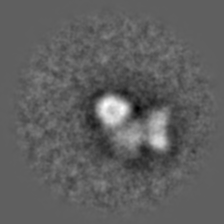

Supplement: Supplementary file 5 — Source Data for Figure 2 [file EMBR-24-e57984-s004.zip › Soruce Data Figure 2/ 2D/Source Data Fig. 2D individual 2D classes/50027.tif]

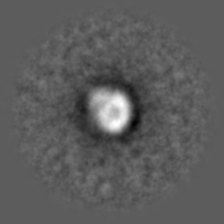

Supplement: Supplementary file 5 — Source Data for Figure 2 [file EMBR-24-e57984-s004.zip › Soruce Data Figure 2/ 2D/Source Data Fig. 2D individual 2D classes/50019.tif]

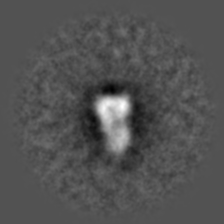

Supplement: Supplementary file 5 — Source Data for Figure 2 [file EMBR-24-e57984-s004.zip › Soruce Data Figure 2/ 2D/Source Data Fig. 2D individual 2D classes/50031.tif]

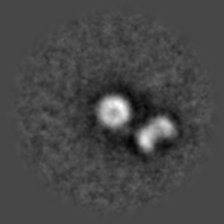

Supplement: Supplementary file 5 — Source Data for Figure 2 [file EMBR-24-e57984-s004.zip › Soruce Data Figure 2/ 2D/Source Data Fig. 2D individual 2D classes/50025.tif]

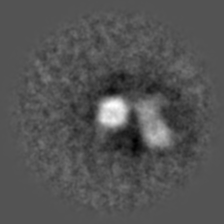

Supplement: Supplementary file 5 — Source Data for Figure 2 [file EMBR-24-e57984-s004.zip › Soruce Data Figure 2/ 2D/Source Data Fig. 2D individual 2D classes/50024.tif]

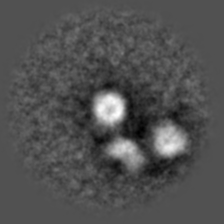

Supplement: Supplementary file 5 — Source Data for Figure 2 [file EMBR-24-e57984-s004.zip › Soruce Data Figure 2/ 2D/Source Data Fig. 2D individual 2D classes/50030.tif]

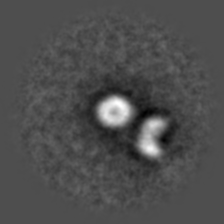

Supplement: Supplementary file 5 — Source Data for Figure 2 [file EMBR-24-e57984-s004.zip › Soruce Data Figure 2/ 2D/Source Data Fig. 2D individual 2D classes/50018.tif]

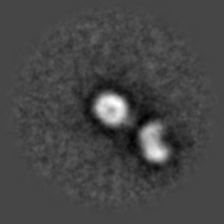

Supplement: Supplementary file 5 — Source Data for Figure 2 [file EMBR-24-e57984-s004.zip › Soruce Data Figure 2/ 2D/Source Data Fig. 2D individual 2D classes/50034.tif]

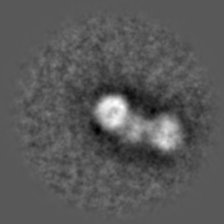

Supplement: Supplementary file 5 — Source Data for Figure 2 [file EMBR-24-e57984-s004.zip › Soruce Data Figure 2/ 2D/Source Data Fig. 2D individual 2D classes/50020.tif]

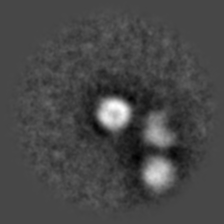

Supplement: Supplementary file 5 — Source Data for Figure 2 [file EMBR-24-e57984-s004.zip › Soruce Data Figure 2/ 2D/Source Data Fig. 2D individual 2D classes/50008.tif]

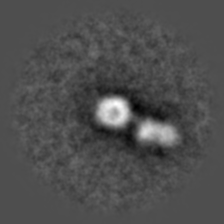

Supplement: Supplementary file 5 — Source Data for Figure 2 [file EMBR-24-e57984-s004.zip › Soruce Data Figure 2/ 2D/Source Data Fig. 2D individual 2D classes/50009.tif]

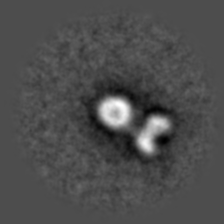

Supplement: Supplementary file 5 — Source Data for Figure 2 [file EMBR-24-e57984-s004.zip › Soruce Data Figure 2/ 2D/Source Data Fig. 2D individual 2D classes/50021.tif]

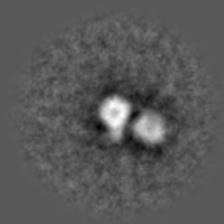

Supplement: Supplementary file 5 — Source Data for Figure 2 [file EMBR-24-e57984-s004.zip › Soruce Data Figure 2/ 2D/Source Data Fig. 2D individual 2D classes/50035.tif]

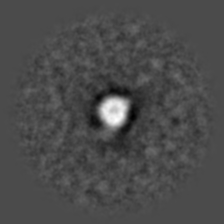

Supplement: Supplementary file 5 — Source Data for Figure 2 [file EMBR-24-e57984-s004.zip › Soruce Data Figure 2/ 2D/Source Data Fig. 2D individual 2D classes/50023.tif]

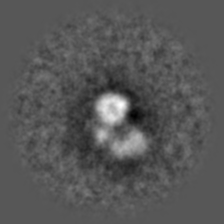

Supplement: Supplementary file 5 — Source Data for Figure 2 [file EMBR-24-e57984-s004.zip › Soruce Data Figure 2/ 2D/Source Data Fig. 2D individual 2D classes/50037.tif]

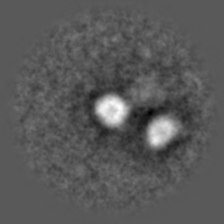

Supplement: Supplementary file 5 — Source Data for Figure 2 [file EMBR-24-e57984-s004.zip › Soruce Data Figure 2/ 2D/Source Data Fig. 2D individual 2D classes/50036.tif]

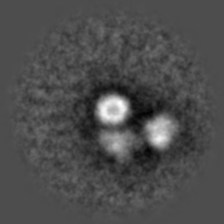

Supplement: Supplementary file 5 — Source Data for Figure 2 [file EMBR-24-e57984-s004.zip › Soruce Data Figure 2/ 2D/Source Data Fig. 2D individual 2D classes/50022.tif]

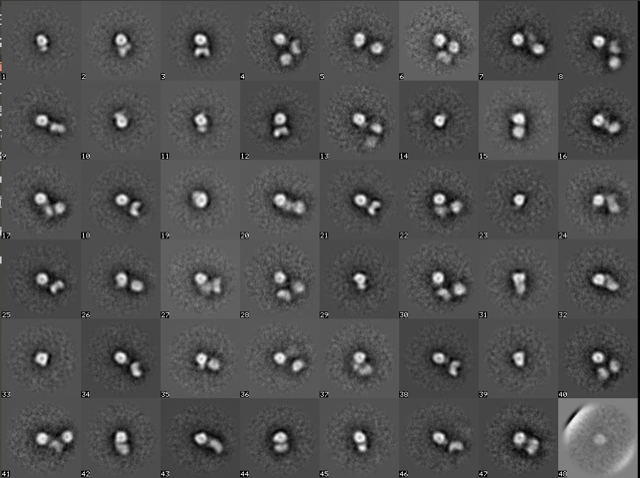

Supplement: Supplementary file 5 — Source Data for Figure 2 [file EMBR-24-e57984-s004.zip › Soruce Data Figure 2/ 2D/Source Data Fig. 2D all 2D classes.tif]

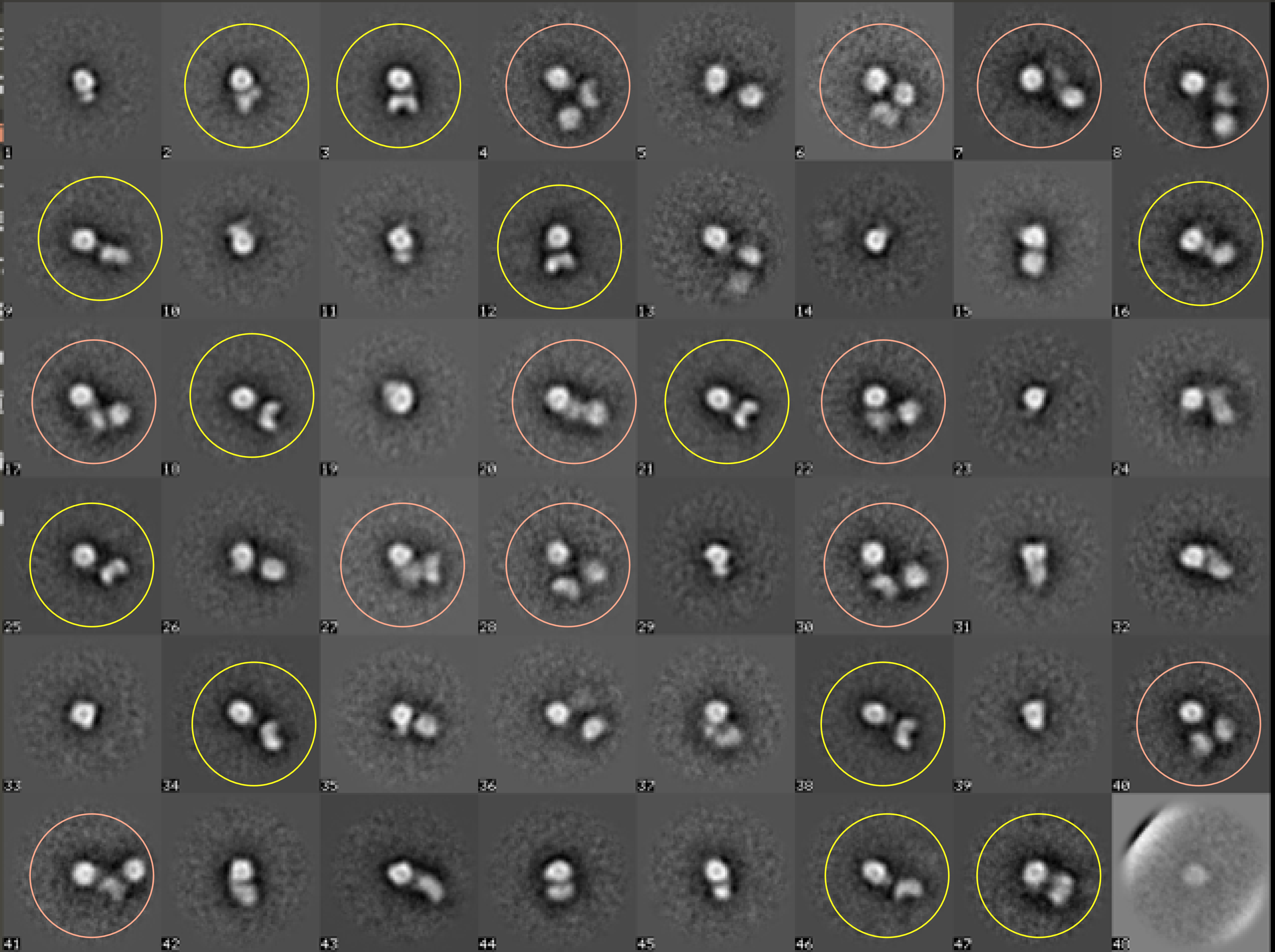

Supplement: Supplementary file 5 — Source Data for Figure 2 [file EMBR-24-e57984-s004.zip › Soruce Data Figure 2/ 2D/Source Data Fig. 2D picked classes labeled.tif]

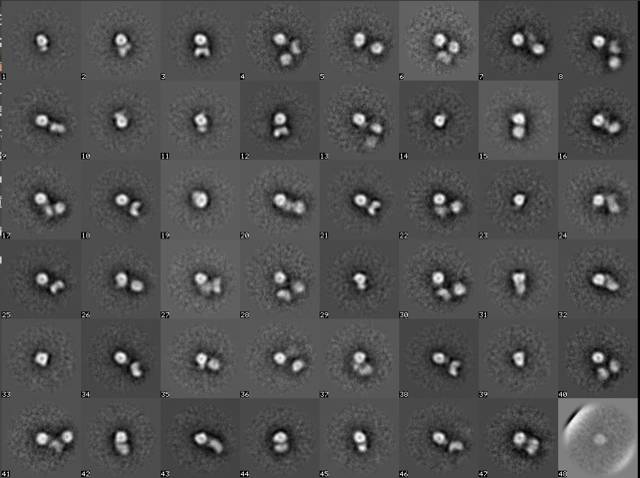

Supplement: Supplementary file 5 — Source Data for Figure 2 [file EMBR-24-e57984-s004.zip › Soruce Data Figure 2/ 2D/Source Data Fig. 2D all 2D classes.png]

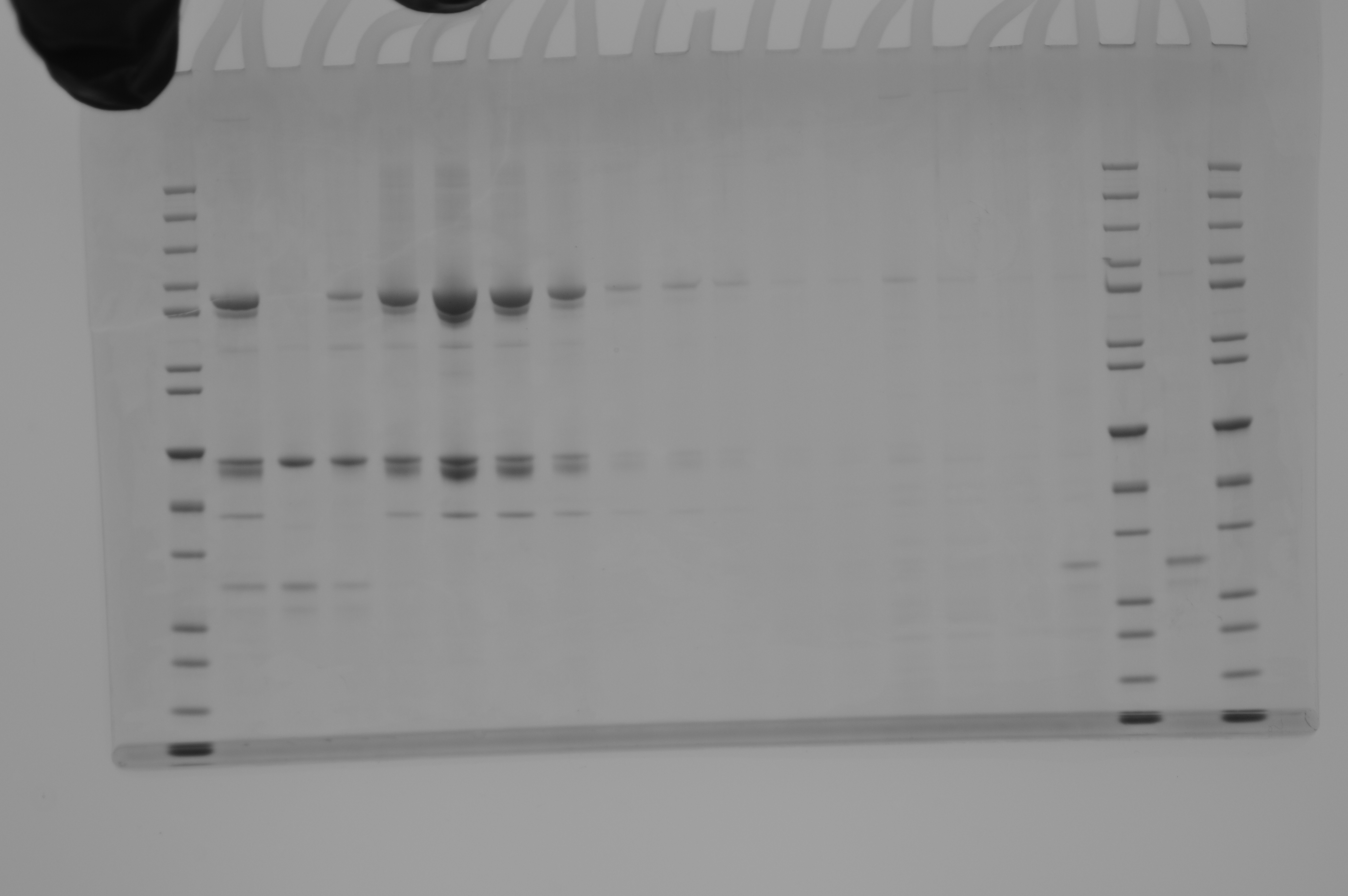

Supplement: Supplementary file 5 — Source Data for Figure 2 [file EMBR-24-e57984-s004.zip › Soruce Data Figure 2/2C/ Source Data Fig 2C SDS_PTF-Las1_sucrose.JPG]

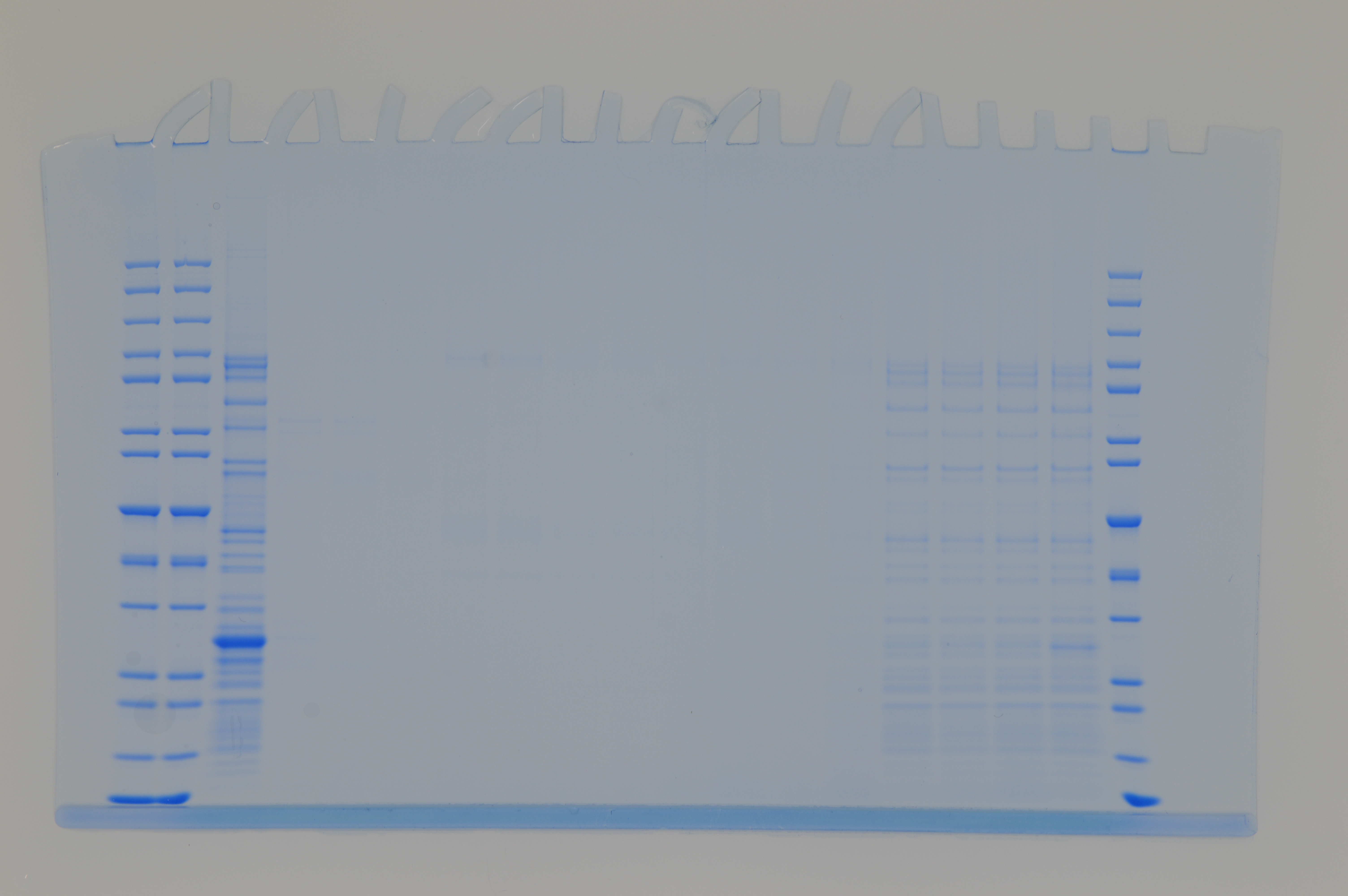

Supplement: Supplementary file 5 — Source Data for Figure 2 [file EMBR-24-e57984-s004.zip › Soruce Data Figure 2/2B/Source Data Fig 2B SDS_Rsa4_Rix1 sucrose.JPG]
