# Supplementary material for: Structural insights into coordinating 5S RNP rotation with ITS2 pre‐RNA processing during ribosome formation
Source: EMBO Rep. 2023 Nov 3;24(12):e57984. doi: 10.15252/embr.202357984 (PMC10702828; doi:10.15252/embr.202357984)
Supplement: Supplementary file 6 [file EMBR-24-e57984-s001.pdf]

Coordinating 5S RNP Rotation with ITS2 pre-RNA Processing During Ribosome Formation

Matthias Thoms, Benjamin Lau, Jingdong Cheng, Lisa Fromm, Timo Denk, Nikola Kellner, Dirk Flemming, Paulina Fischer, Laurent Falquet, Otto Berninghausen, Roland Beckmann, and Ed Hurt
DOI: 10.15252/embr.202357984

Corresponding author(s): Ed Hurt (ed.hurt@bzh.uni-heidelberg.de) , Ed Hurt (ed.hurt@bzh.uni-heidelberg.de), Roland Beckmann (beckmann@genzentrum.lmu.de)

Review Timeline:

Submission Date:	13th Aug 23
Editorial Decision:	13th Sep 23
Revision Received:	29th Sep 23
Editorial Decision:	9th Oct 23
Revision Received:	11th Oct 23
Accepted:	14th Oct 23

Editor: Esther Schnapp

Transaction Report:

(Note: With the exception of the correction of typographical or spelling errors that could be a source of ambiguity, letters and reports are not edited. Depending on transfer agreements, referee reports obtained elsewhere may or may not be included in this compilation. Referee reports are anonymous unless the Referee chooses to sign their reports.)

Dear Prof. Hurt,

Thank you for the submission of your manuscript to EMBO reports. We have now received the full set of referee reports that is pasted below.

As you will see, the referees acknowledge the quality of your work, and that the findings are interesting and a good fit for EMBO reports. We also think that your paper fits our criteria for "first confirmation studies" very well. The referees have only rather minor comments, and I would like to know whether you can address all within 2 weeks? If we can receive the revised ms before the 1st of October, we should be able to include it in our December issue, which would be better than moving it to the January issue, given the recently published, overlapping paper.

Please let me know in case this does not work for you.

Please also carefully address all requests listed below, as otherwise more rounds of revisions will be required.

I would thus like to invite you to revise your manuscript with the understanding that the referee concerns must be fully addressed and their suggestions taken on board. Please address all referee concerns in a complete point-by-point response. Acceptance of the manuscript will depend on a positive outcome of a second round of review. It is EMBO reports policy to allow a single round of major revision only and acceptance or rejection of the manuscript will therefore depend on the completeness of your responses included in the next, final version of the manuscript.

IMPORTANT NOTE: we perform an initial quality control of all revised manuscripts before re-review. Your manuscript will FAIL this control and the handling will be DELAYED if the following APPLIES:
1) A data availability section providing access to data deposited in public databases is missing. If you have not deposited any data, please add a sentence to the data availability section that explains that.
2) Your manuscript contains statistics and error bars based on n=2. Please use scatter blots in these cases. No statistics should be calculated if n=2.

When submitting your revised manuscript, please carefully review the instructions that follow below. Failure to include requested items will delay the evaluation of your revision.

1) a .docx formatted version of the manuscript text (including legends for main figures, EV figures and tables). Please make sure that the changes are highlighted to be clearly visible.

2) individual production quality figure files as .eps, .tif, .jpg (one file per figure). See https://wol-prod-cdn.literatumonline.com/pb-assets/embo-site/EMBOPress_Figure_Guidelines_061115-1561436025777.pdf for more info on how to prepare your figures.

3) We replaced Supplementary Information with Expanded View (EV) Figures and Tables that are collapsible/expandable online. A maximum of 5 EV Figures can be typeset. EV Figures should be cited as 'Figure EV1, Figure EV2" etc... in the text and their respective legends should be included in the main text after the legends of regular figures.

- For the figures that you do NOT wish to display as Expanded View figures, they should be bundled together with their legends in a single PDF file called *Appendix*, which should start with a short Table of Content. Appendix figures should be referred to in the main text as: "Appendix Figure S1, Appendix Figure S2" etc. See detailed instructions regarding expanded view here: <https://www.embopress.org/page/journal/14693178/authorguide#expandedview>

- Additional Tables/Datasets should be labeled and referred to as Table EV1, Dataset EV1, etc. Legends have to be provided in a separate tab in case of .xls files. Alternatively, the legend can be supplied as a separate text file (README) and zipped together with the Table/Dataset file.

4) a .docx formatted letter INCLUDING the reviewers' reports and your detailed point-by-point responses to their comments. As part of the EMBO Press transparent editorial process, the point-by-point response is part of the Review Process File (RPF), which will be published alongside your paper.

5) a complete author checklist, which you can download from our author guidelines <https://www.embopress.org/page/journal/14693178/authorguide>. Please insert information in the checklist that is also reflected in the manuscript. The completed author checklist will also be part of the RPF.

6) Please note that all corresponding authors are required to supply an ORCID ID for their name upon submission of a revised manuscript (<https://orcid.org/>). Please find instructions on how to link your ORCID ID to your account in our manuscript tracking system in our Author guidelines
<https://www.embopress.org/page/journal/14693178/authorguide#authorshipguidelines>

7) Before submitting your revision, primary datasets produced in this study need to be deposited in an appropriate public database (see https://www.embopress.org/page/journal/14693178/authorguide#datadeposition). Please remember to provide a reviewer password if the datasets are not yet public. The accession numbers and database should be listed in a formal "Data Availability" section placed after Materials & Method (see also https://www.embopress.org/page/journal/14693178/authorguide#datadeposition). Please note that the Data Availability Section is restricted to new primary data that are part of this study. * Note - All links should resolve to a page where the data can be accessed. *
If your study has not produced novel datasets, please mention this fact in the Data Availability Section.

8) At EMBO Press we ask authors to provide source data for the main manuscript figures. Our source data coordinator will contact you to discuss which figure panels we would need source data for and will also provide you with helpful tips on how to upload and organize the files.

9) Our journal also encourages inclusion of *data citations in the reference list* to directly cite datasets that were re-used and obtained from public databases. Data citations in the article text are distinct from normal bibliographical citations and should directly link to the database records from which the data can be accessed. In the main text, data citations are formatted as follows: "Data ref: Smith et al, 2001" or "Data ref: NCBI Sequence Read Archive PRJNA342805, 2017". In the Reference list, data citations must be labeled with "[DATASET]". A data reference must provide the database name, accession number/identifiers and a resolvable link to the landing page from which the data can be accessed at the end of the reference. Further instructions are available at https://www.embopress.org/page/journal/14693178/authorguide#referencesformat

10) Regarding data quantification (see Figure Legends: https://www.embopress.org/page/journal/14693178/authorguide#figureformat)

The following points must be specified in each figure legend:

- the name of the statistical test used to generate error bars and P values,

- the number (n) of independent experiments (please specify technical or biological replicates) underlying each data point,

- the nature of the bars and error bars (s.d., s.e.m.),

- If the data are obtained from n = 2, use scatter blots showing the individual data points.

Discussion of statistical methodology can be reported in the materials and methods section, but figure legends should contain a basic description of n, P and the test applied.

- Please also include scale bars in all microscopy images.

11) The journal requires a statement specifying whether or not authors have competing interests (defined as all potential or actual interests that could be perceived to influence the presentation or interpretation of an article). In case of competing interests, this must be specified in your disclosure statement. Further information: https://www.embopress.org/competing-interests

We would also welcome the submission of cover suggestions, or motifs to be used by our Graphics Illustrator in designing a cover.

As part of the EMBO publication's Transparent Editorial Process, EMBO reports publishes online a Review Process File (RPF) to accompany accepted manuscripts. This File will be published in conjunction with your paper and will include the referee reports, your point-by-point response and all pertinent correspondence relating to the manuscript.

You are able to opt out of this by letting the editorial office know (emboreports@embo.org). If you do opt out, the Review Process File link will point to the following statement: "No Review Process File is available with this article, as the authors have chosen not to make the review process public in this case."

I look forward to seeing a revised form of your manuscript when it is ready.

Best regards,
Esther

Esther Schnapp, PhD
Senior Editor

Referee #1:

The authors report excellent structural analyses of pre-60S particles accumulated in C. thermophilum expressing dominant negative protein ct Rsa4 E117D together with split tags. The work follows on from a recent EMBO Rep. publication that used ct Ytm1E88D in split bait purifications to characterize an earlier set of particles. The structural biology is excellent and the authors do a good job of relating the findings to previous studies in budding and fission yeasts and vert recent analyses in human cells.

Although the conclusions are not really groundbreaking, and are of somewhat specialist interest, the ribosome synthesis pathway is of huge importance and understanding the similarities and differences between organisms is very valuable. I very seldom recommend publication without changes, but am happy to do so in this case.

Referee #2:

Matthias Thoms and colleagues determined the cryo-EM structures of the rixosome-containing pre-60S particles, uncovering a possible role of rixosome in coordinating the 5S RNP rotation with ITS2 processing. Very importantly, this study presents a novel structural snapshot that is missing from published studies, and it provides insights into the mechanism of the initiation of the ITS2 processing and the 5S RNP rotation (to be more accurately, the CP rotation). Overall, the data is of high quality and the manuscript is well written.

A major concern is the interpretation of the structural data. As described in the title and abstract, the authors attributed a role of the Rix1 complex in the 5S RNP rotation. The authors have successfully captured the Rix1 complex in a state prior to the 5S rotation and observed that upon rotation, the Rix1 complex moves along with its contacts of CP components. The fact that the Rix1 complex wedges between the 5S rRNA and the L1 stalk does not necessarily mean that it would "force the immature 5S RNP to rotate". Based on previous structural data, including two very recent studies on human pre-60S particles (Arnaud Vanden Broeck, Science, 2023; Zhang, Y., Cell Research, 2023), Rrs1 and Rpf2 could be the two factors and their departure is coupled with the rotation of the CP. In particular, Zhang et al. has discussed the conformational changes of H80 during the CP rotation. Therefore, the authors could examine 1) structural changes of Rrs1/Rpf2 contact sites on the pre-60S before and after the CP rotation; 2) conformational difference of H80 (two linker regions of H80-H88); 3) the long linker of Rpf2 that is inserted into the junctional region of the central helices. It would be interesting to know whether these regions could provide a triggering signal to initiate the CP rotation. The authors could present their analysis in the revision and should revise the title and the main text accordingly.

Minor issues:
(1) In the abstract, "Finally, Rix1 complex recruitment and inward movement of the L1 stalk allow the flexible Nop53 N-terminus with its AIM motif to be strategically positioned at the L1-stalk's base, facilitating Mtr4 helicase-exosome's role in completing ITS2 removal." The authors seem to assert that both the Rix1 complex's recruitment and the L1 stalk's inward movement are crucial for Mtr4-exosome's recruitment and the subsequent ITS2 removal. Yet, Figure EV4 depicts state 5, where the N-terminal extension of Nop53 appears stabilized around the inward L1 stalk and the Rix1 complex is not involved. This suggests that the inward movement of the L1 stalk alone suffices for Mtr4's recruitment. This suggests that Sda1 might be more important in this action, as it stabilizes the L1 stalk in the closed conformation.

(2) Regarding several states displayed in Figure EV3, where the foot structure seems to be partially or entirely removed prior to the 5S RNP rotation. In these situations, how the GRC3-Las1 complex could be positioned to the pre-60S?

(3) A typographical error is present at the end of page 7: "Las-Grc3" should be corrected.

(4) A mixed use of both Nog2 and Nug2.

Referee #3:

In this article, M. Thoms and colleagues describe cryo-EM structures of both nucleolar and nuclear pre-60S particles, purified from Chaetomium thermophilum. Using an elegant purification scheme that combines split-tagging and the use of mutant pre-ribosomal factors that slows down nuclear maturation of pre-60S particles, the authors were able to obtain 3D structures of firstly the so-called rixosome complex, (namely, the butterfly-like Las1-Grc3 and the doughnut-shaped Rix1-Ipi1-Ip3 modules) which can be co-purified with pre-60S particles. The authors also present atomic-resolution structures of pre-60S particles containing the Rix1 submodule, and in these structures the 5S RNP is found either in a rotated or in a mature conformation. They propose

that the Rix1 submodule could serve as a hub for various operators of the ribosome assembly line, and thus coordinate two major events of nuclear maturation of 60S ribosomal subunits : cleavage of the ITS2 sequence from the 60S pre-rRNA (initiated by the Las1-Grc3 module of the Rixosome), and 5S RNP/core protuberance rotation into its mature state. Based on their cryo-EM observations, they propose that ITS2 cleavage might precede the rotation of the 5S core protuberance in its mature position, and that the AAA+ATPase Rea1 might not be the primary cause of 5S RNP rotation, as previously deduced from yeast results. Overall, the work provided by the authors is very solid (the cryo-EM processing schemes are true masterpieces), and provides structures of nucle(ol)ar pre-60S that were not obtained in C.thermophilum until now. More broadly, this structural work gives new insights on the nucleolar-to nuclear processing pathway of large ribosomal subunits of C.thermophilum, and confirm very recent results obtained in human cells (Vanden Broeck & Klinge, Science 2023). I thus believe these results are worth publishing in EMBO reports.

However, to follow the embo reports guidelines for referees, the following points should be revised before final publication of this manuscript :
- « how to do the study justice without overselling the claims »:
Figure 1A : In the beginning of the results section, the authors performed coIPs using either a wt or mutant (E117D) version of Rsa4 as bait, and claim that mutant Rsa4 affinity purification « showed co-
enrichment of a number of typical pre-60S factors known from yeast, but the wild-type Rsa4 eluate was largely devoid of these components, but instead was rather associated with a few heat shock proteins, Rea1 and ribosomes ». This should be explained with slightly more details : how can the authors know that it's mature ribosomes, and not pre-ribosomes? Do the authors mean that co-IP yields mostly RPLs, and not the pre-60S factors that are found in yeast or with mutant ctRsa4? Or do they mean that Rsa4 can be found associated to mature, translation-competent ribosomes in Chaetomium thermophilum ?

Figure 2B, the authors have performed sucrose gradient analysis of their pre-60S eluates (ct Rsa4 E117D Rix1-Flag eluate), and claim that this experiment « showed that Rix1 complex and Las1-Grc3 strictly co-sedimented with the pre-60S particles, suggesting a tight physical interaction between
both [Rix1 and Las1-Grc3]sub-modules as well as the pre-60S subunit». This sentence is confusing, since there are bands with molecular weights corresponding to Grc3, Rix1, Las1, Ipi3 and Ip1 in lighter gradient fractions 4 and 5, which indicate a non-negligible level of dissociation between pre-60S particles and Rixosome complexes. A western blot analysis of each fractions might be more convincing to prove that at least the majority of purified Rixosome particles are bound to pre-60S particles. Otherwise the conclusion that « Rix1 complex and Las1-Grc3 strictly co-sedimented with the pre-60S particles » cannot be drawn.

- «how to represent earlier litterature more fairly »:
The article from Vanden Broeck & Klinge (Science 2023), which present similar structures of human pre-60S nucle(ol)ar particles with the Rix1 submodule as well as a short peptide of Las1 is not cited once in the manuscript. The findings in the current manuscript are in good agreement with what was published by the Klinge's lab, but performed in a different species, and the authors of the current manuscript have found good selling points of their story that clearly distinguish it from the Science article, so the latter should be at least mentioned in the discussion, all the more since the manuscript was classified as "first confirmation [of a study] by an independent group" by the editors.

- «how the clarity of the writing could be improved» :

In the results sections, the authors present 2D classes of Rixosome particles observed after negative staining, and write the following : « Since the Rix1-Ipi3-Ipi1 complex is known to exhibit a large hollow sphere with a diameter of ~9 nm in yeast (Barrio-Garcia et al., 2016; Kater et al., 2020) and
the reconstituted ct Las1-Grc3 heterodimer a butterfly-like structure of 7 nm x 13 nm (Pillon et al., 2017), we conclude that we can observe the entire Rixosome by electron microscopy ». The dimensions of the 2D classes should be clearly depicted in the text and/or figure so that this conclusion can fully and unambiguously convince readers.

Cryo-EM processing. The loose (flexible) connection between both Rixosome subcomplexes prevents reaching high resolution for the 3D structures of the full Rixosome particles. Thus, the authors processed the submodules separately, to reach 2.9 - 3.0 A resolution for the Rix1 and Las1-Grc3 subcomplexes, respectively. Could the authors precise whether they find, in their cryo-EM images or in their 2D classes, evidences of the existence of independent submodules (Rix1 alone or Las1-Grc3 alone), and how much of full rixosome vs. isolated submodules they can count ? Or, did they had to systematically mask out one submodule from full rixosome particles to study the other ? This is not entirely clear from the M&M or the processing schemes, and might help to understand the dynamics of interatction between Las1-Grc3 and Rix1 complex.

It is not entirely clear how strong the hypothesis regarding the timing of ITS2 maturation compared to 5S RNP rotation is for the authors. On the results part, based on their observations they draw the conclusion that the ITS2 cleavage precedes 5S RNP rotation («Altogether, we conclude that initial ITS2 processing by the Las1-Grc3 rixosome subcomplex likely precedes 5S RNP rotation »). However, and very understandably so, they don't provide direct evidence that ITS2 cleavage and trimming has been initiated by Las1-Grc3. Then, in the discussion part, this assessment is presented as a clear but not entirely proven hypothesis, which is a fairer way to present it : it might be worth reformulating the sentence of the results part as an hypothesis, not an unquestionable conclusion.

Minor point : please harmonize writing for the Rsa4 mutations (E117D, E>117D...)

Our point-by-point responses to the reviewers' comments (in red)

Referee #1:

The authors report excellent structural analyses of pre-60S particles accumulated in C. thermophilum expressing dominant negative protein ct Rsa4 E117D together with split tags. The work follows on from a recent EMBO Rep. publication that used ct Ytm1E88D in split bait purifications to characterize an earlier set of particles. The structural biology is excellent and the authors do a good job of relating the findings to previous studies in budding and fission yeasts and vert recent analyses in human cells.

Although the conclusions are not really groundbreaking, and are of somewhat specialist interest, the ribosome synthesis pathway is of huge importance and understanding the similarities and differences between organisms is very valuable. I very seldom recommend publication without changes, but am happy to do so in this case.

Thank you so much for the positive comments.

Referee #2:

Matthias Thoms and colleagues determined the cryo-EM structures of the rixosome-containing pre-60S particles, uncovering a possible role of rixosome in coordinating the 5S RNP rotation with ITS2 processing. Very importantly, this study presents a novel structural snapshot that is missing from published studies, and it provides insights into the mechanism of the initiation of the ITS2 processing and the 5S RNP rotation (to be more accurately, the CP rotation). Overall, the data is of high quality and the manuscript is well written.

A major concern is the interpretation of the structural data. As described in the title and abstract, the authors attributed a role of the Rix1 complex in the 5S RNP rotation. The authors have successfully captured the Rix1 complex in a state prior to the 5S rotation and observed that upon rotation, the Rix1 complex moves along with its contacts of CP components. The fact that the Rix1 complex wedges between the 5S rRNA and the L1 stalk does not necessarily mean that it would "force the immature 5S RNP to rotate". Based on previous structural data, including two very recent studies on human pre-60S particles (Arnaud Vanden Broeck, Science, 2023; Zhang, Y et al., Cell Research, 2023), Rrs1 and Rpf2 could be the two factors and their departure is coupled with the rotation of the CP. In particular, Zhang et al. has discussed the conformational changes of H80 during the CP rotation.

Therefore, the authors could examine 1) structural changes of Rrs1/Rpf2 contact sites on the pre-60S before and after the CP rotation;

It is well established that the Rrs1/Rpf2 heterodimer dissociates upon rotation of the 5S RNP/CP. As recently observed for the human system by Vanden Broeck, A. & Klinge, S., Science, 2023 and Zhang, Y. et al., Cell Res., 2023 and for the yeast system by us and others (e.g. Kater, L. et al., Mol Cell, 2020). Also in our maps we did not find any densities for Rpf2/Rrs1 after CP rotation indicating there complete dissociation.
Due to the lack of Rpf2-Rrs1 in the post 5S rotation state examination of the structural changes of Rrs1/Rpf2 contact sites on the pre-60S before and after the CP rotation, as suggested by the reviewer, is unfortunately not possible.

2) conformational difference of H80 (two linker regions of H80-H88);

As observed before by several labs in yeast and human the conformation of rRNA helix H80 as part of the CP is drastically different when comparing Pre-5S and Post-5S rotation states. Yet, there were no observable conformational differences of H80-H88 between the Pre-5S rotation state (State 1) without Sda1/Rix1 complex and the Pre-5S rotation state with engaged Sda1/Rix1 complex (State 2). We included the following sentence in the revised manuscript “However, Rpf2-Rrs1 as well as the connecting rRNA helices H80-88 of the immature CP show no structural differences between the pre-5S rotation states with and without engaged Sda1 and Rix1 complex.”

3) the long linker of Rpf2 that is inserted into the junctional region of the central helices.

We closely inspected the terminal extensions of Rrs1 and Rpf2 when bound to the pre-60S in the last states preceding rotation (+/- Sda1/Rix complex) without finding any differences in their interaction with the junctional region of the central helices. See also response to point 2)

It would be interesting to know whether these regions could provide a triggering signal to initiate the CP rotation. The authors could present their analysis in the revision and should revise the title and the main text accordingly.

We have analysed these points and did not find any indication for how these regions could provide a triggering signal to initiate CP rotation. As mentioned above our analysis revealed that the rRNA H80 and also the two proteins Rrs1 and Rpf2 remain in the exact same conformation in the Pre-5S rotation states before and after Sda1 and Rix1 complex binding. The only observed difference is that the binding of Sda1 together with the Rix1 complex eventually results in a small bent of the 5S rRNA tip in the direction of the rotation, supporting our hypothesis that this steric clash may serve as the trigger point for rotation. Therefore, Rrs1 and Rpf2 are likely acting in the stabilization of the un-rotated conformation of the 5S RNP and are completely dislodged (and dissociated) upon 5S RNP rotation.
Nevertheless, we revised the title and the main text accordingly in order to keep it more general. The new title 'Structural Insights into Coordinating 5S RNP Rotation with ITS2 pre-RNA Processing During Ribosome Formation' is less specific and hence addresses not only the role of the rixosome but also of other factors such as Rpf2 and Rrs1, which as pointed out by this reviewer are potentially involved in controlling 5S RNP rotation.

Minor issues:
(1) In the abstract, "Finally, Rix1 complex recruitment and inward movement of the L1 stalk allow the flexible Nop53 N-terminus with its AIM motif to be strategically positioned at the L1-stalk's base, facilitating Mtr4 helicase-exosome's role in completing ITS2 removal." The authors seem to assert that both the Rix1 complex's recruitment and the L1 stalk's inward movement are crucial for Mtr4-exosome's recruitment and the subsequent ITS2 removal. Yet, Figure EV4 depicts state 5, where the N-terminal extension of Nop53 appears stabilized around the inward L1 stalk and the Rix1 complex is not involved. This suggests that the inward movement of the L1 stalk alone suffices for Mtr4's recruitment. This suggests that Sda1 might be more important in this action, as it stabilizes the L1 stalk in the closed conformation.

Since we found the L1 stalk in the inward position in the absence of Rix1 complex but also in the absence of Sda1 we tuned down the statement in the abstract accordingly. "Finally, inward movement of the L1 stalk permit the flexible Nop53 N-terminus with its AIM motif to become positioned at the base of the L1-stalk to facilitate Mtr4 helicase-exosome participation for completing ITS2 removal"

(2) Regarding several states displayed in Figure EV3, where the foot structure seems to be partially or entirely removed prior to the 5S RNP rotation. In these situations, how the GRC3-Las1 complex could be positioned to the pre-60S?

We were aware about these states and concluded that the activity of the Grc3-Las1 complex to trigger the ITS2 processing is not strictly dependent on the presence of the Rix1 complex (see discussion). Whether and how the Grc3-Las1 complex is recruited to and positioned on the pre-60S in this situation remains elusive.
In our study, we only observed the Rix1 complex bound to pre-60S subunits in ~ 9.2% of the particles for the PT-Rsa4-E117D Flag-Las1 dataset and ~ 13.2% of particles for the PT-Rsa4-E117D Rix1-Flag dataset (see Appendix Figure S4), even though Las1 and Rix1, as part of the rixosome, were used for split purification. This at least suggests, that the Rix1 complex/rixosome could dissociate during e.g. grid preparation or that the Rix1 complex might interact with the pre-60S particle in a highly flexible manner, not detectable by cryo-EM.
In the recent study by Vanden Broeck, A. and Klinge, S., Science, 2023 an interaction between the very C-terminus of LAS1L (Las1 homologue) and TEX10 (Ipi1 homologue) has been observed. This interaction is not possible in *C. thermophilum* or *S. cerevisae* as the interacting regions of TEX10 and LAS1L do not exist in these lower eukaryotes. Nevertheless, it is possible that similar small peptide interaction exist which are not yet discovered. One of these might be the interaction between the Las1-Grc3 complex/rixosome and the ITS2-Foot structure. Recent crosslink data identified an inter-protein crosslink between Grc3 (K475) and the Nsa3 C-terminus (K363) in *S. cerevisae* (Sailer, C. et al., Cell Reports, 2022). Nevertheless, the C-terminus of Nsa3 was not resolved in corresponding yeast and human structures and is therefore likely too flexible. In *C. thermophilum* Nsa3 is exchanged by its 90S paralog Utp30 and also here the C-terminus is not visible.

Taken together neither published nor our data provide a clear explanation for reliable positioning of Las1-Grc3 on the pre-60S subunits with or without Rix1 complex. Therefore, we do not want to speculate in the ms without further evidence.

(3) A typographical error is present at the end of page 7: "Las-Grc3" should be corrected.
The error has been corrected.
(4) A mixed use of both Nog2 and Nug2.
This has been corrected, we use now Nog2 in the revised version.

Referee #3:

In this article, M. Thoms and colleagues describe cryo-EM structures of both nucleolar and nuclear pre-60S particles, purified from Chaetomium thermophilum. Using an elegant purification scheme that combines split-tagging and the use of mutant pre-ribosomal factors that slows down nuclear maturation of pre-60S particles, the authors were able to obtain 3D structures of firstly the so-called rixosome complex, (namely, the butterfly-like Las1-Grc3 and the doughnut-shaped Rix1-Ipi1-Ip3 modules) which can be co-purified with pre-60S particles. The authors also present atomic-resolution structures of pre-60S particles containing the Rix1 submodule, and in these structures the 5S RNP is found either in a rotated or in a mature conformation. They propose that the Rix1 submodule could serve as a hub for various operators of the ribosome assembly line, and thus coordinate two major events of nuclear maturation of 60S ribosomal subunits : cleavage of the ITS2 sequence from the 60S pre-rRNA (initiated by the Las1-Grc3 module of the Rixosome), and 5S RNP/core protuberance rotation into its mature state. Based on their cryo-EM observations, they propose that ITS2 cleavage might precede the rotation of the 5S core protuberance in its mature position, and that the AAA+ATPase Rea1 might not be the primary cause of 5S RNP rotation, as previously deduced from yeast results. Overall, the work provided by the authors is very solid (the cryo-EM processing schemes are true masterpieces), and provides structures of nucle(ol)ar pre-60S that were not obtained in C.thermophilum until now. More broadly, this structural work gives new insights on the nucleolar-to nuclear processing pathway of large ribosomal subunits of C.thermophilum, and confirm very recent results obtained in human cells (Vanden Broeck & Klinge, Science 2023). I thus believe these results are worth publishing in EMBO reports.
However, to follow the embo reports guidelines for referees, the following points should be revised before final publication of this manuscript :
- « how to do the study justice without overselling the claims »:
Figure 1A : In the beginning of the results section, the authors performed coIPs using either a wt or mutant (E117D) version of Rsa4 as bait, and claim that mutant Rsa4 affinity purification « showed co-enrichment of a number of typical pre-60S factors known from yeast, but the wild-type Rsa4 eluate was largely devoid of these components, but instead was rather associated with a few heat shock proteins, Rea1 and ribosomes ». This should be explained with slightly more details : how can the authors know that it's mature ribosomes, and not pre-ribosomes? Do the authors mean that co-IP yields mostly RPLs, and not the pre-60S factors that are found in yeast or with mutant ctRsa4? Or do they mean that Rsa4 can be found associated to mature, translation-competent ribosomes in Chaetomium thermophilum?

This has been now clarified in the text. The reviewer is right in saying that we do not know whether these are mature ribosomes. We believe that it is a contamination of ribosomal proteins during affinity purification, which is frequently observed during affinity-purification of bait proteins. We have now described this in the text more clearly: "However, the wild-type Rsa4 co-purified its direct binding partner Rea1, but also heat shock proteins and ribosomal proteins, which are frequent contaminants during affinity-purifications (Fig 1A)".

Figure 2B, the authors have performed sucrose gradient analysis of their pre-60S eluates (ct Rsa4 E117D Rix1-Flag eluate), and claim that this experiment « showed that Rix1 complex and Las1-Grc3 strictly co-sedimented with the pre-60S particles, suggesting a tight physical interaction between both [Rix1 and Las1-Grc3] sub-modules as well as the pre-60S subunit». This sentence is confusing, since there are bands with molecular weights corresponding to Grc3, Rix1, Las1, Ipi3 and Ip1 in lighter gradient fractions 4 and 5, which indicate a non-negligible level of dissociation between pre-60S particles and Rixosome complexes. A western blot analysis of each fractions might be more convincing to prove that at least the majority of purified Rixosome particles are bound to pre-60S particles. Otherwise the conclusion that « Rix1 complex and Las1-Grc3 strictly co-sedimented with the pre-60S particles » cannot be drawn.

This has been revised in the text, by no longer using the terms 'strictly and tight' to leave it open whether a small pool also dissociates and remains on the top of the gradient: "Finally, the *ct* Rsa4 E117D Rix1-Flag eluate was analyzed by sucrose gradient centrifugation, which showed that the Rix1 complex and the Las1-Grc3 co-sedimented with the pre-60S particles, suggesting a physical interaction of both sub-modules with the pre-60S subunit (Fig 2B)." Unfortunately, we do not have antibodies against these *ct* factors to follow this by Western blot.

- «how to represent earlier literature more fairly »:
The article from Vanden Broeck & Klinge (Science 2023), which present similar structures of human pre-60S nucle(ol)ar particles with the Rix1 submodule as well as a short peptide of Las1 is not cited once in the manuscript. The findings in the current manuscript are in good agreement with what was published by the Klinge's lab, but performed in a different species, and the authors of the current manuscript have found good selling points of their story that clearly distinguish it from the Science article, so the latter should be at least mentioned in the discussion, all the more since the manuscript was classified as "first confirmation [of a study] by an independent group" by the editors.

The paper by Vanden Broeck & Klinge and the related publication by Zhang et al. (2023) dealing with human pre-60S particles are now cited in our revised manuscript and aspects overlapping with our findings are now mentioned in the discussion.

- «how the clarity of the writing could be improved» :
In the results sections, the authors present 2D classes of Rixosome particles observed after negative staining, and write the following : « Since the Rix1-Ipi3-Ipi1 complex is known to exhibit a large hollow sphere with a diameter of ~9 nm in yeast (Barrio-Garcia et al., 2016; Kater et al., 2020) and the reconstituted ct Las1-Grc3 heterodimer a butterfly-like structure of 7 nm x 13 nm (Pillon et al., 2017), we conclude that we can observe the entire Rixosome by electron microscopy ». The dimensions of the 2D classes should be clearly depicted in the **text and/or figure** so that this conclusion can fully and unambiguously convince readers.

The dimensions of both the Rix1 subcomplex and the Las1-Grc3 were now measured in the negative stain EM 2D classes are indeed highly similar: 10 nm +/- 1 nm for the Rix1 complex and 13 x 8 nm +/- 1 nm for the Las1-Grc1. These values are now depicted in the revised ms.

Cryo-EM processing. The loose (flexible) connection between both Rixosome subcomplexes prevents reaching high resolution for the 3D structures of the full Rixosome particles. Thus, the authors processed the submodules separately, to reach 2.9 - 3.0 A resolution for the Rix1 and Las1-Grc3 subcomplexes, respectively. Could the authors precise whether they find, in their cryo-EM images or in their 2D classes, evidences of the existence of independent submodules (Rix1 alone or Las1-Grc3 alone), and how much of full rixosome vs. isolated submodules they can count ? Or, did they had to systematically mask out one submodule from full rixosome particles to study the other ? This is not entirely clear from the M&M or the processing schemes, and might help to understand the dynamics of interaction between Las1-Grc3 and Rix1 complex.

The Rixosome was affinity purified via Las1 N-terminal tagged with PTF, yet, we observed after several rounds of particle picking a ~ 6 fold excess of Rix1 subcomplexes in the cryo-EM 2D analysis (588,317 particle of the Rix1 complexes vs 106,869 particle of the Las1-Grc3 complexes). This is likely a result of a varying performance of the particle picking algorithms which might work less efficiently for the Las1-Grc3 complex. The Rix1 complex is a rather spherical complex and can be recognized without difficulty in 2D class averages. For the Las1-Grc3 complex the butterfly-like front and back views of the complex are easy to recognize but side views are more difficult to identify and therefore might be overlooked during processing.
Therefore, any conclusions regarding individual subcomplexes vs full Rixosome would be highly problematic. In addition, it is not clear to what extent the Rixosome is prone to dissociation into subcomplexes upon grid application and freezing as observed for many other complexes studied by cryo-EM. The individual high resolution 3D reconstructions of the Las1-Grc3 and the Rix1 complexes were done by processing the complexes individually (see Material and Methods section and Appendix Figure S2). The full complex was then reconstructed by extending the box size of the Las1-Grc3 complex images including the Rix complex densities partially visible in the 2D classes. To that end, we used 38,095 particles of the initially identified 106,869 Grc3-Las1 particles. Due to the long and highly flexible linker region (see Alphafold multimer models in Figure EV1) both complexes are very flexibly orientated to each other. For the reconstruction of the Rixosome we only chose 2D class averages were both complexes were similarly orientated, even though most 2D classes appear to contain both complexes or at least one of the complexes and a fuzzy density for the second complex (see Source Data for 3A). Exact particle numbers for the different processing steps can be found in the sorting scheme (Appendix Figure S2). The image processing is described in detail in the Materials and Method section.

It is not entirely clear how strong the hypothesis regarding the timing of ITS2 maturation compared to 5S RNP rotation is for the authors. On the results part, based on their observations they draw the conclusion that the ITS2 cleavage precedes 5S RNP rotation («Altogether, we conclude that initial ITS2 processing by the Las1-Grc3 rixosome subcomplex likely precedes 5S RNP rotation »). However, and very understandably so, they don't provide direct evidence that ITS2 cleavage and trimming has been initiated by Las1-Grc3. Then, in the discussion part, this assessment is presented as a clear but not entirely proven hypothesis, which is a fairer way to present it : it might be worth reformulating the sentence of the results part as an hypothesis, not an unquestionable conclusion.

As suggested, we have reformulated this sentence of the results part, so that it does no longer read as an unquestionable conclusion. The revised sentence is: “Thus, the relative neighborhood of the Las1-Grc3 subcomplex involved in the ITS2 processing and the Rix1 complex with a facilitating role in 5S RNP rotation potentially allows for the coordination of both processes (see Discussion)”.

Minor point : please harmonize writing for the Rsa4 mutations (E117D, E>117D...)

The writing has been harmonized to E117D.

Dear Ed,

Thank you for the submission of your revised manuscript. There are only some last points that will need to be addressed and clarified before we can proceed with the official acceptance of your manuscript.

Our routine image check of revised manuscripts found potential image re-uses in Appendix Figure S4 B and E. I understand that images in S4 B and E are not expected to be identical? Can you please explain?

Please remove the author credits from the ms file. We now use CRediT to specify the contributions of each author in the journal submission system. CRediT replaces the author contribution section. Please use the free text box to provide more detailed descriptions, if you wish. See also guide to authors
https://www.embopress.org/page/journal/14693178/authorguide#authorshipguidelines.

Some funding info is missing in our online journal submission system, please add the German Research Foundation (HU363/12-2).

Please add a callout for Appendix Table S2 to the ms text.

EMBO press papers are accompanied online by A) a short (1-2 sentences) summary of the findings and their significance, B) 2-3 bullet points highlighting key results and C) a synopsis image that is exactly 550 pixels wide and 200-600 pixels high (the height is variable). You can either show a model or key data in the synopsis image. Please note that text needs to be readable at the final size. Please send us this information along with the final manuscript.

I look forward to seeing a final version of your manuscript as soon as possible.

Best regards,

Esther

Esther Schnapp, PhD
Senior Editor
EMBO reports

Dear Dr. Esther Schnapp,

Thank you very much for your email dated October 10th, 2023, regarding our manuscript EMBOR-2023-57984-T.

Only a few points have been finally raised by you, which we address in the following.

We hope that our manuscript can now be accepted for publication in *EMBO Reports*.

Best regards,

Ed Hurt and Roland Beckmann

Your e-mail from October 10th, 2023

Dear Ed,

Thank you for the submission of your revised manuscript. There are only some last points that will need to be addressed and clarified before we can proceed with the official acceptance of your manuscript.

Our routine image check of revised manuscripts found potential image re-uses in Appendix Figure S4 B and E. I understand that images in S4 B and E are not expected to be identical? Can you please explain?

Our response:
There are no duplications of identical images. The images in question simply represent highly similar averages of particle orientations, however, in one case present in a horizontally flipped orientation on the EM grid. The 2D projections of such particles look the same as soon as their orientation difference is close to a 180 degree rotation perpendicular to the optical axis (in other words: lying on their back vs lying on their face) and can be transformed into each other by a mirroring operation as was done for the image analysis ('horizontally flipped') for every image pair.

Please remove the author credits from the ms file. We now use CRediT to specify the contributions of each author in the journal submission system. CRediT replaces the author contribution section. Please use the free text box to provide more detailed descriptions, if you wish. See also guide to authors https://www.embopress.org/page/journal/14693178/authorguide#authorshipguidelines.

We have removed the author credits from the ms file and await that EMBO Reports adds the contributions via CRediT supplied in the journal submission system.

Some funding info is missing in our online journal submission system, please add the German Research Foundation (HU363/12-2).

We have added this GRF HU363/12-2 funding into the online submission system

Please add a callout for Appendix Table S2 to the ms text.

Appendix Table S2 has been twice added into the ms (marked red):
"Next, we performed cryo-EM of the *ct* rixosome to unravel additional structural details (Appendix Figure S2 and Table S2)."
"….by single particle cryo-EM (Appendix Figures S3-S7 and Table S2)."

EMBO press papers are accompanied online by A) a short (1-2 sentences) summary of the findings and their significance, B) 2-3 bullet points highlighting key results and C) a synopsis image that is exactly 550 pixels wide and 200-600 pixels high (the height is variable). You can either show a model or key data in the synopsis image. Please note that text needs to be readable at the final size. Please send us this information along with the final manuscript.

Both (1) a short (1-2 sentences) summary, (2) 2 bullet points and (3) a synopsis image are now provided

Synopsis

The rixosome is involved in ribosome biogenesis and gene silencing. Its cryo-EM structure explains how it can participate in pre-60S ribosome maturation during 5S RNP rotation and ITS2 processing.

- Rix1 sub-module of the rixosome functions as central hub to trigger the 5S RNP rotation on pre-60S particles
- Las1-Grc3 endonuclease flexibly attached to the rixosome can be strategically positioned at the pre-60S foot to perform ITS2 processing

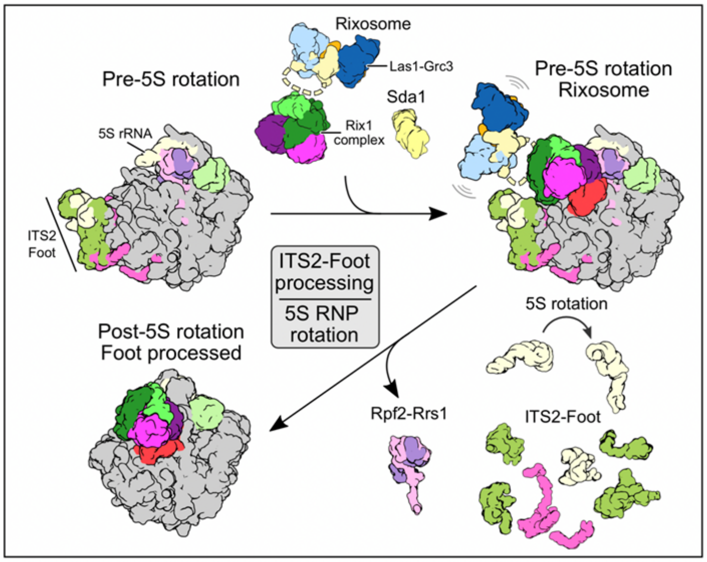

I look forward to seeing a final version of your manuscript as soon as possible. Please use this link to submit your revision: https://embor.msubmit.net/cgi-bin/main.plex

Best regards,

Esther

Esther Schnapp, PhD
Senior Editor
EMBO reports

Prof. Ed Hurt
Heidelberg, Ruprecht-Karls-Universität
BZH - Universität Heidelberg
Im Neuenheimer Feld 328
D-69120 Heidelberg
Heidelberg D-69126
Germany

Dear Prof. Hurt,

I am very pleased to accept your manuscript for publication in the next available issue of EMBO reports. Thank you for your contribution to our journal.

At the end of this email I include important information about how to proceed. Please ensure that you take the time to read the information and complete and return the necessary forms to allow us to publish your manuscript as quickly as possible.

As part of the EMBO publication's Transparent Editorial Process, EMBO reports publishes online a Review Process File to accompany accepted manuscripts. As you are aware, this File will be published in conjunction with your paper and will include the referee reports, your point-by-point response and all pertinent correspondence relating to the manuscript.

If you do NOT want this File to be published, please inform the editorial office within 2 days, if you have not done so already, otherwise the File will be published by default [contact: emboreports@embo.org]. If you do opt out, the Review Process File link will point to the following statement: "No Review Process File is available with this article, as the authors have chosen not to make the review process public in this case."

Thank you again for your contribution to EMBO reports and congratulations on a successful publication. Please consider us again in the future for your most exciting work.

Yours sincerely,

Esther Schnapp, PhD
Senior Editor
EMBO reports

****

THINGS TO DO NOW:

Please note that you will be contacted by Wiley Author Services to complete licensing and payment information. The required 'Page Charges Authorization Form' is available here: https://www.embopress.org/pb-assets/embo-site/er_apc.pdf - please download and complete the form and return to embopressproduction@wiley.com

EMBO Press participates in many Publish and Read agreements that allow authors to publish Open Access with reduced/no publication charges. Check your eligibility: https://authorservices.wiley.com/author-resources/Journal-Authors/open-access/affiliation-policies-payments/index.html

You will receive proofs by e-mail approximately 2-3 weeks after all relevant files have been sent to our Production Office; you should return your corrections within 2 days of receiving the proofs.

Please inform us if there is likely to be any difficulty in reaching you at the above address at that time. Failure to meet our deadlines may result in a delay of publication, or publication without your corrections.

All further communications concerning your paper should quote reference number EMBOR-2023-57984V3 and be addressed to emboreports@wiley.com.

Should you be planning a Press Release on your article, please get in contact with emboreports@wiley.com as early as possible, in order to coordinate publication and release dates.

EMBO Press Author Checklist

Corresponding Author Name: Ed Hurt	
Journal Submitted to: EMBO Reports	
Manuscript Number: EMBOR-2023-57984-T	

USEFUL LINKS FOR COMPLETING THIS FORM
The EMBO Journal - Author Guidelines
EMBO Reports - Author Guidelines
Molecular Systems Biology - Author Guidelines
EMBO Molecular Medicine - Author Guidelines

Reporting Checklist for Life Science Articles (updated January

This checklist is adapted from Materials Design Analysis Reporting (MDAR) Checklist for Authors. MDAR establishes a minimum set of requirements in transparent reporting in the life sciences (see Statement of Task: 10.31222/osf.io/9sm4x). Please follow the journal's guidelines in preparing your

Please note that a copy of this checklist will be published alongside your article.

Abridged guidelines for figures

1. Data

The data shown in figures should satisfy the following conditions:

- the data were obtained and processed according to the field's best practice and are presented to reflect the results of the experiments in an accurate and unbiased manner.
- ideally, figure panels should include only measurements that are directly comparable to each other and obtained with the same assay.
- plots include clearly labeled error bars for independent experiments and sample sizes. Unless justified, error bars should not be shown for technical
- if n<5, the individual data points from each experiment should be plotted. Any statistical test employed should be justified.
- Source Data should be included to report the data underlying figures according to the guidelines set out in the authorship guidelines on Data

2. Captions

Each figure caption should contain the following information, for each panel where they are relevant:

- a specification of the experimental system investigated (eg cell line, species name).
- the assay(s) and method(s) used to carry out the reported observations and measurements.
- an explicit mention of the biological and chemical entity(ies) that are being measured.
- an explicit mention of the biological and chemical entity(ies) that are altered/varied/perturbed in a controlled manner.
- the exact sample size (n) for each experimental group/condition, given as a number, not a range;
- a description of the sample collection allowing the reader to understand whether the samples represent technical or biological replicates (including how many animals, litters, cultures, etc.).
- a statement of how many times the experiment shown was independently replicated in the laboratory.
- definitions of statistical methods and measures:
  - common tests, such as t-test (please specify whether paired vs. unpaired), simple χ2 tests, Wilcoxon and Mann-Whitney tests, can be unambiguously identified by name only, but more complex techniques should be described in the methods section;
  - are tests one-sided or two-sided?
  - are there adjustments for multiple comparisons?
  - exact statistical test results, e.g., P values = x but not P values < x;
  - definition of 'center values' as median or average;
  - definition of error bars as s.d. or s.e.m.

Please complete ALL of the questions below.
Select "Not Applicable" only when the requested information is not relevant for your study.

Materials

**Newly Created Materials**	**Information included in the manuscript?**	**In which section is the information available?** (Reagents and Tools Table, Materials and Methods, Figures, Data Availability Section)
New materials and reagents need to be available; do any restrictions apply?	Not Applicable	

**Antibodies**	**Information included in the manuscript?**	**In which section is the information available?** (Reagents and Tools Table, Materials and Methods, Figures, Data Availability Section)
For **antibodies** provide the following information:<br>- Commercial antibodies: RRID (if possible) or supplier name, catalogue number and or/clone number<br>- Non-commercial: RRID or citation	Not Applicable	

**DNA and RNA sequences**	**Information included in the manuscript?**	**In which section is the information available?** (Reagents and Tools Table, Materials and Methods, Figures, Data Availability Section)
**Short novel DNA or RNA including primers, probes**: provide the sequences.	Not Applicable	

**Cell materials**	**Information included in the manuscript?**	**In which section is the information available?** (Reagents and Tools Table, Materials and Methods, Figures, Data Availability Section)
**Cell lines:** Provide species information, strain. Provide accession number in repository **OR** supplier name, catalog number, clone number, and/**OR** RRID.	Not Applicable	
**Primary cultures:** Provide species, strain, sex of origin, genetic modification status.	Yes	Materials and Methods, Appendix Table S1
Report if the cell lines were recently **authenticated** (e.g., by STR profiling) and tested for mycoplasma contamination.	Not Applicable	

**Experimental animals**	**Information included in the manuscript?**	**In which section is the information available?** (Reagents and Tools Table, Materials and Methods, Figures, Data Availability Section)
**Laboratory animals or Model organisms:** Provide species, strain, sex, age, genetic modification status. Provide accession number in repository **OR** supplier name, catalog number, clone number, **OR** RRID.	Yes	Materials and Methods, Appendix Table S1
**Animal observed in or captured from the field:** Provide species, sex, and age where possible.	Not Applicable	
Please detail **housing and husbandry conditions**.	Not Applicable	

**Plants and microbes**	**Information included in the manuscript?**	**In which section is the information available?** (Reagents and Tools Table, Materials and Methods, Figures, Data Availability Section)
**Plants:** provide species and strain, ecotype and cultivar where relevant, unique accession number if available, and source (including location for collected wild specimens).	Not Applicable	
**Microbes:** provide species and strain, unique accession number if available, and source.	Not Applicable	

**Human research participants**	**Information included in the manuscript?**	**In which section is the information available?** (Reagents and Tools Table, Materials and Methods, Figures, Data Availability Section)
If collected and within the bounds of privacy constraints report on age, sex and gender or ethnicity for all study participants.	Not Applicable	

**Core facilities**	**Information included in the manuscript?**	**In which section is the information available?** (Reagents and Tools Table, Materials and Methods, Figures, Data Availability Section)
If your work benefited from core facilities, was their service mentioned in the acknowledgments section?	Not Applicable	

Design

**Study protocol**	**Information included in the manuscript?**	**In which section is the information available?** (Reagents and Tools Table, Materials and Methods, Figures, Data Availability Section)

If study protocol has been **pre-registered, provide DOI in the manuscript**. For clinical trials, provide the trial registration number **OR** cite DOI.	Not Applicable	
Report the **clinical trial registration number** (at ClinicalTrials.gov or equivalent), where applicable.	Not Applicable	

Laboratory protocol	Information included in the manuscript?	In which section is the information available? (Reagents and Tools Table, Materials and Methods, Figures, Data Availability Section)
Provide DOI OR other citation details if **external detailed step-by-step protocols** are available.	Not Applicable	

Experimental study design and statistics	Information included in the manuscript?	In which section is the information available? (Reagents and Tools Table, Materials and Methods, Figures, Data Availability Section)
Include a statement about **sample size** estimate even if no statistical methods were used.	Yes	Materials and Methods, Figures
Were any steps taken to minimize the effects of subjective bias when allocating animals/samples to treatment (e.g. **randomization procedure**)? If yes, have they been described?	Not Applicable	
Include a statement about **blinding** even if no blinding was done.	Not Applicable	
Describe **inclusion/exclusion criteria** if samples or animals were excluded from the analysis. Were the criteria pre-established?<br><br>If sample or data points were omitted from analysis, report if this was due to attrition or intentional exclusion and provide justification.	Not Applicable	
For every figure, are **statistical tests** justified as appropriate? Do the data meet the assumptions of the tests (e.g., normal distribution)? Describe any methods used to assess it. Is there an estimate of variation within each group of data? Is the variance similar between the groups that are being statistically compared?	Not Applicable	

Sample definition and in-laboratory replication	Information included in the manuscript?	In which section is the information available? (Reagents and Tools Table, Materials and Methods, Figures, Data Availability Section)
In the figure legends: state number of times the experiment was **replicated** in laboratory.	Yes	Figure legends
In the figure legends: define whether data describe **technical or biological replicates**.	Not Applicable	

Ethics

Ethics	Information included in the manuscript?	In which section is the information available? (Reagents and Tools Table, Materials and Methods, Figures, Data Availability Section)
Studies involving **human participants**: State details of **authority granting ethics approval** (IRB or equivalent committee(s), provide reference number for approval.	Not Applicable	
Studies involving **human participants**: Include a statement confirming that **informed consent** was obtained from all subjects and that the experiments conformed to the principles set out in the WMA Declaration of Helsinki and the Department of Health and Human Services Belmont Report.	Not Applicable	
Studies involving **human participants:** For publication of **patient photos**, include a statement confirming that consent to publish was obtained.	Not Applicable	
Studies involving experimental **animals**: State details of **authority granting ethics approval** (IRB or equivalent committee(s), provide reference number for approval. Include a statement of compliance with ethical regulations.	Not Applicable	
Studies involving **specimen and field samples:** State if relevant **permits** obtained, provide details of authority approving study; if none were required, explain why.	Not Applicable	

Dual Use Research of Concern (DURC)	Information included in the manuscript?	In which section is the information available? (Reagents and Tools Table, Materials and Methods, Figures, Data Availability Section)
Could your study fall under dual use research restrictions? Please check biosecurity documents and list of **select agents and toxins** (CDC): https://www.selectagents.gov/sat/list.htm	Not Applicable	
If you used a select agent, is the security level of the lab appropriate and reported in the manuscript?	Not Applicable	
If a study is subject to dual use research of concern regulations, is the name of the **authority granting approval and reference number** for the regulatory approval provided in the manuscript?	Not Applicable	

Reporting

The MDAR framework recommends adoption of discipline-specific guidelines, established and endorsed through community initiatives. Journals have their own policy about requiring specific guidelines and recommendations to complement MDAR.

Adherence to community standards	Information included in the manuscript?	In which section is the information available? (Reagents and Tools Table, Materials and Methods, Figures, Data Availability Section)
State if relevant guidelines or checklists (e.g., **ICMJE, MIBBI, ARRIVE, PRISMA**) have been followed or provided.	Not Applicable	
For **tumor marker prognostic studies,** we recommend that you follow the **REMARK** reporting guidelines (see link list at top right). See author guidelines, under 'Reporting Guidelines'. Please confirm you have followed these guidelines.	Not Applicable	
For **phase II and III randomized controlled trials**, please refer to the **CONSORT** flow diagram (see link list at top right) and submit the CONSORT checklist (see link list at top right) with your submission. See author guidelines, under 'Reporting Guidelines'. Please confirm you have submitted this list.	Not Applicable	

Data Availability

Data availability	Information included in the manuscript?	In which section is the information available? (Reagents and Tools Table, Materials and Methods, Figures, Data Availability Section)
Have **primary datasets** been deposited according to the journal's guidelines (see 'Data Deposition' section) and the respective accession numbers provided in the Data Availability Section?	Yes	Data Availability Section
Were **human clinical and genomic datasets** deposited in a public access-controlled repository in accordance to ethical obligations to the patients and to the applicable consent agreement?	Not Applicable	
Are **computational models** that are central and integral to a study available without restrictions in a machine-readable form? Were the relevant accession numbers or links provided?	Not Applicable	
If publicly available data were reused, provide the respective **data citations in the reference list**.	Not Applicable	
